# Correction: Mukavi et al. Antiprotozoal Aminosteroid Alkaloids from *Buxus obtusifolia* (Mildbr.) Hutch. *Molecules* 2025, *30*, 4558

**DOI:** 10.3390/molecules31111807

**Published:** 2026-05-25

**Authors:** Justus Wambua Mukavi, Monica Cal, Marcel Kaiser, Pascal Mäser, Njogu M. Kimani, Leonidah Kerubo Omosa, Thomas J. Schmidt

**Affiliations:** 1 University of Münster, Institute of Pharmaceutical Biology and Phytochemistry (IPBP), PharmaCampus, Corrensstraße 48, D-48149 Münster, Germany; jmukavi@uni-muenster.de; 2 Swiss Tropical and Public Health Institute (Swiss TPH), Kreuzstrasse 2, CH-4123 Allschwil, Switzerland; monica.cal@swisstph.ch (M.C.); marcel.kaiser@swisstph.ch (M.K.); pascal.maeser@swisstph.ch (P.M.); 3 University of Basel, Petersplatz 1, CH-4003 Basel, Switzerland; 4 Department of Physical Sciences, University of Embu, Embu P.O. Box 6-60100, Kenya; mark.njogu@gmail.com; 5 Department of Chemistry, Faculty of Science & Technology, University of Nairobi, Nairobi P.O. Box 30197-00100, Kenya; lkerubo@uonbi.ac.ke

## Figure Legend

In the original publication [1], there was a mistake in the legend for Figure 1. The scientific name was not italicized. The correct legend appears below.

**Figure 1.** Chemical structures of alkaloids isolated from *Buxus obtusifolia*. New compounds are marked by an asterisk (*).

## Table Legend

In the original publication [1], there was a mistake in the legend for Table 8. The superscripts ^a^ and ^b^, indicating which of the compounds were tested as mono-TFA and bis-TFA salts, were mistakenly exchanged. The correct legend appears below.

**Table 8 caption.** Antiprotozoal activity and cytotoxicity of compounds isolated from *Buxus obtusifolia* leaves. Data are the means of two separate IC_50_ determinations with absolute deviations from the mean. Compounds were tested as the *bis* ^a^- or mono ^b^-trifluoroacetates.

**Table 8 footnote.** * Deviation not calculable as the result of one replicate was higher than the maximal concentration tested (>100 µg/mL). ^a^ *bis*-trifluoroacetate; ^b^ mono-trifluoroacetate; ^c^ alkaloids isolated as a mixture. Positive controls: melarsoprol (*Tbr*), chloroquine (*Pf*), podophyllotoxin (Cytotox.L6); selectivity indices (SI) = cytotoxic IC_50_/IC_50_ of the target parasite.

## Error in Tables

In the original publication [1], there was a mistake in Table 1 and Table 2 as published. There were inconsequential rounding errors in some selectivity index (SI) values. The corrected Table 1 and Table 2 appear below. 

**Table 1:** Preliminary in vitro antiprotozoal and cytotoxic activity of the crude extracts from the leaves of *Buxus obtusifolia*, and the acid-base extraction fractions. Data (in µg/mL) are the means of two separate IC_50_ determinations ± the absolute deviations from the mean.

Test Sample	*Tbr*	*Pf*	Cytotox. L6	SI (*Tbr*)	SI (*Pf*)
Small-scale extracts					
Twigs (CH_3_OH)	6.3 ± 0.04	2.8 ± 0.25	54 ± 2.4	8.6	19
Twigs (CH_2_Cl_2_)	12 ± 0.40	3.1 ± 0.33	57 ± 4.7	4.8	18
Leaves (CH_3_OH)	13 ± 0.25	2.3 ± 0.33	51 ± 2.9	3.9	22
Leaves (CH_2_Cl_2_)	16 ± 0.45	1.1 ± 0.11	26 ± 1.4	1.6	24
Fractions of the large-scale CH_2_Cl_2_ extract					
Alkaloid fraction	8.1 ± 3.1	0.69 ± 0.14	42 ± 4.2	5.2	60.9
Lipophilic fraction	14 ± 0.90	2.2 ± 0.10	50 ± 0.7	3.5	23
Hydrophilic residue	>100	>50	>100	-	-
Positive controls	0.004 ± 0.000 ^a^	0.002 ± 0.000 ^b^	0.007 ± 0.002 ^c^	-	-

Positive controls: ^a^ melarsoprol (*Tbr*), ^b^ chloroquine (*Pf*), ^c^ podophyllotoxin (Cytotoxic L6); selectivity indices (SI) = IC_50_ (Cytotox. L6)/IC_50_ (parasite).

**Table 2:** Antiprotozoal activity of centrifugal partition chromatography (CPC) subfractions and positive controls. Data (in µg/mL) are the means of two separate IC_50_ determinations ± the absolute deviations from the mean.

Test Sample	*Tbr*	*Pf*	Cytotox.L6	SI *Tbr*	SI *Pf*
Fr. 01	22 ± 0.6	4.2 ± 0.6	67 ± 14	3	16
Fr. 02	42 ± 5.3	7.6 ± 1.4	58 ^a^	1	8
Fr. 03	0.8 ± 0.0	1.9 ± 0.5	26 ± 9.3	33	14
Fr. 04	0.8 ± 0.0	1.6 ± 0.6	16 ± 2.5	20	10
Fr. 05	4.4 ± 0.2	1.0 ± 0.2	15 ± 3.0	3	15
Fr. 06	2.4 ± 0.3	1.1 ± 0.2	14 ± 3.2	6	13
Fr. 07	2.9 ± 0.2	1.0 ± 0.1	15 ± 2.1	5	15
Fr. 08	4.2 ± 0.2	1.1 ± 0.2	14 ± 2.4	3	13
Fr. 09	2.9 ± 0.5	1.1 ± 0.2	12 ± 3.2	4	11
Fr. 10a	4.2 ± 0.3	1.2 ± 0.1	16 ± 1.3	4	13
Fr. 10b	3.6 ± 0.6	1.7 ± 0.5	14 ± 4.1	4	8
Fr. 11	3.4 ± 0.0	1.8 ± 0.4	12 ± 3.2	4	7
Fr. 12	3.3 ± 0.5	1.7 ± 0.4	18 ± 4.4	5	11
Fr. 13	2.9 ± 0.2	1.8 ± 0.6	16 ± 3.6	6	9
Fr. 14	5.2 ± 0.4	1.1 ± 0.2	18 ± 0.4	4	16
Fr. 15	5.2 ± 0.2	0.9 ± 0.1	17 ± 0.5	3	19
Fr. 16	17 ± 0.5	1.1 ± 0.5	28 ± 12	2	25
Positive controls	0.004 ± 0.000 ^b^	0.002 ± 0.000 ^c^	0.007 ± 0.003 ^d^	-	-

^a^ Deviation not calculable as the result of one replicate was higher than the maximal concentration tested (>100 µg/mL). Positive controls: ^b^ melarsoprol (*Tbr*), ^c^ chloroquine (*Pf*), ^d^ podophyllotoxin (Cytotox.L6); selectivity indices (SI) = IC_50_ (Cytotox. L6)/IC_50_ (parasite).

In the original publication [1], there was a mistake in Table 8 as published. There was an additional superscript c to indicate that compounds **8a** + **8b** and **9a** + **9b** were isolated as a mixture. The superscript b, indicating that these compounds were tested as mono-TFA salts, was missing and has been added. The corrected Table 8 appears below. 

**Table 8:** Antiprotozoal activity and cytotoxicity of compounds isolated from *Buxus obtusifolia* leaves. Data are the means of two separate IC_50_ determinations with absolute deviations from the mean. Compounds were tested as the *bis* ^a^- or mono ^b^-trifluoroacetates.

Compound	*Tbr* [µmol/L]	*Pf* [µmol/L]	Cytotox.L6 [µmol/L]	SI *Tbr*	SI *Pf*
**1** ^a^	6.7 ± 3.3	28 ± 0.7	>100	-	-
**2** ^a^	3.8 ± 0.1	9.5 ± 0.9	96 ± 5.0	25	10
**3** ^a^	7.3 ± 0.1	16 ± 2.1	107 ± 34	15	7
**4** ^a^	2.3 ± 0.3	1.1 ± 0.0	16 ± 1.9	7	15
**5** ^a^	1.6 ± 0.7	0.5 ± 0.0	5.9 ± 0.0	4	12
**6** ^a^	0.9 ± 0.2	3.0 ± 0.1	50 ± 11	56	17
**7** ^a^	48 ± 8.2	49 ± 3.9	78 *	2	2
**8a** + **8b** ^b,c^	41 ± 0.6	30 ± 1.8	52 ± 22	1	2
**9a** + **9b** ^b,c^	39 ± 2.0	26 ± 0.4	68 ± 0.7	2	3
**10** ^a^	37 ± 18	20 ± 1.3	51 ± 2.8	1	3
**11** ^a^	14 ± 7.0	19 ± 1.1	50 ± 1.6	4	3
**12** ^a^	0.8 ± 0.3	5.4 ± 1.2	82 ± 4.1	103	15
**13** ^a^	11 ± 0.3	8.4 ± 0.9	77 ± 4.8	7	9
**14** ^a^	16 ± 0.5	11 ± 2.2	57 ± 1.2	4	5
**15** ^a^	6.6 ± 3.5	5.8 ± 0.4	24 ± 1.5	4	4
**16** ^b^	22 ± 1.7	13 ± 1.4	45 ± 5.1	2	3
**17** ^b^	44 ± 16	11 ± 2.2	50 ± 0.5	1	5
**18** ^b^	4.9 ± 0.1	17 ± 1.5	27 ± 1.7	5	2
**19** ^b^	2.0 ± 0.2	18 ± 0.8	16 ± 1.1	8	1
**20** ^b^	23 ± 4.7	8.7 ± 2.8	43 ± 2.0	2	5
**21** ^b^	2.2 ± 0.2	10 ± 1.1	34 ± 0.5	16	3
**22** ^b^	6.5 ± 0.3	24 ± 2.7	48 ± 2.2	7	2
**23** ^b^	6.5 ± 0.4	30 ± 6.5	39 ± 5.9	7	1
**24** ^b^	26 ± 1.2	6.4 ± 0.2	51 ± 1.6	2	8
Positive controls	0.013 ± 0.001	0.012 ± 0.000	0.014 ± 0.004	-	-

* Deviation not calculable as the result of one replicate was higher than the maximal concentration tested (>100 µg/mL). ^a^ *bis*-trifluoroacetate; ^b^ mono-trifluoroacetate; ^c^ alkaloids isolated as a mixture. Positive controls: melarsoprol (*Tbr*), chloroquine (*Pf*), podophyllotoxin (Cytotox.L6); selectivity indices (SI) = cytotoxic IC_50_/IC_50_ of the target parasite.

## Text Correction

There was an error in the original publication [1]. In the abstract, the selectivity index (SI) values reported for deoxycyclovirobuxeine-B (**12**) and 29-trimethoxybenzoyloxy cycloprotobuxoline-C (**5**) were stated incorrectly. Additionally, the name for compound **5** was incorrectly spelled.

A correction has been made to the second-to-last sentence of the Abstract:

Deoxycyclovirobuxeine-B (**12**) (IC_50_ = 0.8 µmol/L, SI = 103) and 29-trimethoxybenzoyloxy cycloprotobuxoline-C (**5**) (IC_50_ = 0.5 µmol/L, SI = 12) showed the highest activities with good selectivity indices against *Tbr* and *Pf*, respectively.

There was an error in the original publication [1]. In the heading of Section 2.2, the first letter of the species name was incorrectly capitalized. In addition, the two SI values referring to CPC subfractions 3 and 4 contained inconsequential calculation errors, which have now been corrected in accordance with the updated Table 2.

A correction has been made to the heading of Section 2.2, and Paragraph 1 of this section:

*2.2.* *Isolation and Characterization of Aminosteroids from Buxus obtusifolia*

Furthermore, subfractions 3 and 4 demonstrated significant activity against *Tbr* (IC_50_ < 1 μg/mL) with greater selectivity indices (SI = 33 and 20, respectively) compared to the parent alkaloidal fraction.

There was an error in the original publication [1]. In Section 2.2, paragraph 9, the names of compounds **8a** and **8b** were incorrectly spelled.

A correction has been made to the last sentence on paragraph 9:

Based on the above-mentioned data, and the relation to compound **4**, compounds **8a** and **8b** were assigned as cycloprotobuxoline-D *N*_3_-*trans*- and cycloprotobuxoline-D *N*_3_-*cis*-formamide, respectively.

There was an error in the original publication [1]. In Section 2.2, paragraphs 13, 14 and 19, the names of compounds **15**, **16** and **21** were lacking hyphens to be consistent with the other compound names. A correction has been made to the names of these compounds:

*N*_20_-demethyl deoxycyclobuxoxazine-A, obtusibuxeine-A, and obtusiepoxamine-A.

There was an error in the original publication [1]. In Section 2.3, Paragraph 1, the selectivity index (SI) values reported for compounds **6** and **12**, as well as the IC_50_ value for **2,** were stated incorrectly.

A correction has been made to the last two sentences of this paragraph:

Among the 9,19-cyclo-5α-pregnanes (**1**–**16**), the new compounds **6** and **12** demonstrated the highest antitrypanosomal activity with IC_50_ values of 0.9 µmol/L and 0.8 µmol/L and SI values of 56 and 103, respectively. Albeit with moderate potency, the N-oxide derivative (compound **2**), demonstrated 2-fold the antitrypanosomal activity (IC_50_ = 3.8 µmol/L vs. IC_50_ = 6.7 µmol/L) and 3-fold the antiplasmodial activity (IC_50_ = 9.5 µmol/L vs. IC_50_ = 28 µmol/L) as compared to the unoxidized parent compound **1**.

There was an error in the original publication [1]. In Section 2.3, paragraph 3, the IC_50_ units for compound **4** were stated incorrectly. A correction has been made to the first sentence of this paragraph:

Remarkable antiplasmodial activities were recorded for compounds **4** (IC_50_ = 1.1 µmol/L) and, especially **5** (IC_50_ = 0.5 µmol/L) with the trimethoxy benzoate moiety.

There was an error in the original publication [1]. In Section 3.4, paragraph 3, the name Groß-Umstadt was incorrectly spelled. A correction has been made to the first sentence of this paragraph:

Circular dichroism (CD) spectra of compounds **5**, **17**, **18**, **19**, **21**, **22** and **24** were measured with a Jasco J-815 CD spectrometer (Jasco, Groß-Umstadt, Germany).

There was an error in the original publication [1]. In Section 3.5, paragraphs 7, 10, 11 and 17, the names of compounds **9a** + **9b**, **15**, **16** and **23** were incorrectly spelled. A correction has been made to the names of these compounds:

16α-Hydroxycycloprotobuxoline-C *N*_3_-*trans*-formamide (**9a**) and 16α-hydroxycycloprotobuxoline-C *N*_3_-*cis*-formamide (**9b**), *N*_20_-Demethyl deoxycyclobuxoxazine-A (**15**), Obtusibuxeine-A (**16**) and 16-Deoxyobtusidienolamine-A (**23**).

The authors state that the scientific conclusions are unaffected. This correction was approved by the Academic Editor. The original publication has also been updated.
