# Supplementary material for: Antiprotozoal Aminosteroid Alkaloids from Buxus obtusifolia (Mildbr.) Hutch
Source: Molecules. 2025 Nov 26;30(23):4558. doi: 10.3390/molecules30234558 (PMC12693156; doi:10.3390/molecules30234558)
Supplement: Supplementary file 1 [file molecules-30-04558-s001.zip › molecules-3985427-supplementary.pdf]

Supplementary Materials

Antiprotozoal Aminosteroid Alkaloids from *Buxus obtusifolia* (Mildbr.) Hutch

Justus Wambua Mukavi[1], Monica Cal[2,3], Marcel Kaiser[2,3], Pascal Mäser[2,3], Njogu M. Kimani[4], Leonidah Kerubo Omosa[5], Thomas J. Schmidt[1,*]

1 University of Münster, Institute of Pharmaceutical Biology and Phytochemistry (IPBP), PharmaCampus, Corrensstraße 48, D-48149 Münster, Germany; jmukavi@uni-muenster.de
2 Swiss Tropical and Public Health Institute (Swiss TPH), Kreuzstrasse 2, CH-4123 Allschwil, Switzerland; monica.cal@swisstph.ch (M.C.); marcel.kaiser@swisstph.ch (M.K.); pascal.maeser@swisstph.ch (P.M.)
3 University of Basel, Petersplatz 1, CH-4003 Basel, Switzerland
4 Department of Physical Sciences, University of Embu, P.O. Box 6-60100, Embu, Kenya; mark.njogu@gmail.com (N.M.K.)
5 Department of Chemistry, Faculty of Science & Technology, University of Nairobi, P.O. Box 30197-00100, Nairobi, Kenya; lkerubo@uonbi.ac.ke
* Correspondence: thomschm@uni-muenster.de; Tel.: +49-251-83-33378

Figure S1-S10: Spectral data (LC/MS, ^{1}H NMR, ^{13}C NMR, 2D NMR (HSQC, COSY, HMBC, NOESY)) of cycloprotobuxoline-C N_{20}-oxide (**2**).

Figure S11-S21: Spectral data (LC/MS, ^{1}H NMR, ^{13}C NMR, 2D NMR (HSQC, COSY, HMBC, NOESY)) of 16α-hydroxycycloprotobuxoline-C (**3**).

Figure S22-S34: Spectral data (CD, UV, LC/MS, ^{1}H NMR, ^{13}C NMR, 2D NMR (HSQC, COSY, HMBC, NOESY)) of 29-trimethoxybenzoyloxy cycloprotobuxoline-C (**5**).

Figure S35-S44: Spectral data (LC/MS, ^{1}H NMR, ^{13}C NMR, 2D NMR (HSQC, COSY, HMBC, NOESY)) of N_{3}-demethylcycloprotobuxoline-C (**6**).

Figure S45-S55: Spectral data (LC/MS, ^{1}H NMR, ^{13}C NMR, 2D NMR (HSQC, COSY, HMBC, NOESY)) of 16α hydroxy-N_{3}-demethylcycloprotobuxoline-C (**7**).

Figure S56-S66: Spectral data (^{1}H NMR, ^{13}C NMR, 2D NMR (HSQC, COSY, HMBC, NOESY)) of cycloprotobuxoline-D N_{3}-*trans*-formamide (**8a**) and cycloprotobuxoline-D N_{3}-*cis*-formamide (**8b**).

Figure S67-S80: Spectral data (LC/MS, ^{1}H NMR, ^{13}C NMR, 2D NMR (HSQC, COSY, HMBC, NOESY)) of 16α-hydroxycycloprotobuxoline-C N_{3}-*trans*-formamide (**9a**) and 16α-hydroxycycloprotobuxoline-C N_{3}-*cis*-formamide (**9b**).

Figure S81-S92: Spectral data (LC/MS, ^{1}H NMR, ^{13}C NMR, 2D NMR (HSQC, COSY, HMBC, NOESY)) of N_{3}-demethyl cyclonataminol (**11**).

Figure S93-S105: Spectral data (LC/MS, ^{1}H NMR, ^{13}C NMR, 2D NMR (HSQC, COSY, HMBC, NOESY)) of deoxycyclovirobuxeine-B (**12**).

Figure S106-S118: Spectral data (LC/MS, ^{1}H NMR, ^{13}C NMR, 2D NMR (HSQC, COSY, HMBC, NOESY)) of N_{20}-demethyl deoxycyclobuxoxazine A (**15**).

Figure S119-S132: Spectral data (LC/MS, ^{1}H NMR, ^{13}C NMR, 2D NMR (HSQC, COSY, HMBC, NOESY)) of obtusibuxeine A (**16**).

Figure S133-S144: Spectral data (CD, UV, LC/MS, ^{1}H NMR, ^{13}C NMR, 2D NMR (HSQC, COSY, HMBC, NOESY)) of O^{10}-obtusifuranamine-A (**17**).

Figure S145-S157: Spectral data (CD, UV, LC/MS, ^{1}H NMR, ^{13}C NMR, 2D NMR (HSQC, COSY, HMBC, NOESY)) of O^{10}-obtusifuranamine-B (**18**).

Figure S158-S170: Spectral data (CD, UV, LC/MS, ^{1}H NMR, ^{13}C NMR, 2D NMR (HSQC, COSY, HMBC, NOESY)) of 16-deoxy-O^{10}-obtusifuranamine-B (**19**).

Figure S171-S183: Spectral data (CD, UV, LC/MS, ^{1}H NMR, ^{13}C NMR, 2D NMR (HSQC, COSY, HMBC, NOESY)) of obtusiepoxamine-A (**21**).

Figure S184-S197: Spectral data (CD, UV, LC/MS, ^{1}H NMR, ^{13}C NMR, 2D NMR (HSQC, COSY, HMBC, NOESY)) of obtusidienolamine-A (**22**).

Figure S198-S209: Spectral data (LC/MS, ^{1}H NMR, ^{13}C NMR, 2D NMR (HSQC, COSY, HMBC, NOESY)) of deoxyobtusidienolamine-A (**23**).

Figure S210-S224: Spectral data (CD, UV, LC/MS, ^{1}H NMR, ^{13}C NMR, 2D NMR (HSQC, COSY, HMBC, NOESY)) of obtusiaminocyclin (**24**).

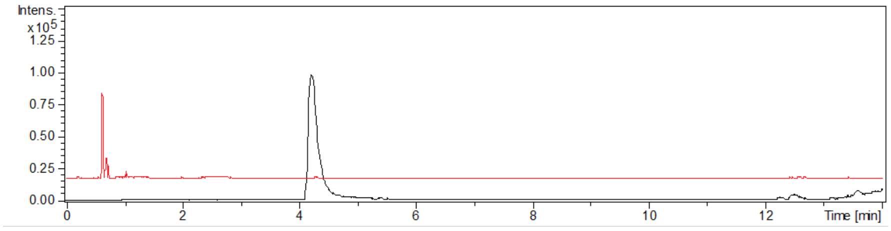

Figure S1. UHPLC/+ESI-QqTOF-MS/MS chromatogram of cycloprotobuxoline-C *N*$_{20}$-oxide (**2**);
Base peak chromatogram 200.0000–1000.0000 +All MS (black); UV chromatogram 200-400 nm (red).

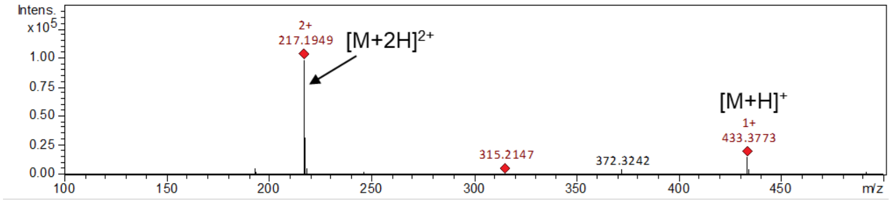

Figure S2. +ESI-QqTOF-MS spectrum of cycloprotobuxoline-C *N*$_{20}$-oxide (**2**); *m/z* 433.3773 $[M+H]^+$, 217.1949 $[M+2H]^{2+}$

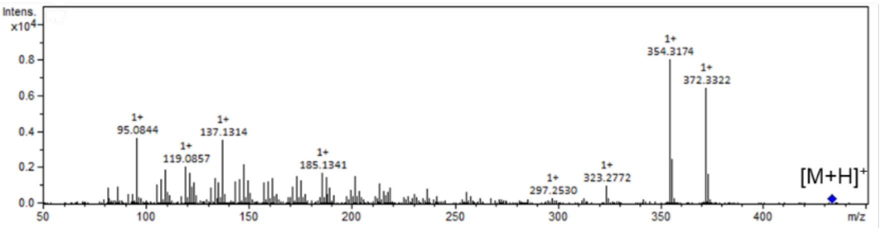

Figure S3. +ESI-QqTOF MS/MS spectrum of cycloprotobuxoline-C *N*$_{20}$-oxide (**2**).

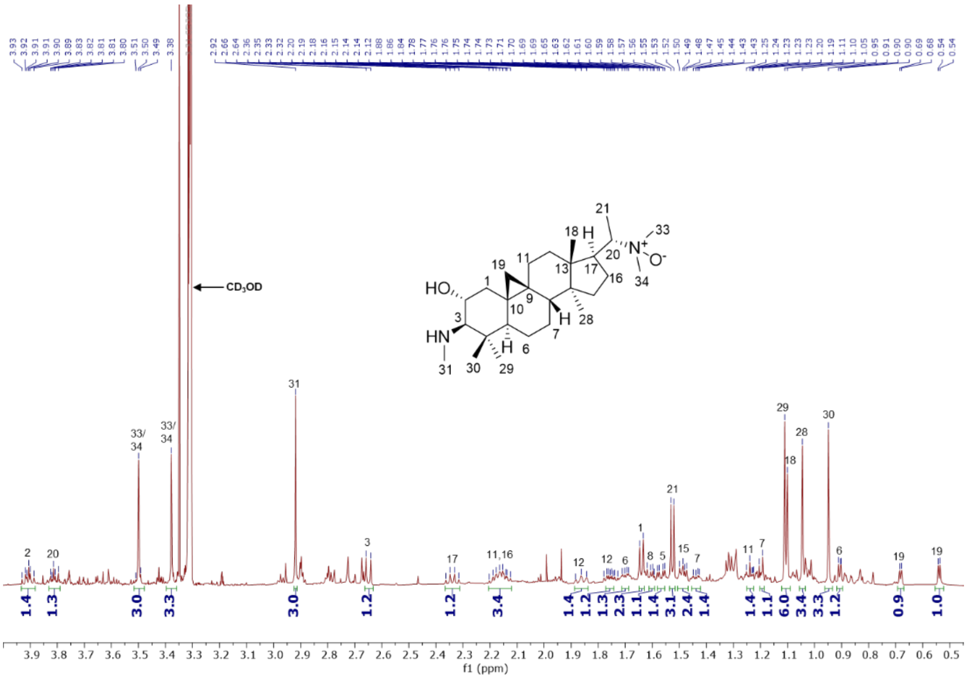

Figure S4. ^{1}H NMR spectrum of cycloprotobuxoline-C *N*$_{20}$-oxide (**2**) (CD_3OD, 600 MHz).

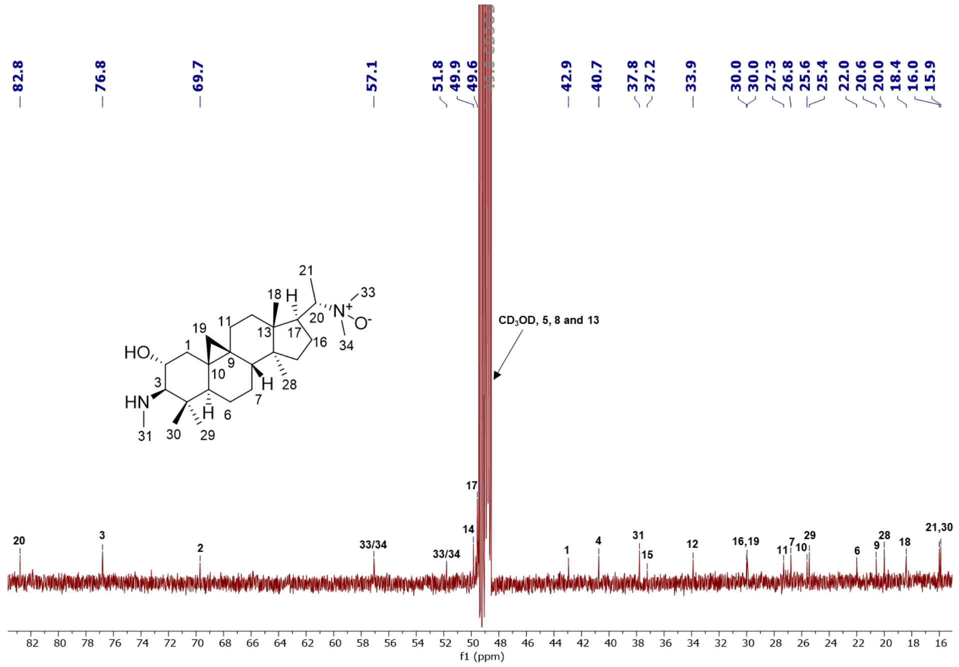

Figure S5. ^{13}C NMR spectrum of cycloprotobuxoline-C *N*$_{20}$-oxide (**2**) (CD_3OD, 150 MHz).

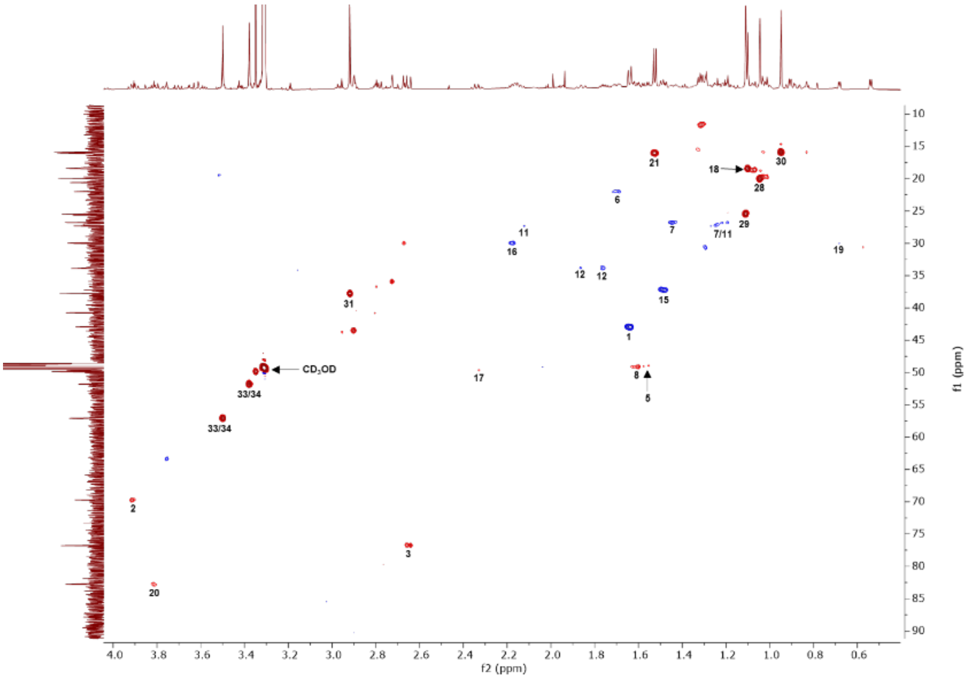

Figure S6. $^{1}H/^{13}C$ HSQC spectrum of cycloprotobuxoline-C *N*$_{20}$-oxide (**2**) (CD_3OD, 600/150 MHz).

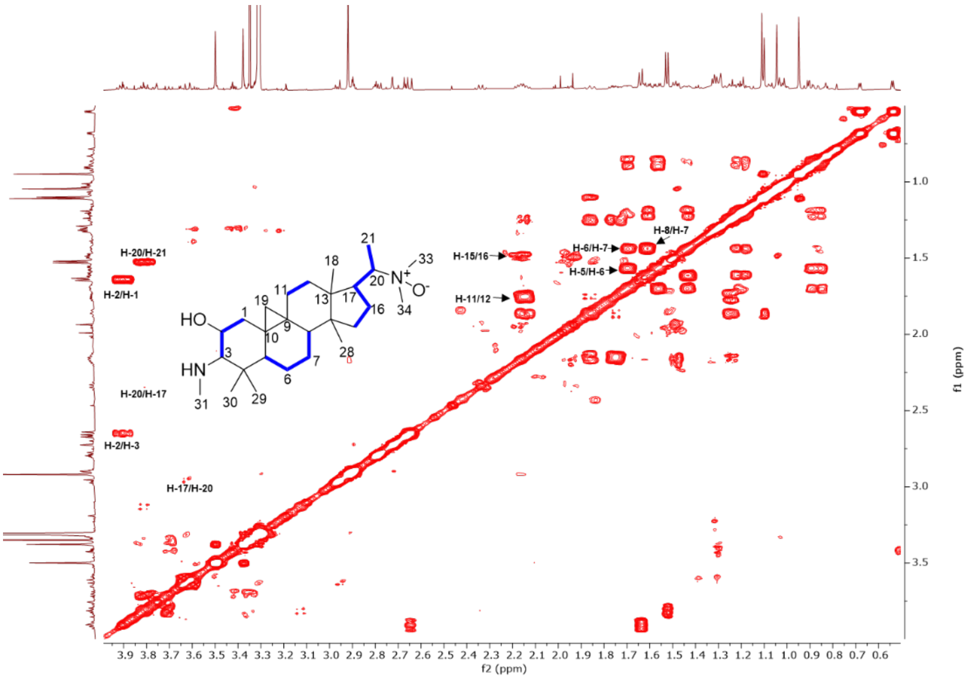

Figure S7. $^1H/^1H$ COSY spectrum (key correlations) of cycloprotobuxoline-C *N*20-oxide (**2**) (CD_3OD, 600 MHz).

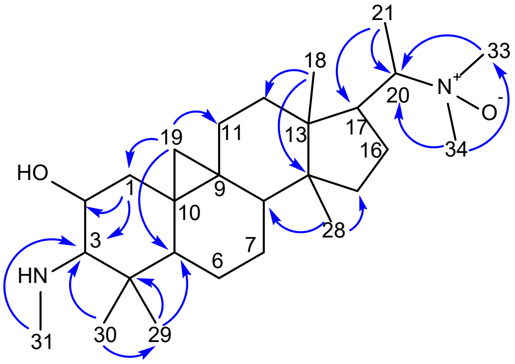

Figure S8. Key HMBC (blue arrows) correlations of cycloprotobuxoline-C *N*20-oxide (**2**).

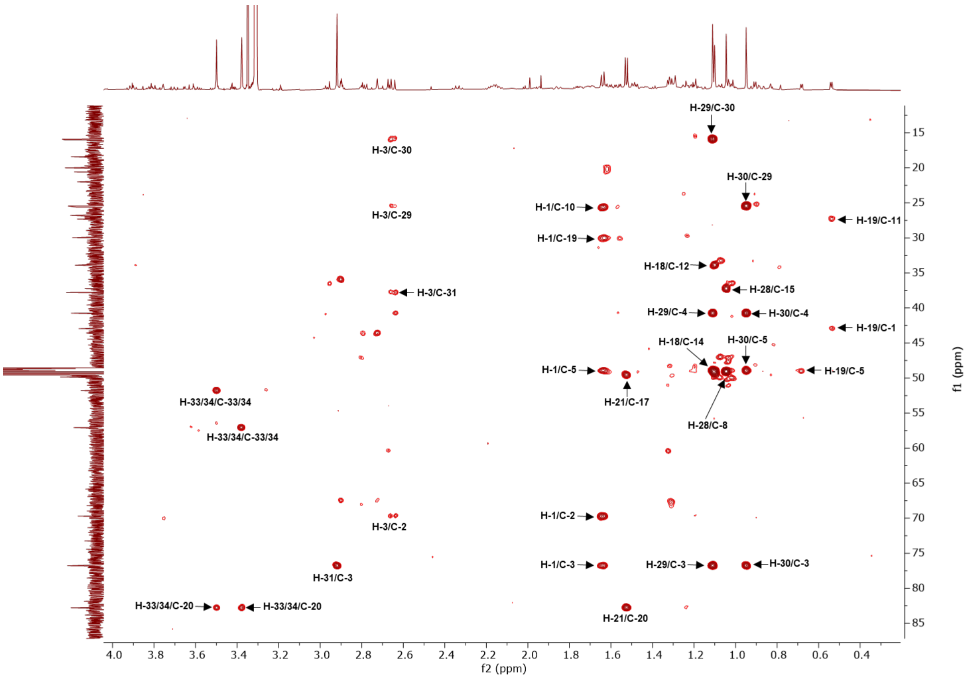

Figure S9. $^{1}H/^{13}C$ HMBC spectrum (key correlations) of cycloprotobuxoline-C *N*$_{20}$-oxide (**2**) (CD_3OD, 600/150 MHz).

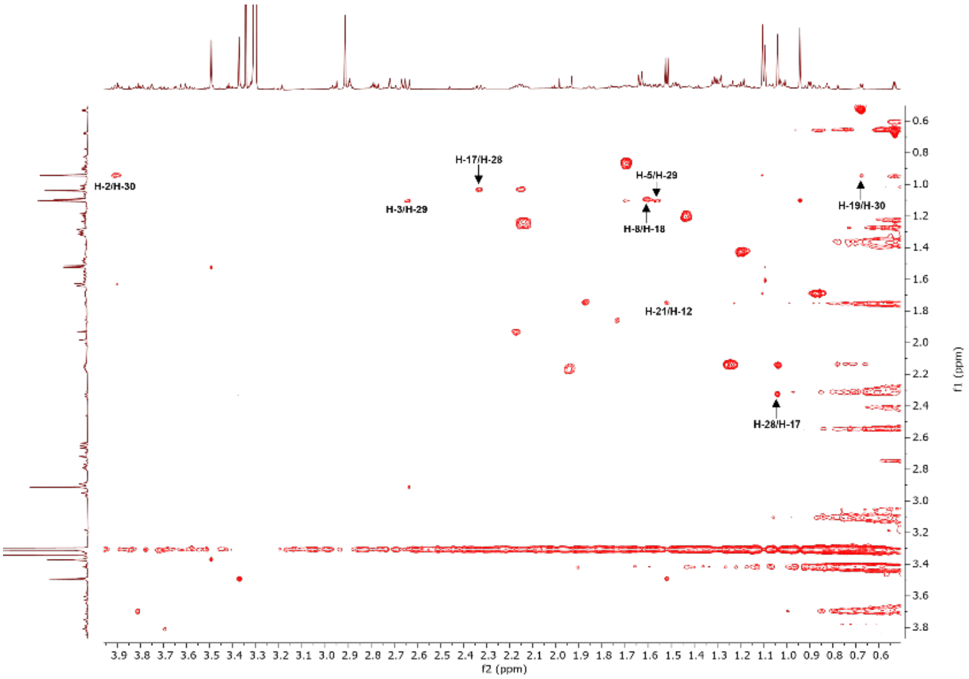

Figure S10. $^{1}H/^{1}H$ NOESY spectrum (key correlations) of cycloprotobuxoline-C *N*$_{20}$-oxide (**2**) (CD_3OD, 600 MHz).

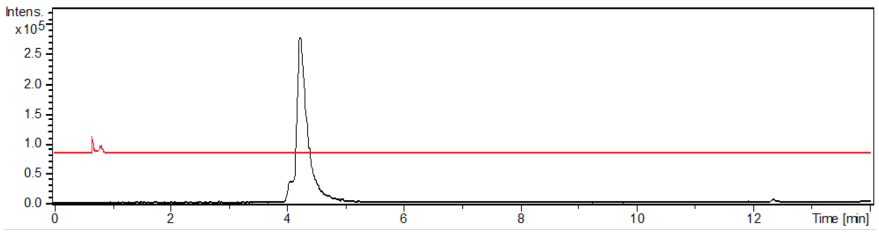

Figure S11. UHPLC/+ESI-QqTOF-MS/MS chromatogram of 16α-hydroxycycloprotobuxoline-C (**3**). Base peak chromatogram 200.0000–1000.0000 +All MS (black); UV chromatogram 200-400 nm (red).

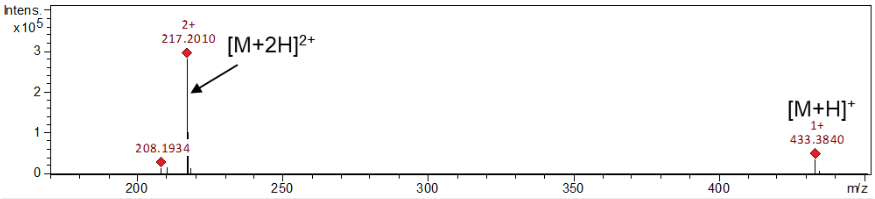

Figure S12. +ESI-QqTOF-MS spectrum of 16α-hydroxycycloprotobuxoline-C (**3**); *m/z* 433.3840 $[M+H]^+$, 217.2010 $[M+2H]^{2+}$

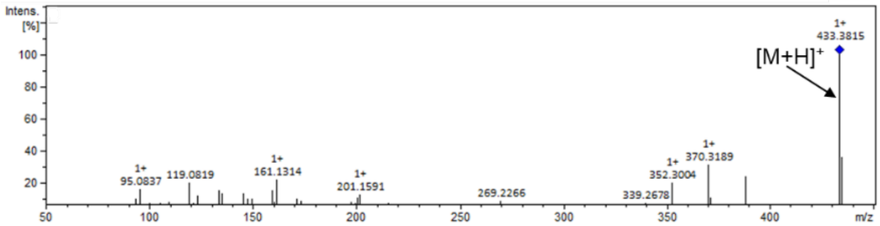

Figure S13. +ESI-QqTOF MS/MS spectrum of 16α-hydroxycycloprotobuxoline-C (**3**).

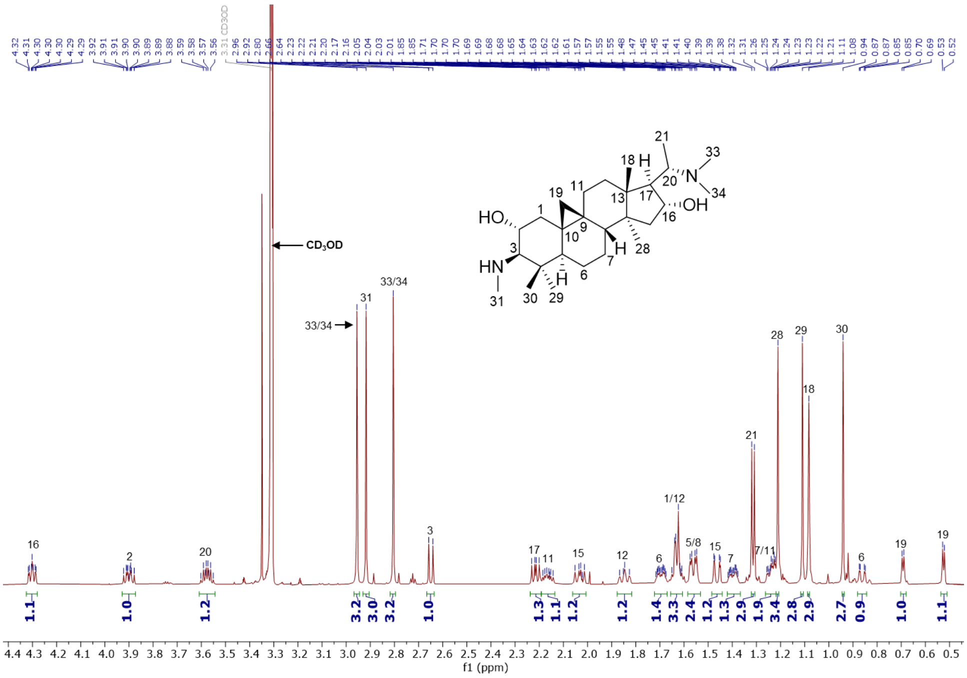

Figure S14. 1H NMR spectrum of 16α-hydroxycycloprotobuxoline-C (**3**) (CD_3OD, 600 MHz).

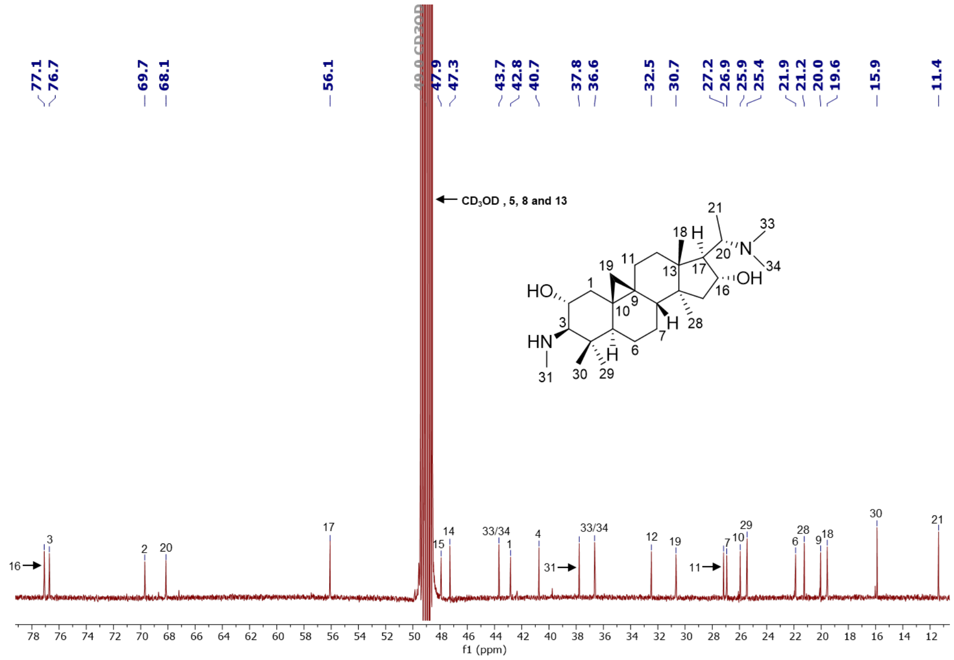

Figure S15. ^{13}C NMR spectrum of 16α-hydroxycycloprotobuxoline-C (**3**) (CD_3OD, 150 MHz).

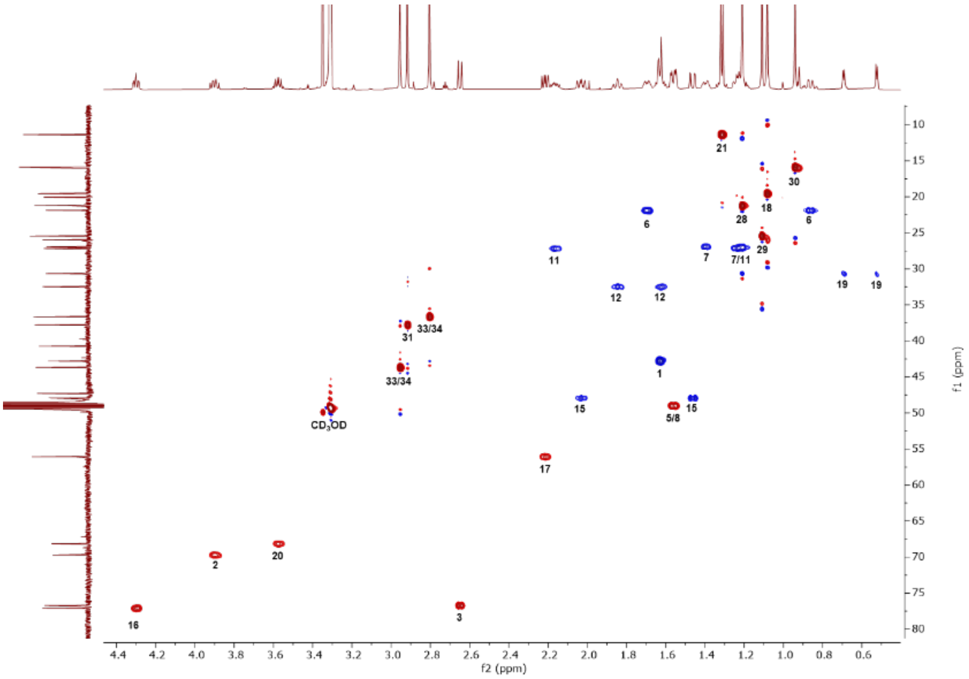

Figure S16. $^{1}H/^{13}C$ HSQC spectrum of 16α-hydroxycycloprotobuxoline-C (**3**) (CD_3OD, 600/150 MHz).

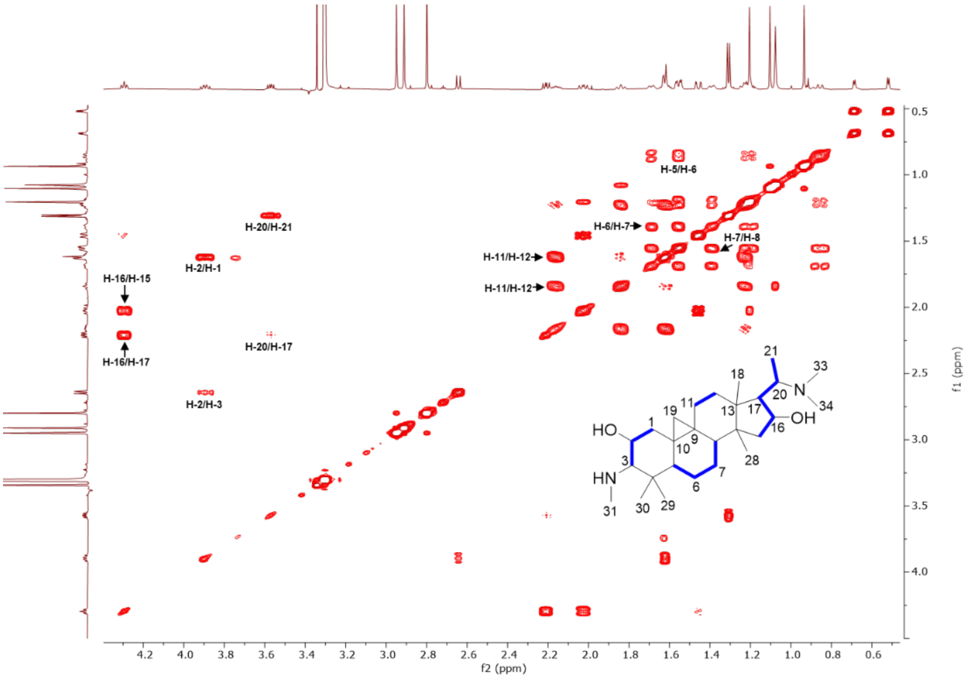

Figure S17. $^{1}H/^{1}H$ COSY spectrum (key correlations) of 16α-hydroxycycloprotobuxoline-C (**3**) (CD_3OD, 600 MHz).

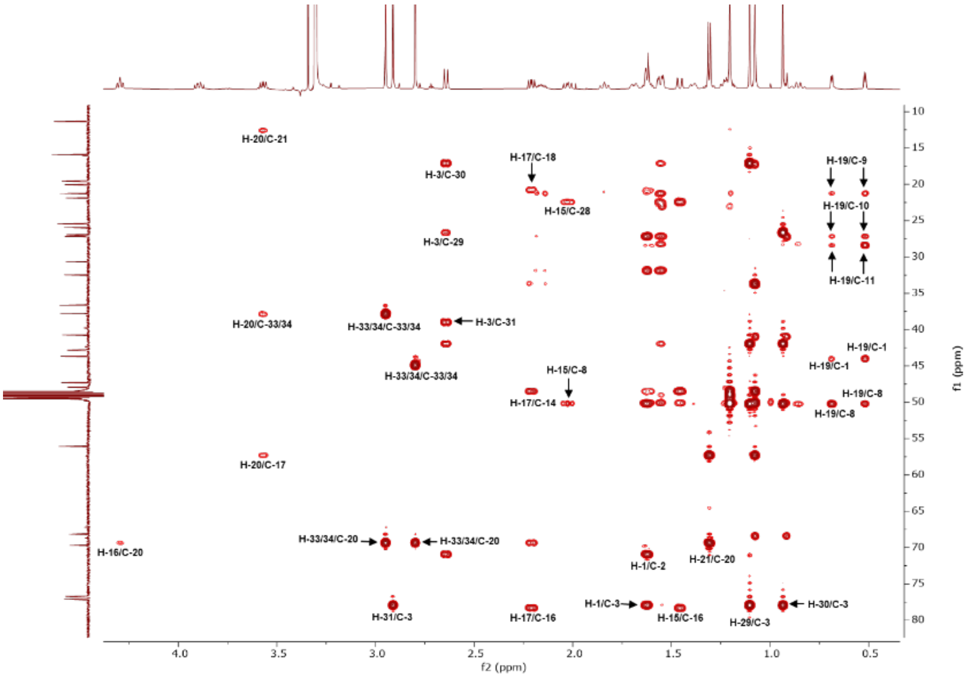

Figure S18. $^{1}H/^{13}C$ HMBC spectrum (key correlations) of 16α-hydroxycycloprotobuxoline-C (**3**) (CD_3OD, 600/150 MHz).

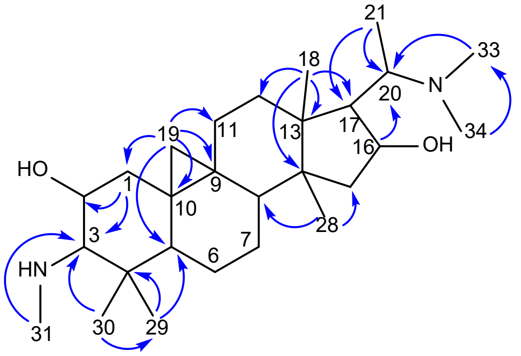

Figure S19. Key HMBC (blue arrows) correlations of 16α-hydroxycycloprotobuxoline-C (**3**).

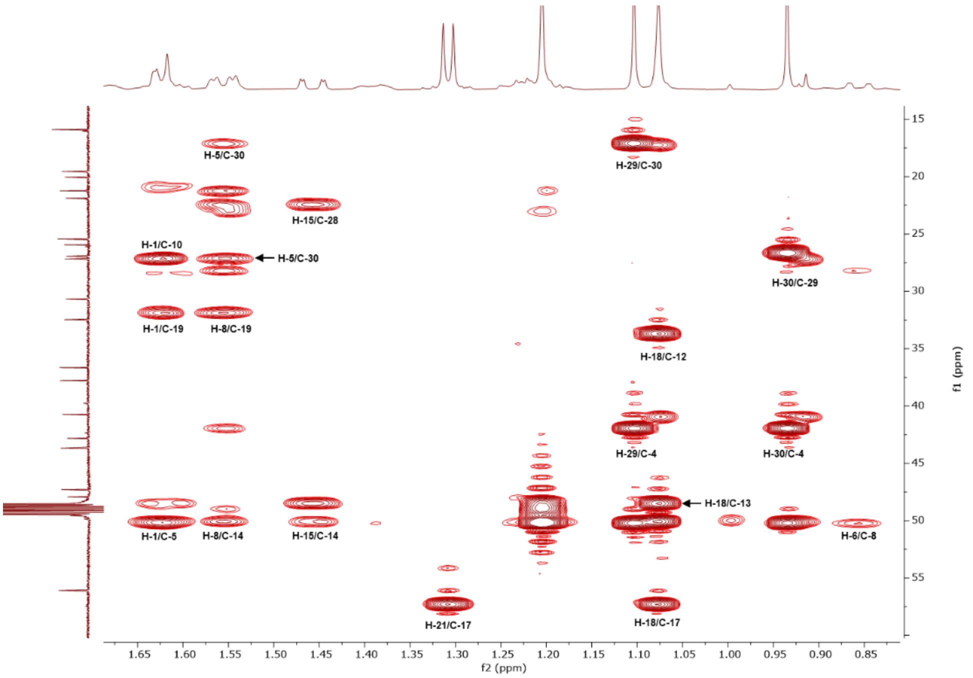

Figure S20. Detail of the $^{1}H/^{13}C$ HMBC spectrum (key correlations) of 16α-hydroxycycloprotobuxoline-C (**3**) (CD_3OD, 600/150 MHz).

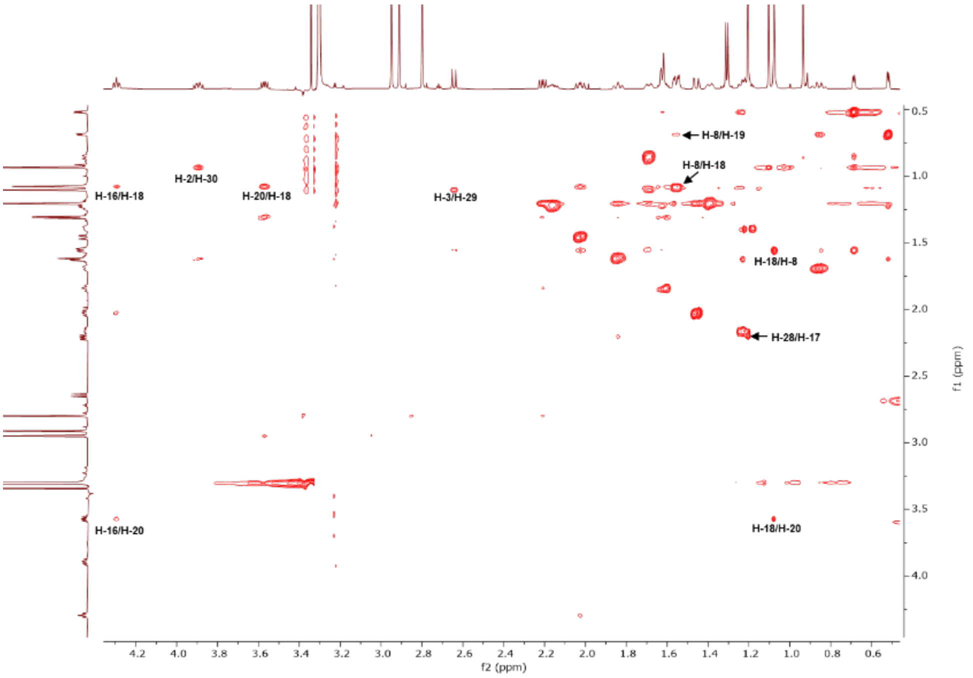

Figure S21. ^{1}H/^{1}H NOESY spectrum (key correlations) of 16α-hydroxycycloprotobuxoline-C (**3**) (CD_3OD, 600 MHz).

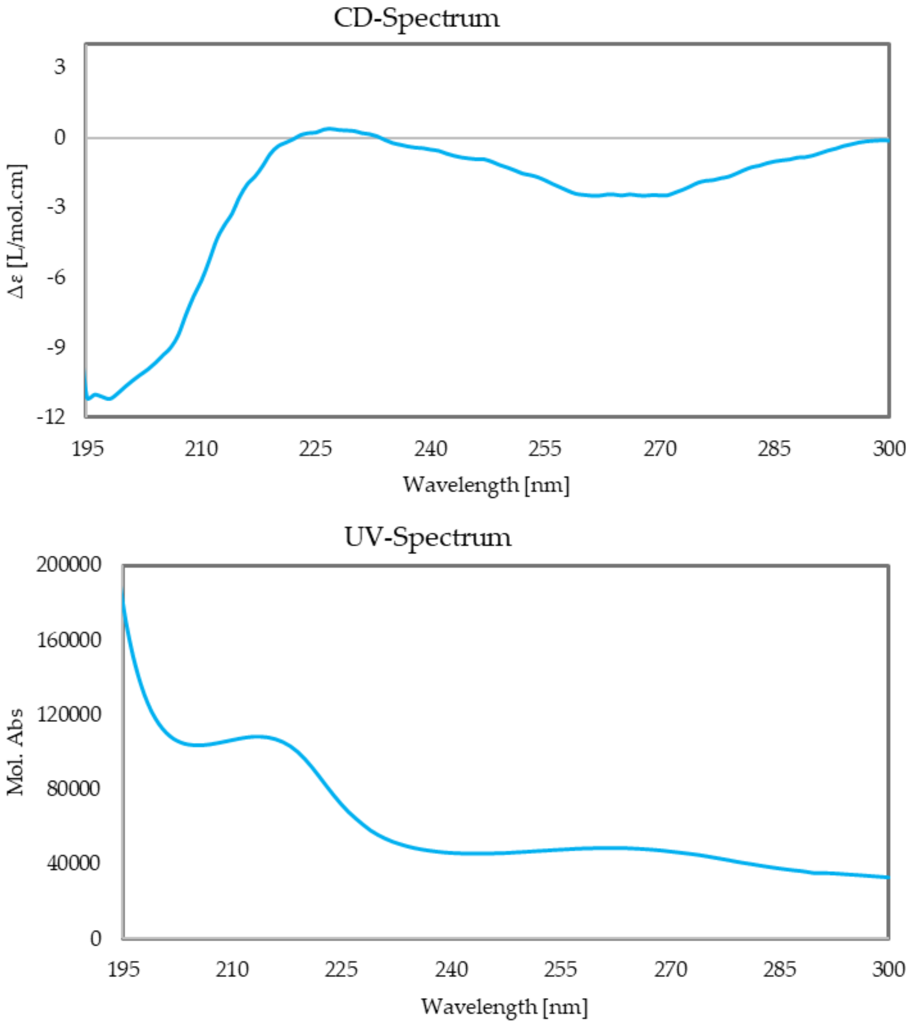

Figure S22. CD and UV spectra of 29-trimethoxybenzoyloxy cycloprotobuxoline-C (**5**) in MeOH (0.1 mg/mL). CD (λ[nm], Δε): 268, -2.47; 227, 0.39; 200, -10.7 (last reading); UV (λ[nm], log ε): 263, 4.69; 214, 5.04; 200, 5.06 (last reading).

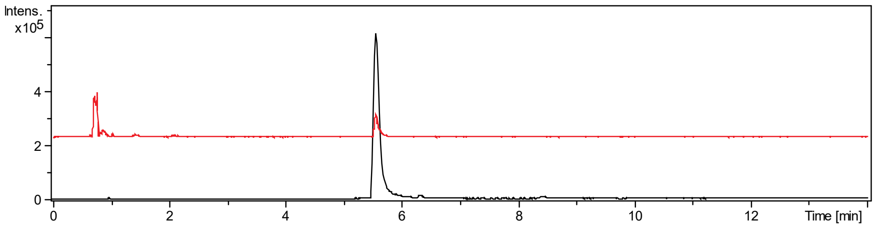

Figure S23. UHPLC/+ESI-QqTOF-MS/MS chromatogram of 29-trimethoxybenzoyloxy cycloprotobuxoline-C (**5**). Base peak chromatogram 200.0000–1000.0000 +All MS (black); UV chromatogram 200-400 nm (red).

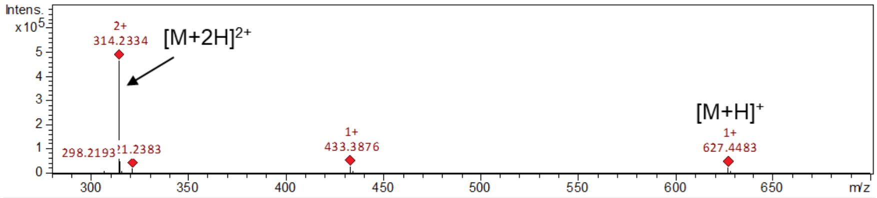

Figure S24. +ESI-QqTOF-MS spectrum of 29-trimethoxybenzoyloxy cycloprotobuxoline-C (**5**); *m/z* 627.4483 [M+H]$^+$, 314.2334[M+2H]$^{2+}$.

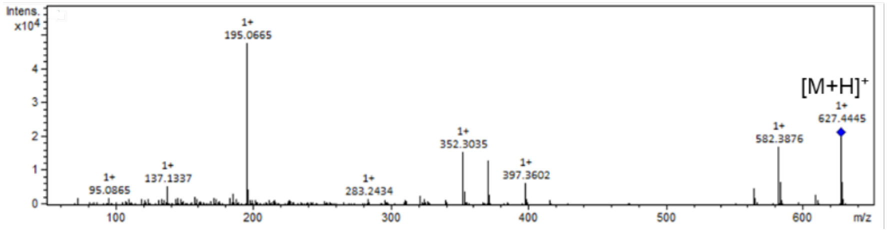

Figure S25. +ESI-QqTOF MS/MS spectrum of 29-trimethoxybenzoyloxy cycloprotobuxoline-C (**5**).

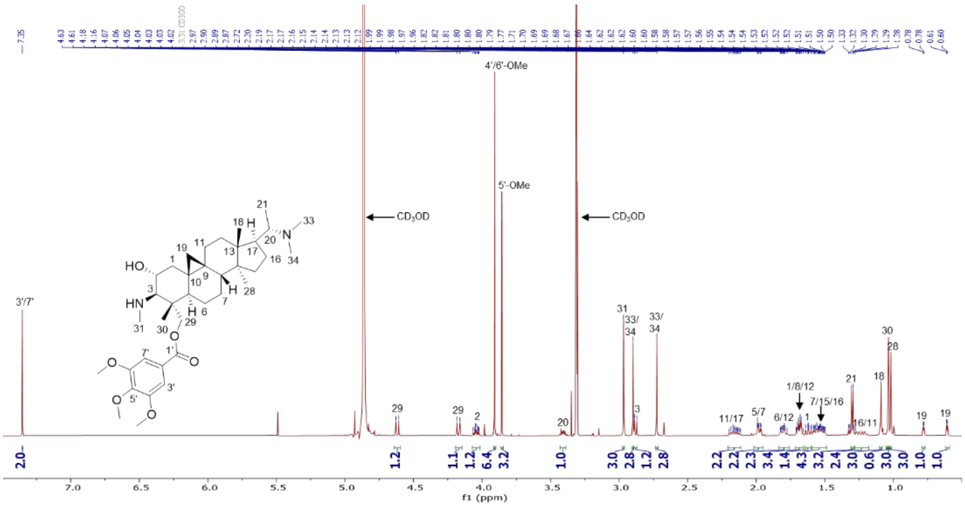

Figure S26. ^{1}H NMR spectrum of 29-trimethoxybenzoyloxy cycloprotobuxoline-C (**5**) (CD_3OD, 600 MHz).

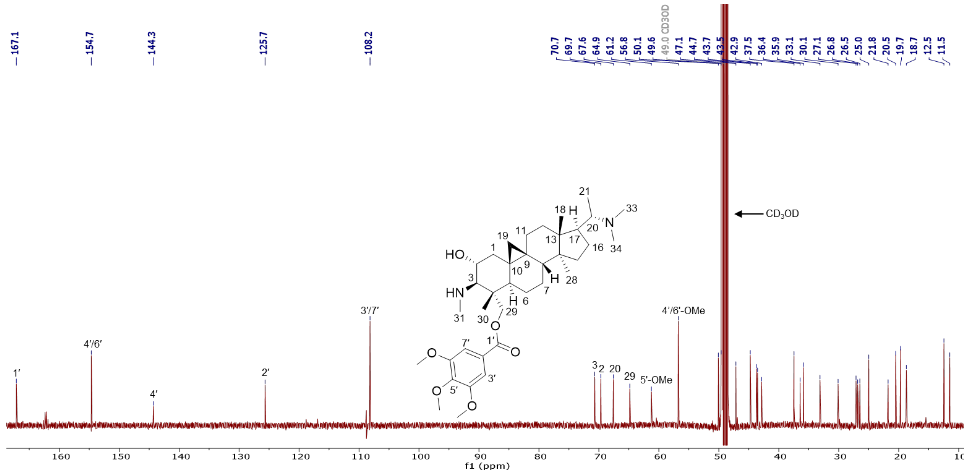

Figure S27. ^{13}C NMR spectrum of 29-trimethoxybenzoyloxy cycloprotobuxoline-C (**5**) (CD_3OD, 150 MHz).

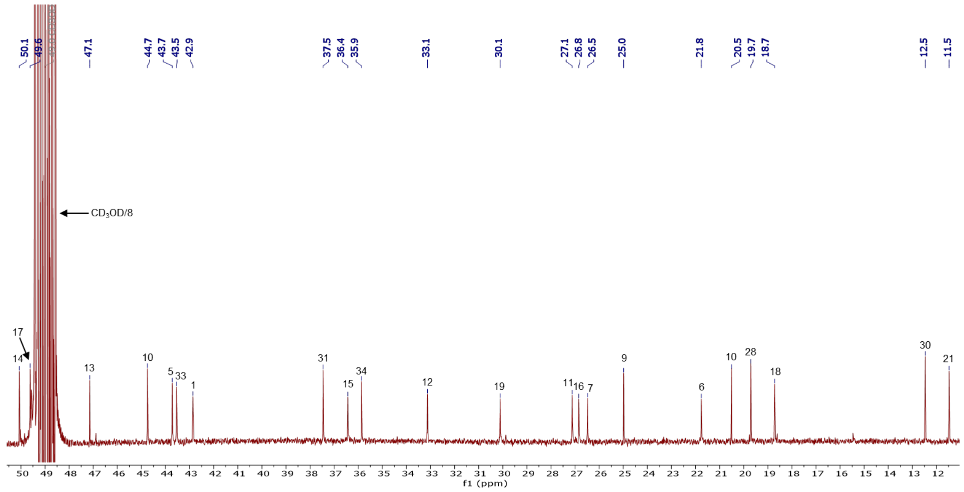

Figure S28. Detail of the ^{13}C NMR spectrum of 29-trimethoxybenzoyloxy cycloprotobuxoline-C (**5**) (CD_3OD, 150 MHz).

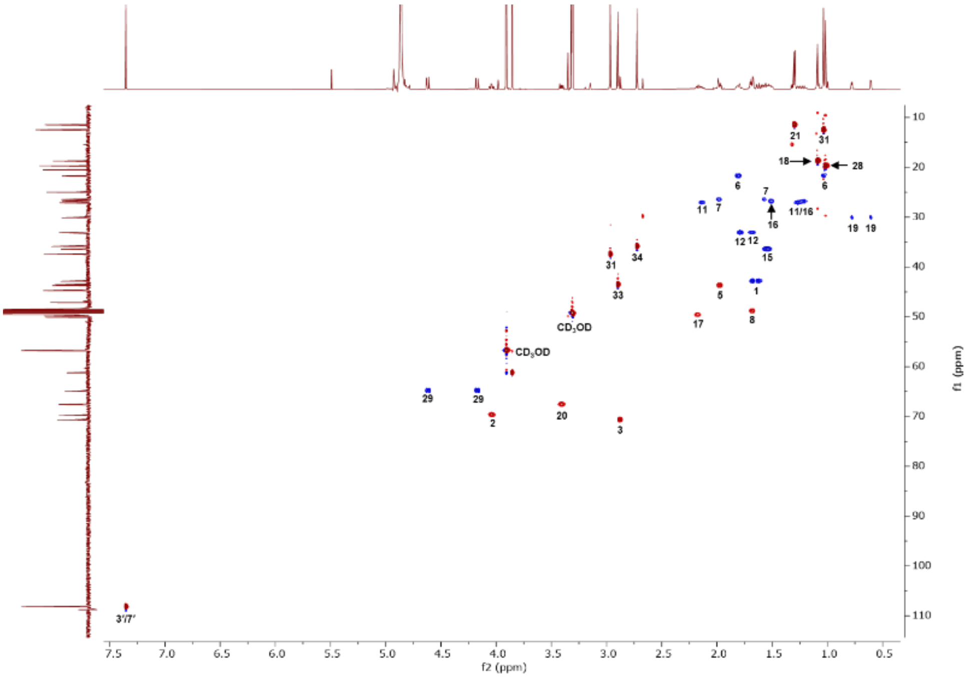

Figure S29. $^{1}H/^{13}C$ HSQC spectrum of 29-trimethoxybenzoyloxy cycloprotobuxoline-C (**5**) (CD_3OD, 600/150 MHz).

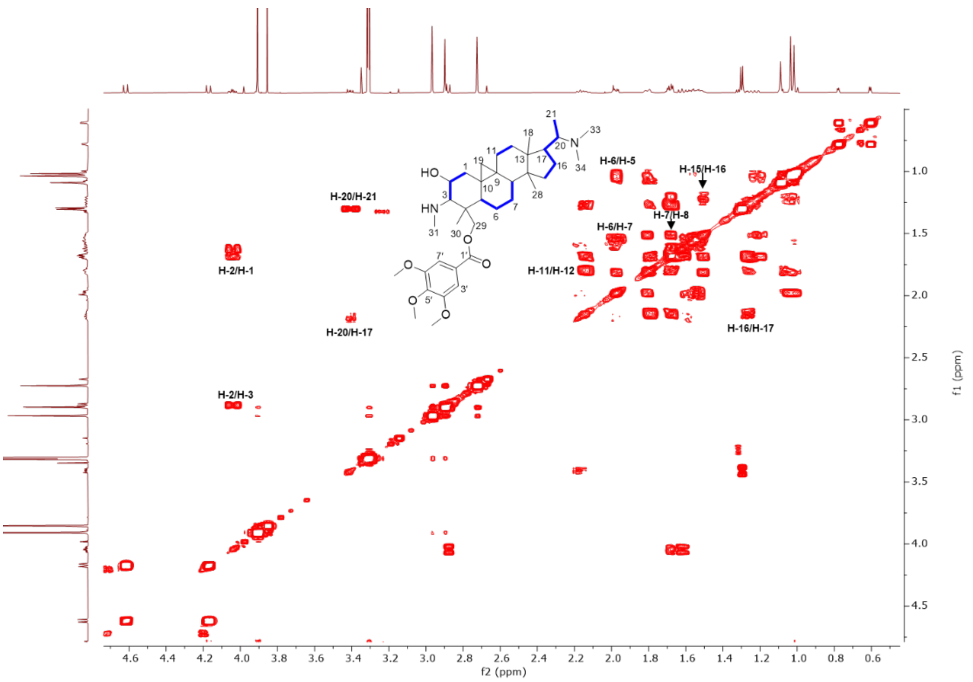

Figure S30. $^1H/^1H$ COSY spectrum (key correlations) of 29-trimethoxybenzoyloxy cycloprotobuxoline-C (**5**) (CD_3OD, 600 MHz).

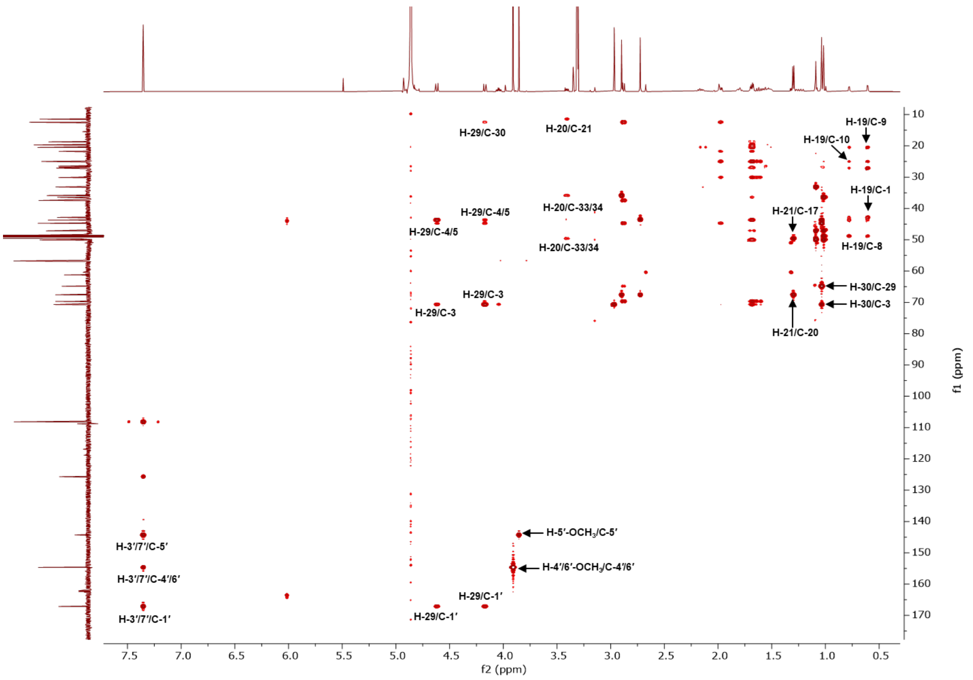

Figure S31. $^1H/^{13}C$ HMBC spectrum (key correlations) of 29-trimethoxybenzoyloxy cycloprotobuxoline-C (**5**) (CD_3OD, 600/150 MHz).

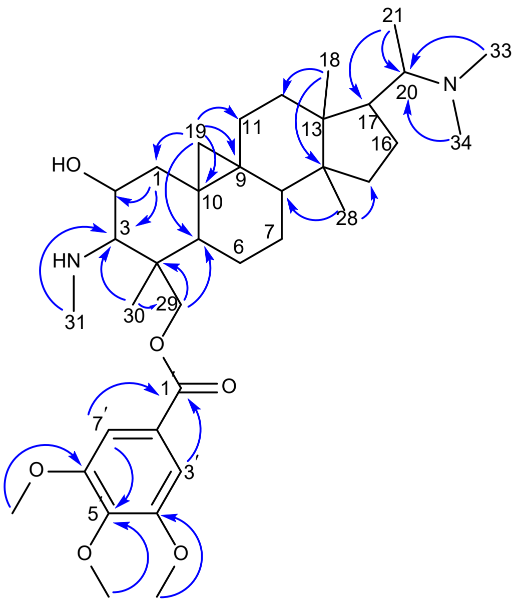

Figure S32. Key HMBC (blue arrows) correlations of 29-trimethoxybenzoyloxy cycloprotobuxoline-C (**5**).

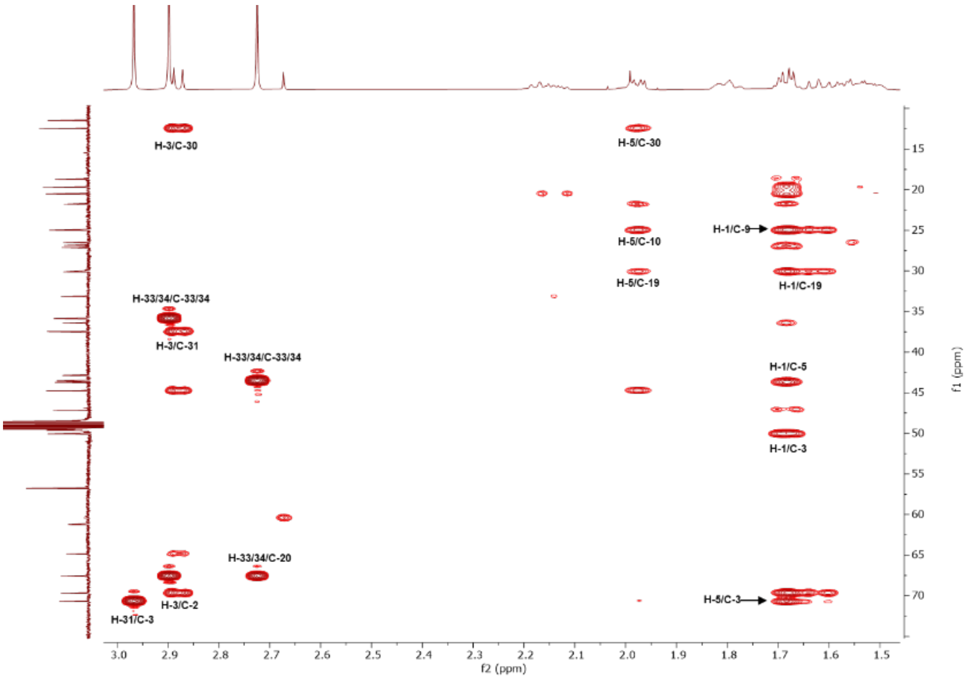

Figure S33. Detail of the $^{1}H/^{13}C$ HMBC spectrum (key correlations) of 29-trimethoxybenzoyloxy cycloprotobuxoline-C (**5**) (CD_3OD, 600/150 MHz).

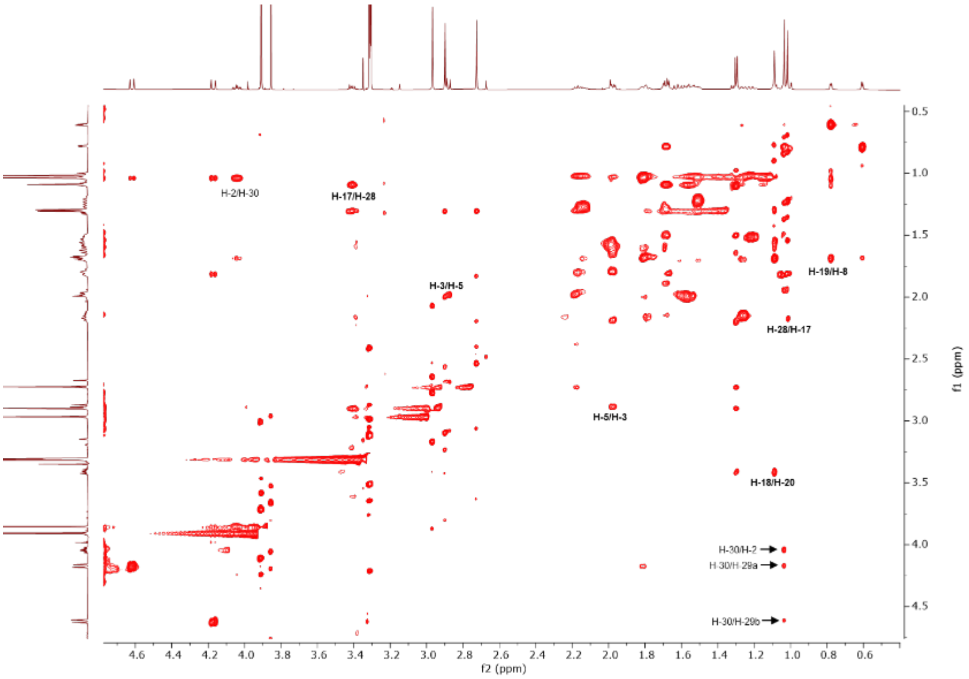

Figure S34. ^{1}H/^{1}H NOESY spectrum (key correlations) of 29-trimethoxybenzoyloxy cycloprotobuxoline-C (**5**) (CD_3OD, 600 MHz).

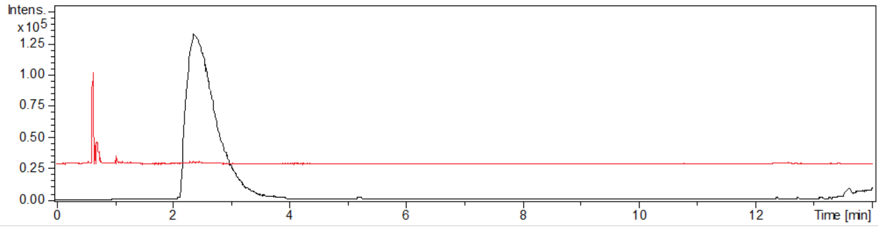

Figure S35. UHPLC/+ESI-QqTOF-MS/MS chromatogram of *N*3-demethylcycloprotobuxoline-C (**6**). Base peak chromatogram 200.0000–1000.0000 +All MS (black); UV chromatogram 200-400 nm (red).

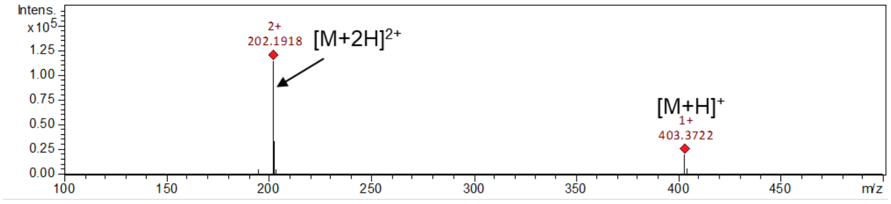

Figure S36. +ESI-QqTOF-MS spectrum of *N*3-demethylcycloprotobuxoline-C (**6**); *m/z* 403.3722 [M+H]+, 202.1918[M+2H]2+.

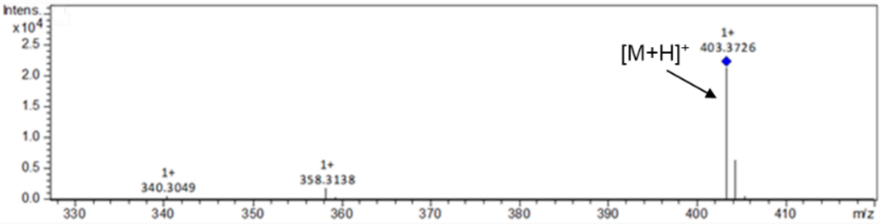

Figure S37. +ESI-QqTOF MS/MS spectrum of *N*3-demethylcycloprotobuxoline-C (**6**).

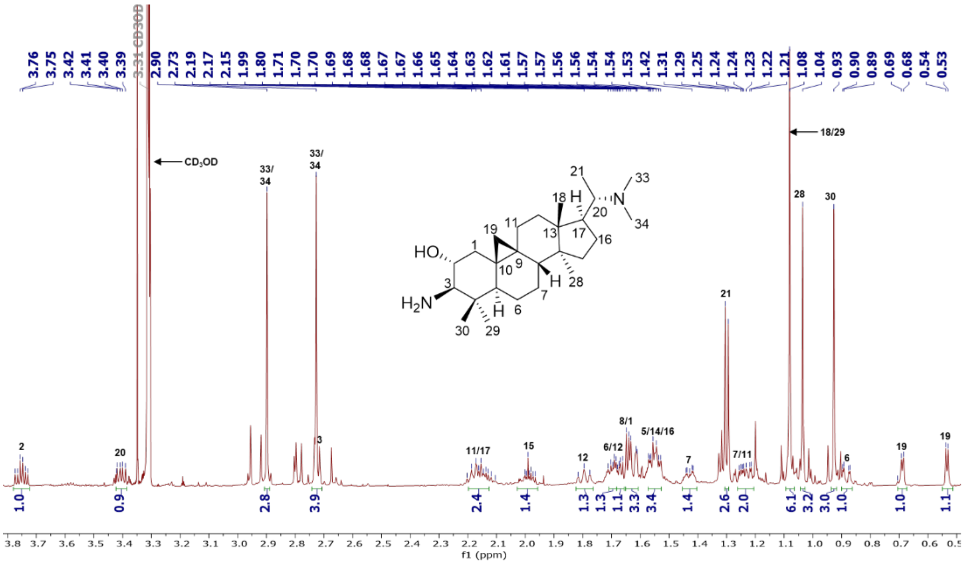

Figure S38. ^{1}H NMR spectrum of *N*3-demethylcycloprotobuxoline-C (**6**) (CD_3OD, 600 MHz).

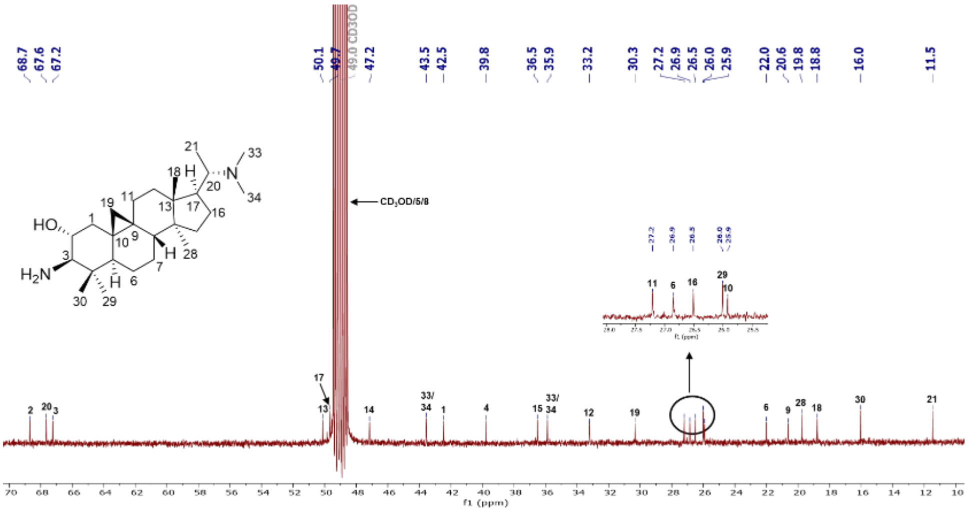

Figure S39. ^{13}C NMR spectrum of *N*3-demethylcycloprotobuxoline-C (**6**) (CD_3OD, 150 MHz).

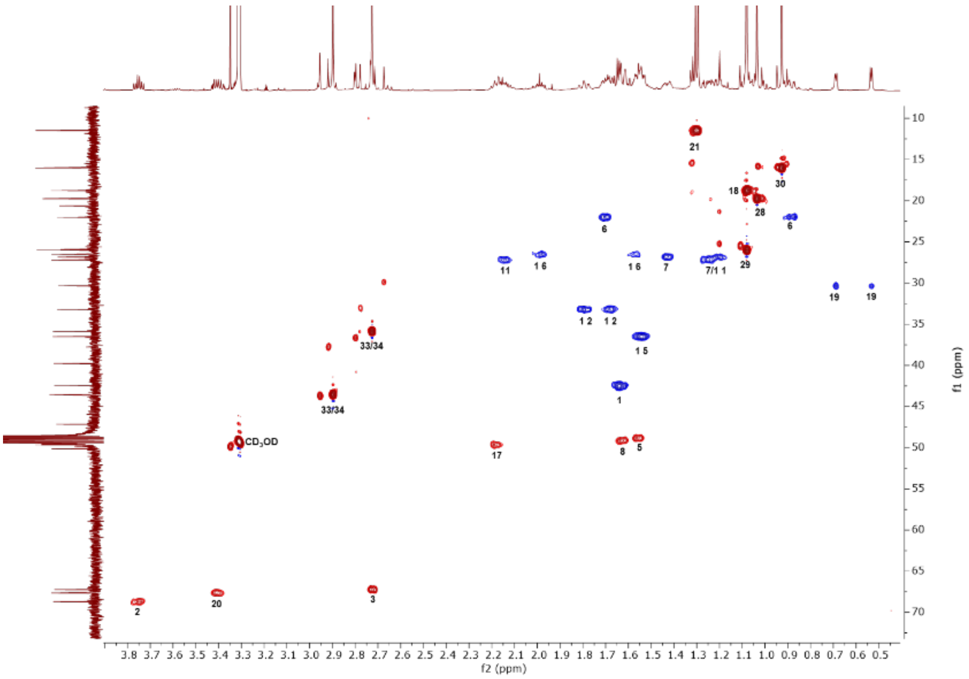

Figure S40. $^{1}H/^{13}C$ HSQC spectrum of *N*3-demethylcycloprotobuxoline-C (**6**) (CD_3OD, 600/150 MHz).

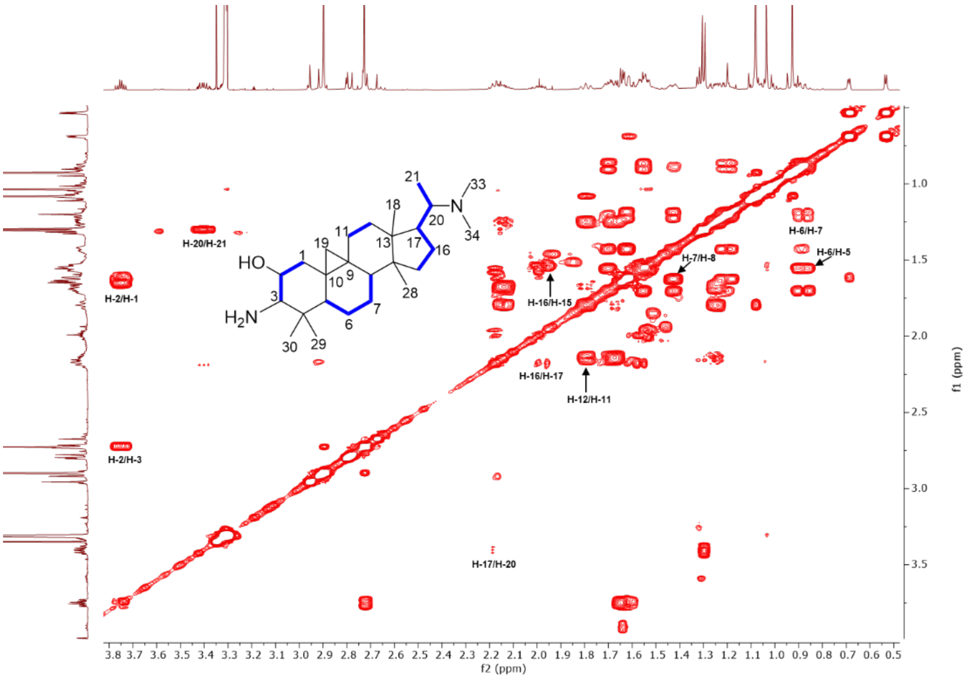

Figure S41. ^{1}H/^{1}H COSY spectrum (key correlations) of *N*3-demethylcycloprotobuxoline-C (**6**) (CD_3OD, 600 MHz).

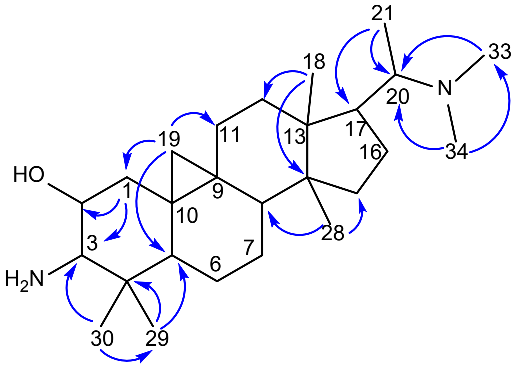

Figure S42. Key HMBC (blue arrows) correlations of *N*3-demethylcycloprotobuxoline-C (**6**).

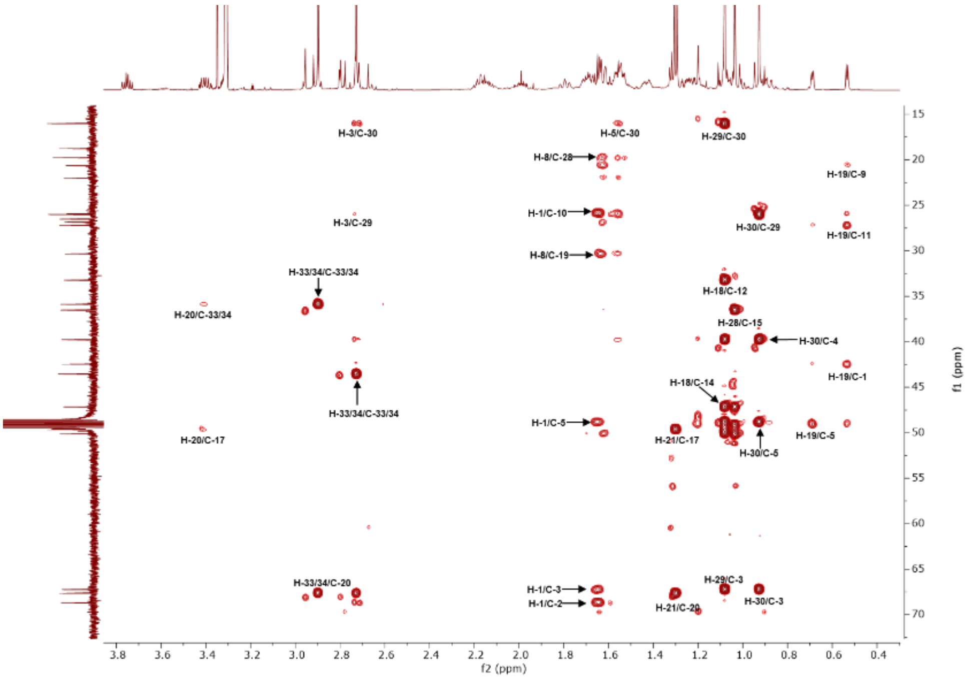

Figure S43. $^{1}H/^{13}C$ HMBC spectrum (key correlations) of *N*3-demethylcycloprotobuxoline-C (**6**) (CD_3OD, 600/150 MHz).

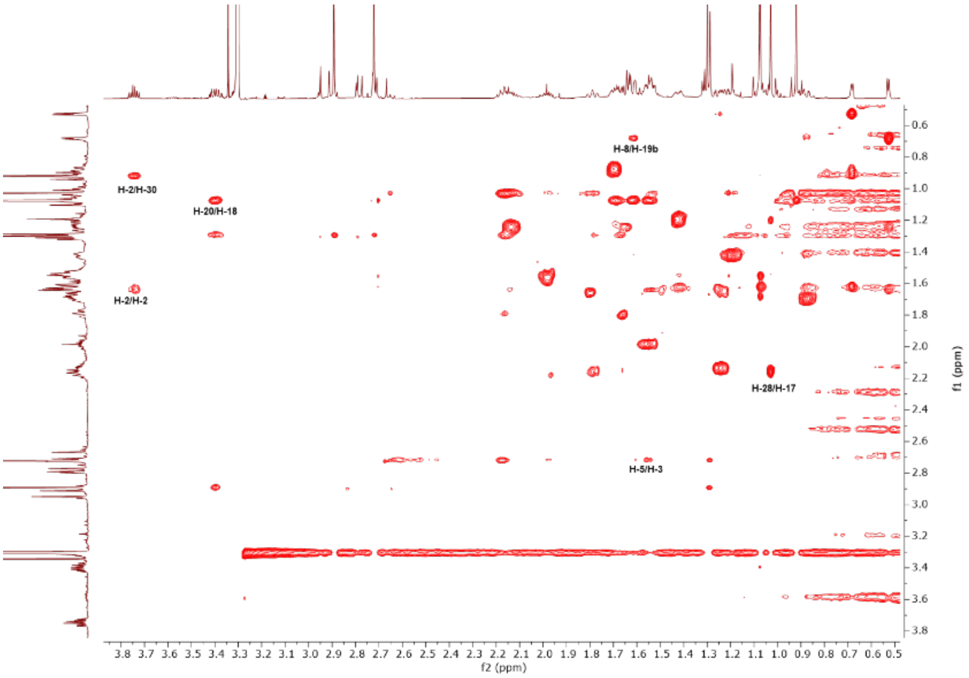

Figure S44. $^{1}H/^{1}H$ NOESY spectrum (key correlations) of *N*3-demethylcycloprotobuxoline-C (**6**) (CD_3OD, 600 MHz).

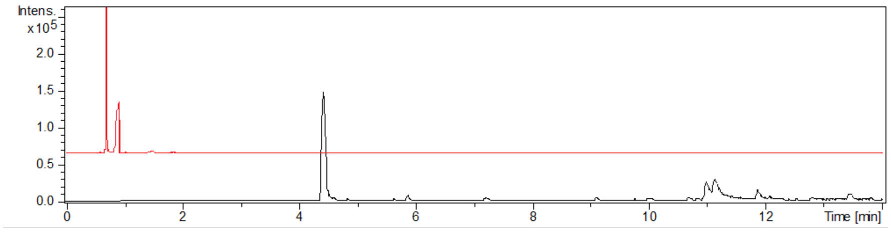

Figure S45. UHPLC/+ESI-QqTOF-MS/MS chromatogram of 16*α* hydroxy-*N*3-demethylcycloprotobuxoline-C (**7**). Base peak chromatogram 200.0000–1000.0000 +All MS (black); UV chromatogram 200-400 nm (red).]

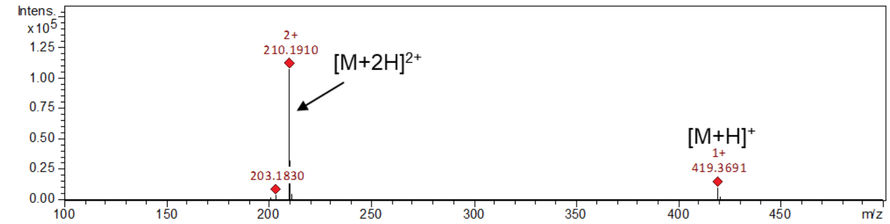

Figure S46. +ESI-QqTOF-MS spectrum of 16*α* hydroxy-*N*3-demethylcycloprotobuxoline-C (**7**); *m/z* 419.3691 $[M+H]^+$, 210.1910 $[M+2H]^{2+}$.

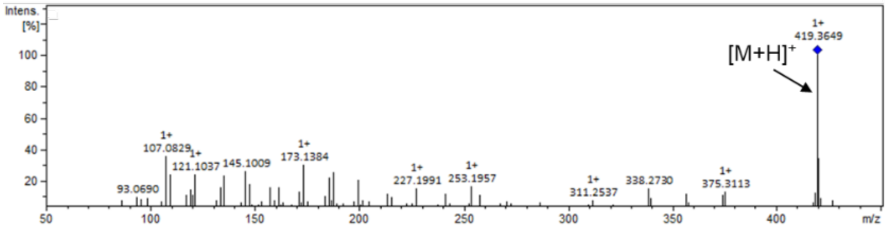

Figure S47. +ESI-QqTOF MS/MS spectrum of 16*α* hydroxy-*N*3-demethylcycloprotobuxoline-C (**7**).

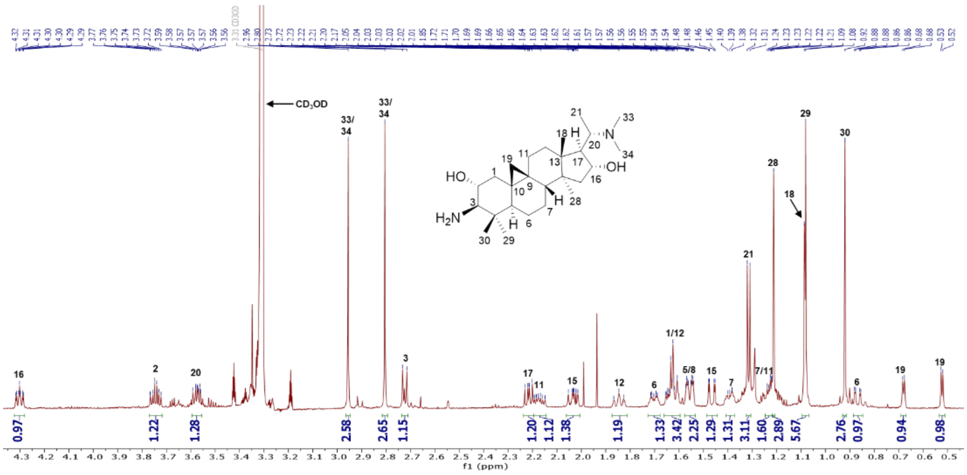

Figure S48. ^{1}H NMR spectrum of 16*α* hydroxy-*N*3-demethylcycloprotobuxoline-C (**7**) (CD_3OD, 600 MHz).

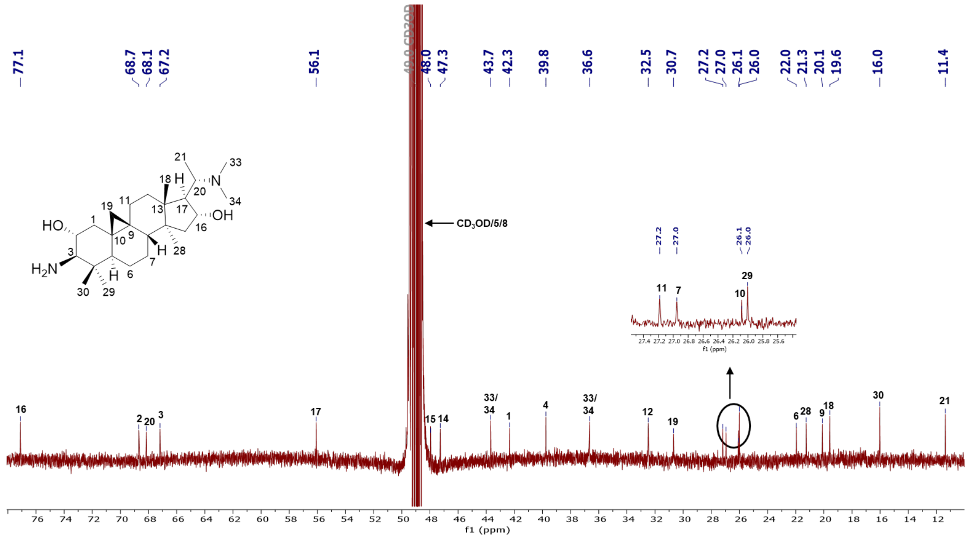

Figure S49. ^{13}C NMR spectrum of 16*α* hydroxy-*N*3-demethylcycloprotobuxoline-C (**7**) (CD_3OD, 150 MHz).

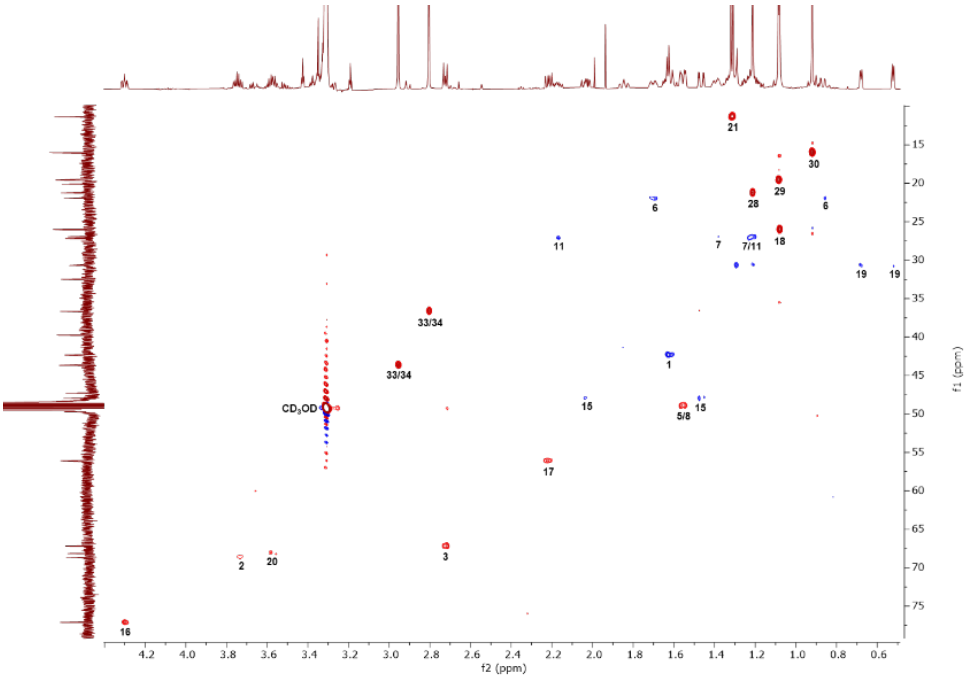

Figure S50. $^{1}H/^{13}C$ HSQC spectrum of 16*α* hydroxy-*N*3-demethylcycloprotobuxoline-C (**7**) (CD_3OD, 600/150 MHz).

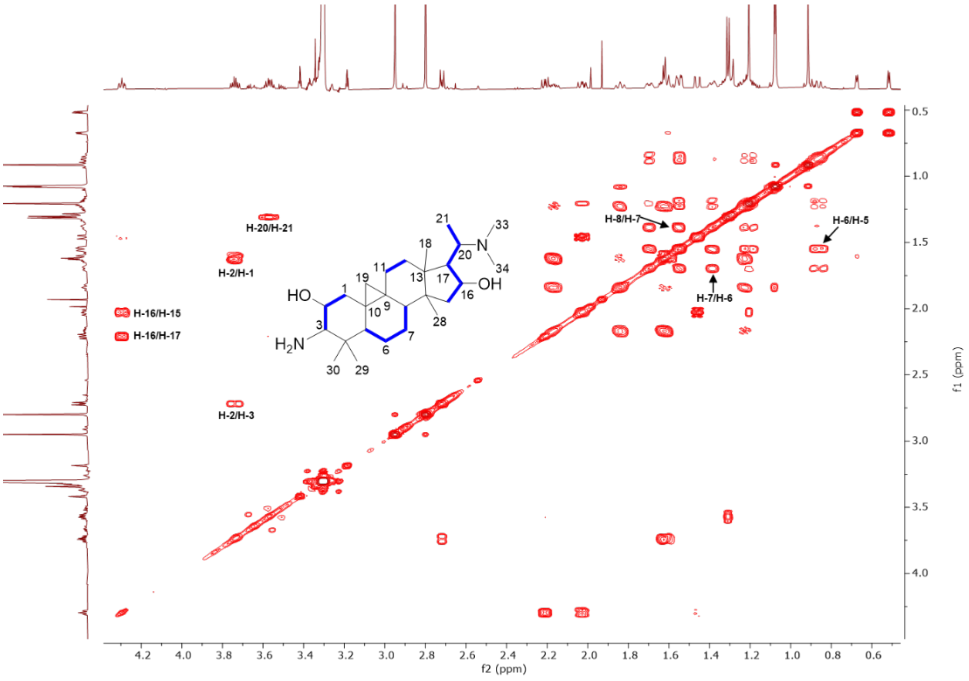

Figure 51. $^{1}H/^{1}H$ COSY spectrum (key correlations) of 16*α* hydroxy-*N*3-demethylcycloprotobuxoline-C (**7**) (CD_3OD, 600 MHz).

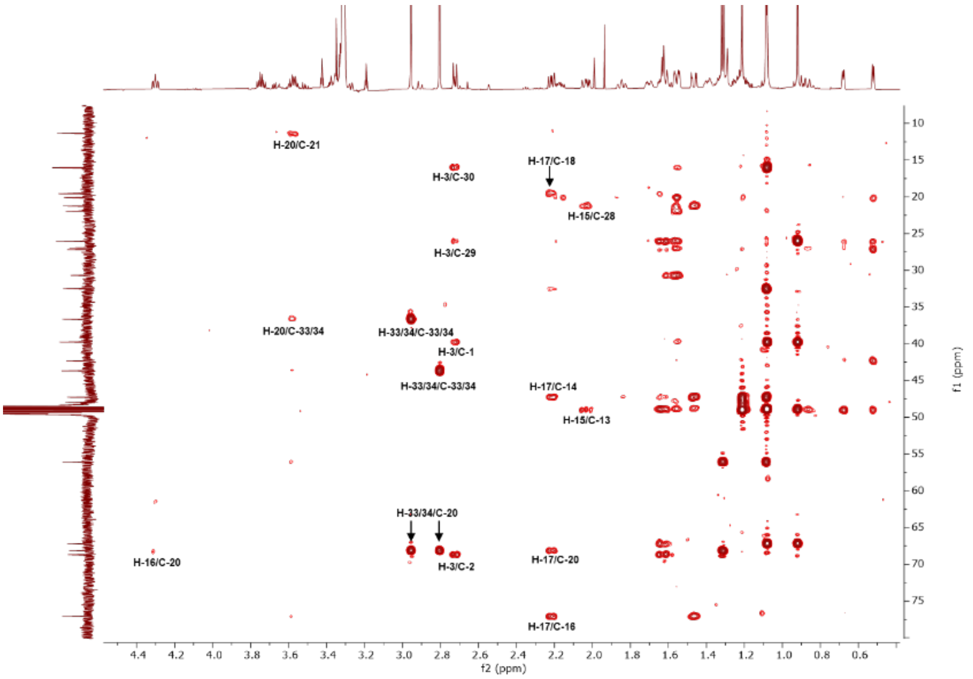

Figure S52. ^{1}H/^{13}C HMBC spectrum (key correlations) of 16*α* hydroxy-N_3-demethylcycloprotobuxoline-C (**7**) (CD_3OD, 600/150 MHz).

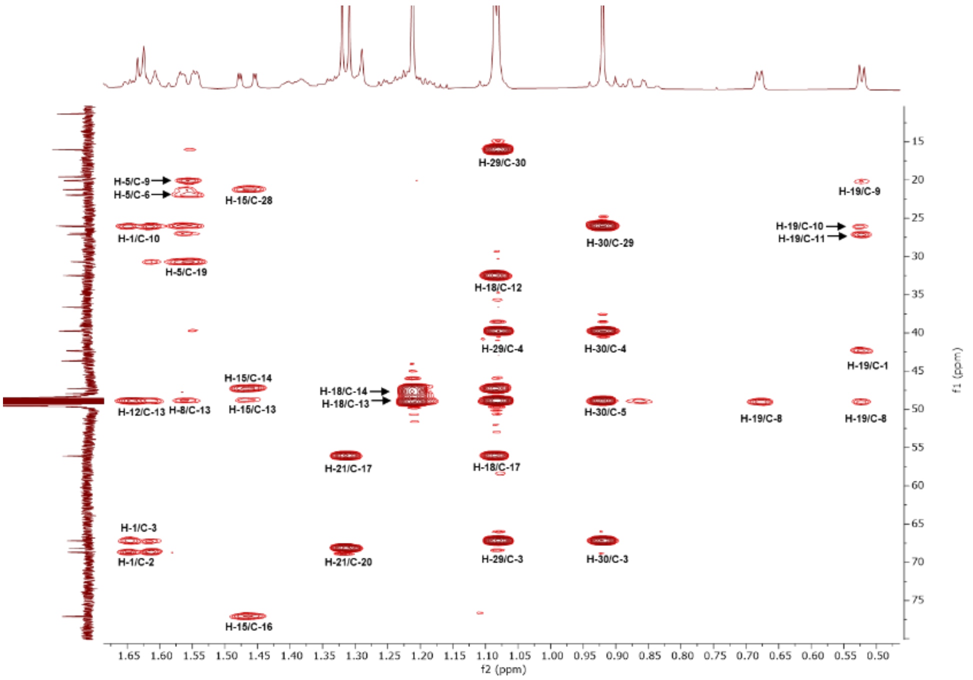

Figure S53. Detail of the ^{1}H/^{13}C HMBC spectrum (key correlations) of 16*α* hydroxy-N_3-demethylcycloprotobuxoline-C (**7**) (CD_3OD, 600/150 MHz).

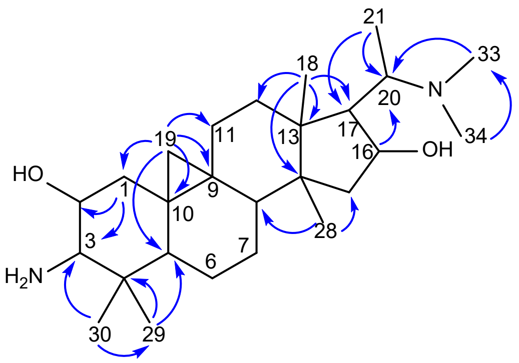

Figure 54. Key HMBC (blue arrows) correlations of 16*α* hydroxy-*N*3-demethylcycloprotobuxoline-C (**7**).

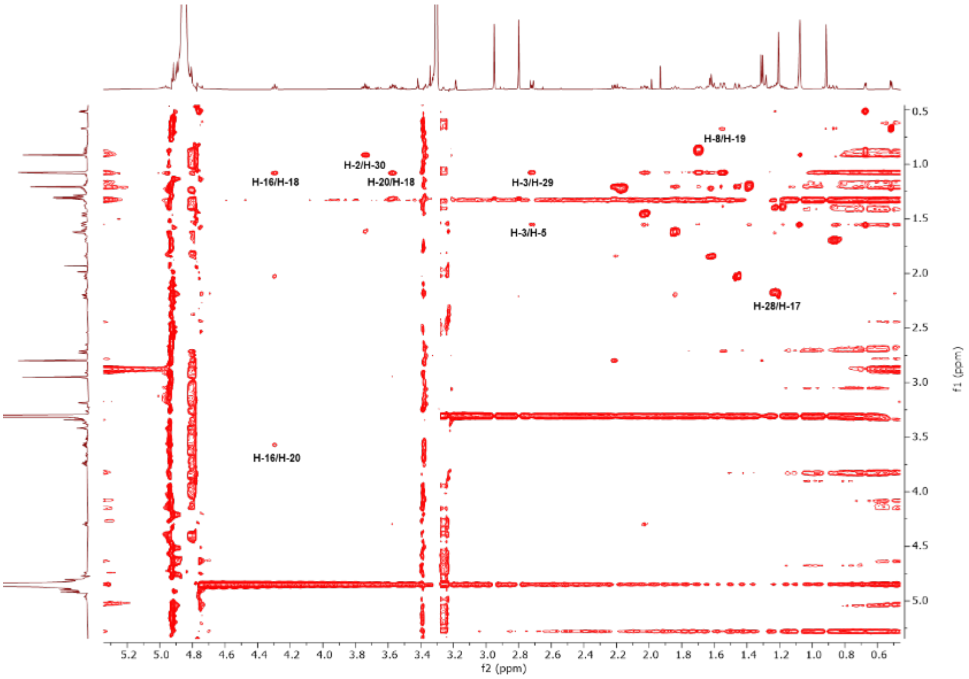

Figure S55. $^{1}H/^{1}H$ NOESY spectrum (key correlations) of 16*α* hydroxy-*N*3-demethylcycloprotobuxoline-C (**7**) (CD_3OD, 600 MHz).

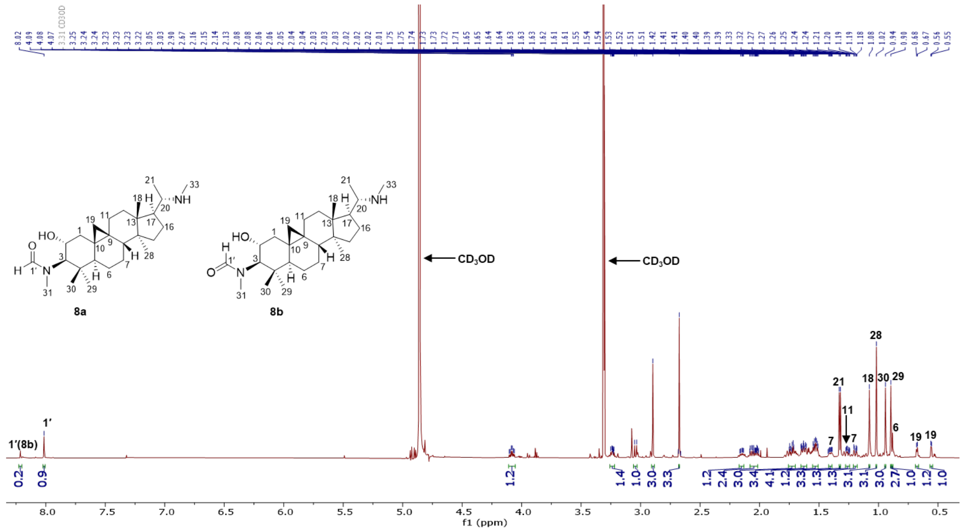

Figure S56. ^{1}H NMR spectrum of cycloprotobuxoline-D *N*3-*trans*-formamide (**8a**) and cycloprotobuxoline-D *N*3-*cis*-formamide (**8b**) (CD_3OD, 600 MHz).

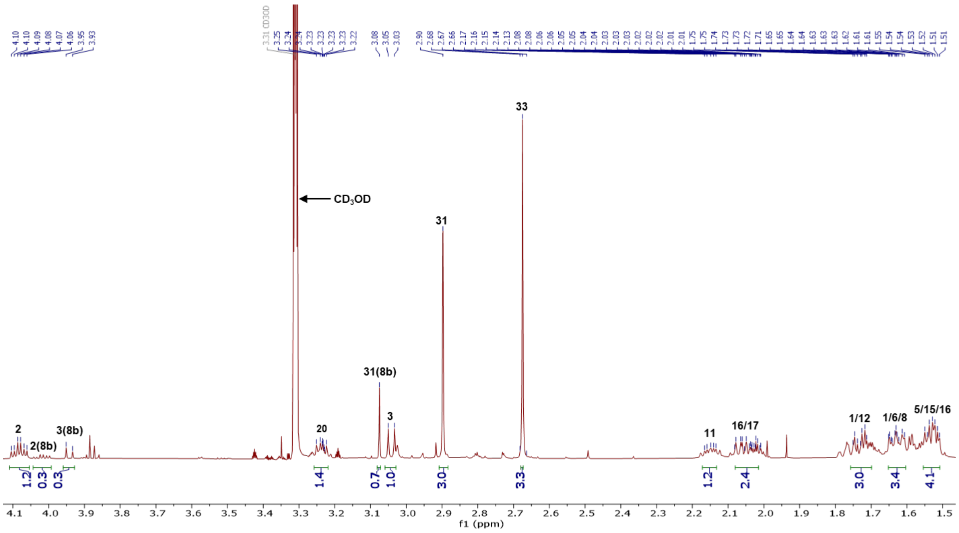

Figure S57. Detail of the ^{1}H NMR spectrum of cycloprotobuxoline-D *N*3-*trans*-formamide (**8a**) and cycloprotobuxoline-D *N*3-*cis*-formamide (**8b**) (CD_3OD, 600 MHz).

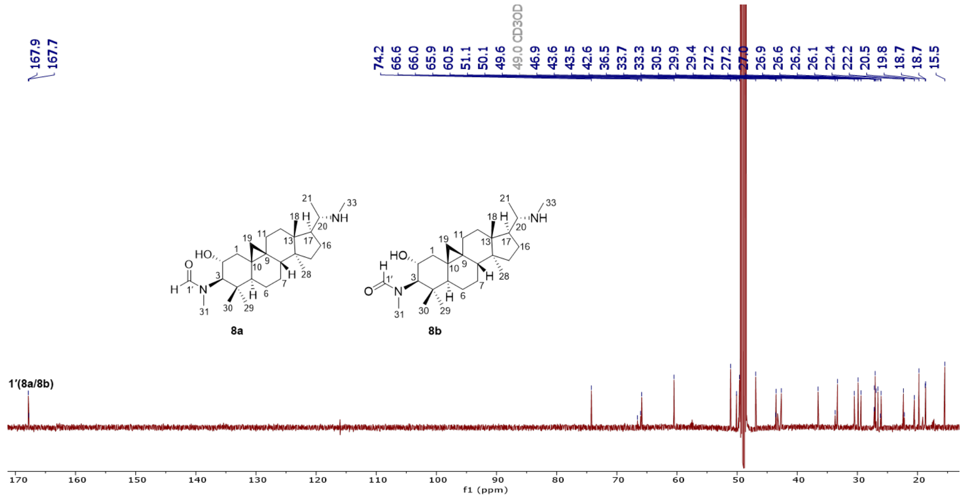

Figure S58. ^{13}C NMR spectrum of cycloprotobuxoline-D *N*3-*trans*-formamide (**8a**) and cycloprotobuxoline-D *N*3-*cis*-formamide (**8b**) (CD_3OD, 150 MHz).

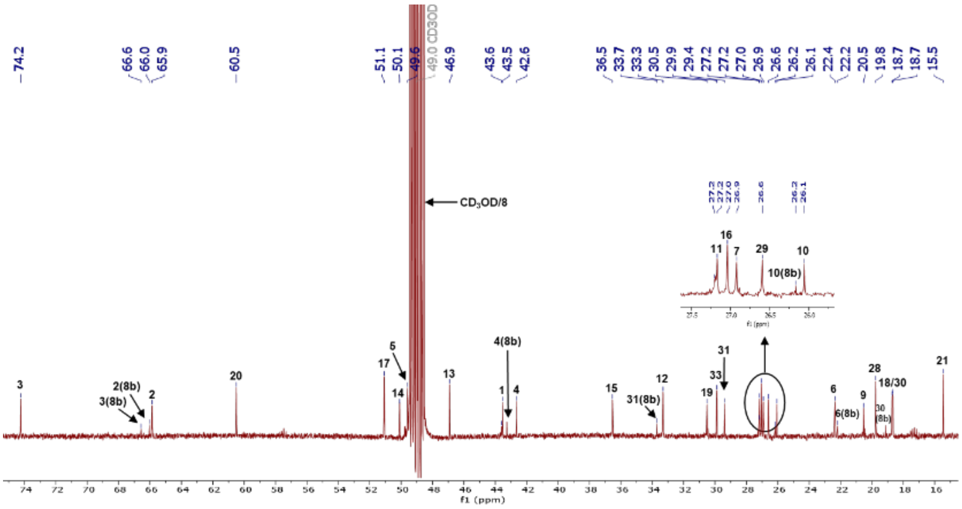

Figure S59. Detail of the ^{13}C NMR spectrum of cycloprotobuxoline-D *N*3-*trans*-formamide (**8a**) and cycloprotobuxoline-D *N*3-*cis*-formamide (**8b**) (CD_3OD, 150 MHz).

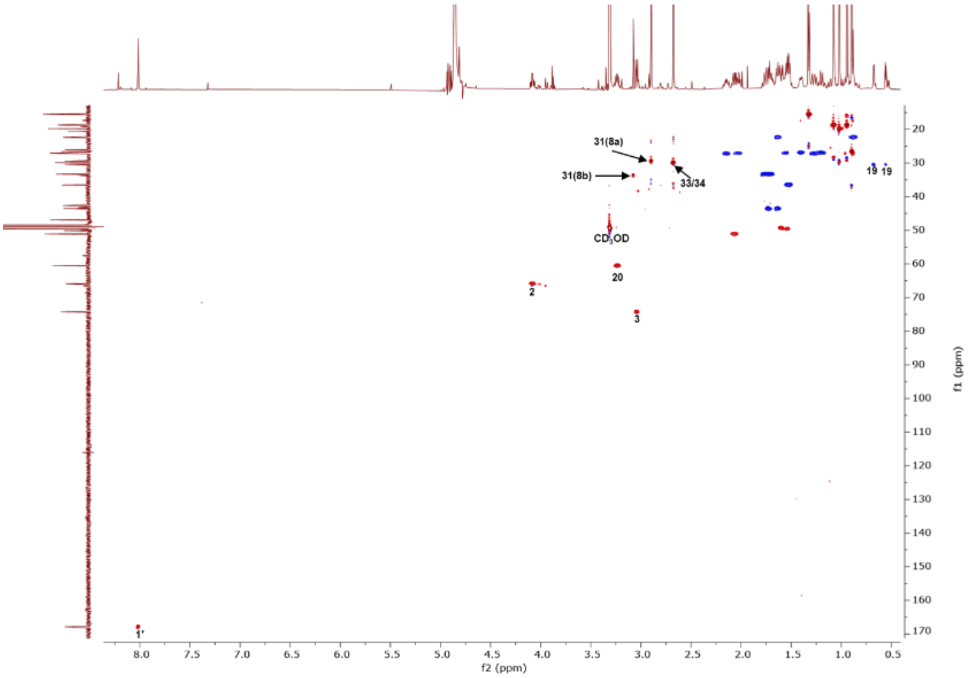

Figure S60. ^{1}H/^{13}C HSQC spectrum of cycloprotobuxoline-D *N*3-*trans*-formamide (**8a**) and cycloprotobuxoline-D *N*3-*cis*-formamide (**8b**) (CD_3OD, 600/150 MHz).

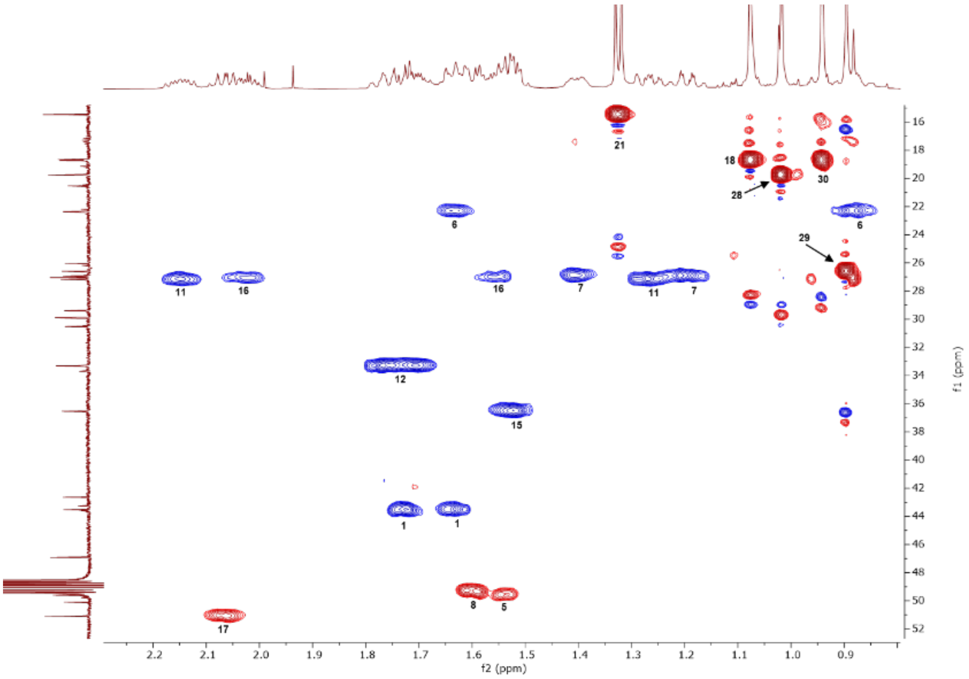

Figure S61. Detail of the ^{1}H/^{13}C HSQC spectrum of cycloprotobuxoline-D *N*3-*trans*-formamide (**8a**) and cycloprotobuxoline-D *N*3-*cis*-formamide (**8b**) (CD_3OD, 600/150 MHz).

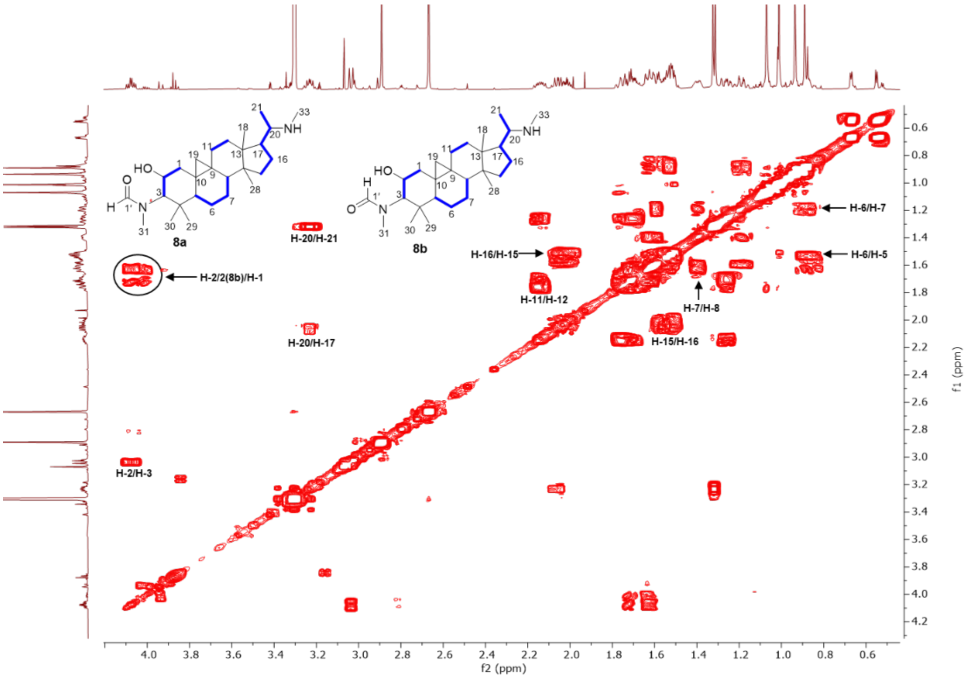

Figure S62. $^1H/^1H$ COSY spectrum (key correlations) of cycloprotobuxoline-D *N*3-*trans*-formamide (**8a**) and cycloprotobuxoline-D *N*3-*cis*-formamide (**8b**) (CD_3OD, 600 MHz).

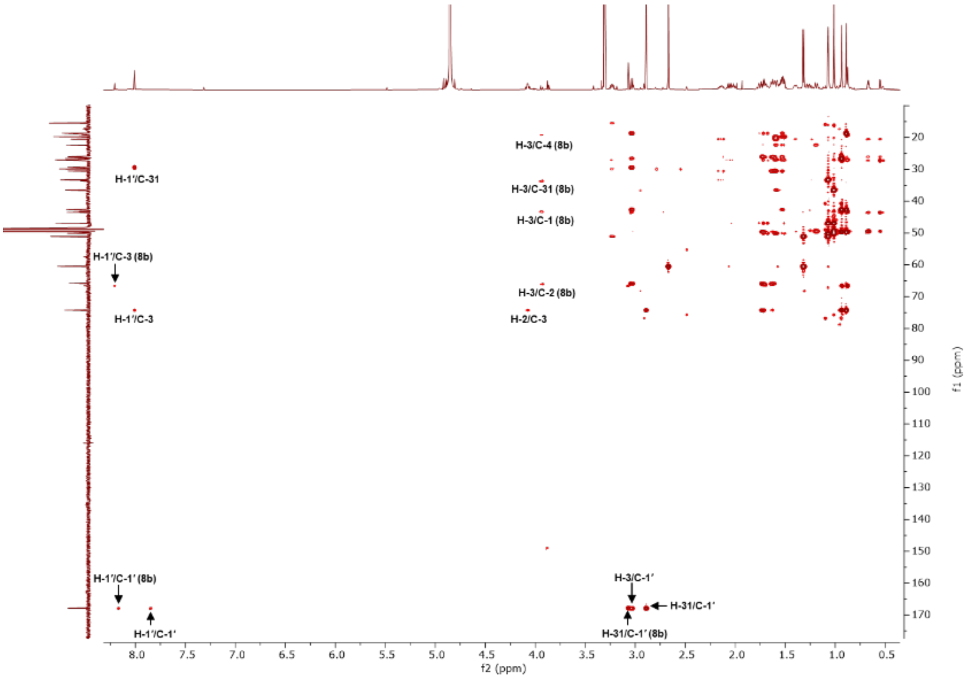

Figure S63. $^1H/^{13}C$ HMBC spectrum (key correlations) of cycloprotobuxoline-D *N*3-*trans*-formamide (**8a**) and cycloprotobuxoline-D *N*3-*cis*-formamide (**8b**) (CD_3OD, 600/150 MHz).

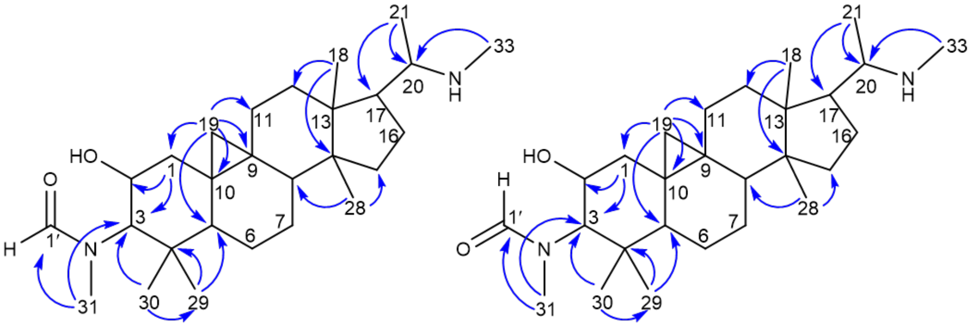

Figure S64. Key HMBC (blue arrows) correlations of cycloprotobuxoline-D *N*3-*trans*-formamide (**8a**) and cycloprotobuxoline-D *N*3-*cis*-formamide (**8b**).

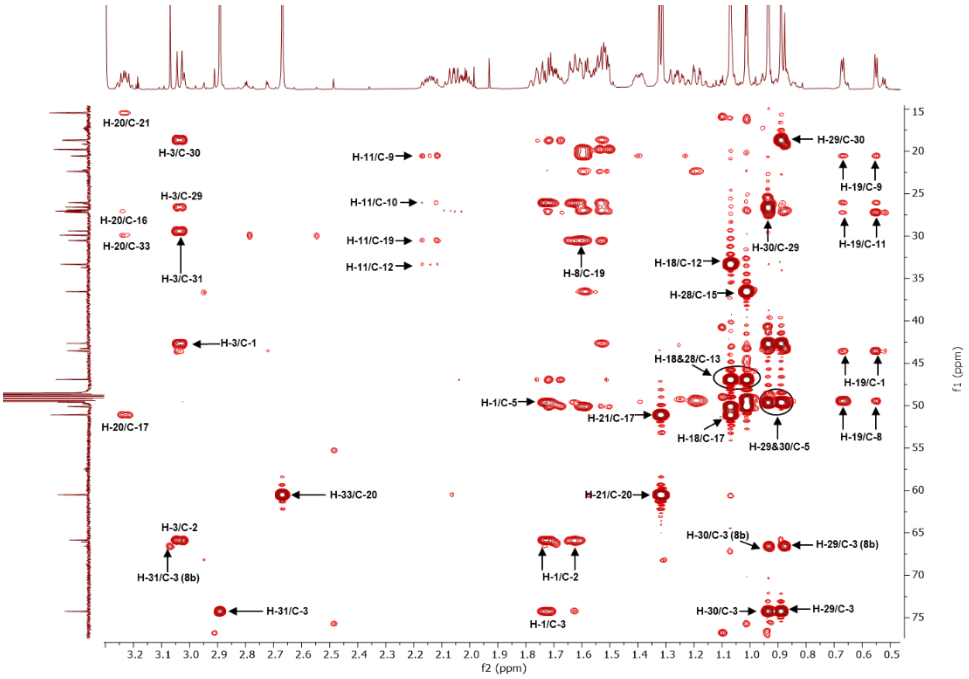

Figure S65. Detail of the $^{1}H/^{13}C$ HMBC spectrum (key correlations) of cycloprotobuxoline-D *N*3-*trans*-formamide (**8a**) and cycloprotobuxoline-D *N*3-*cis*-formamide (**8b**) (CD_3OD, 600/150 MHz).

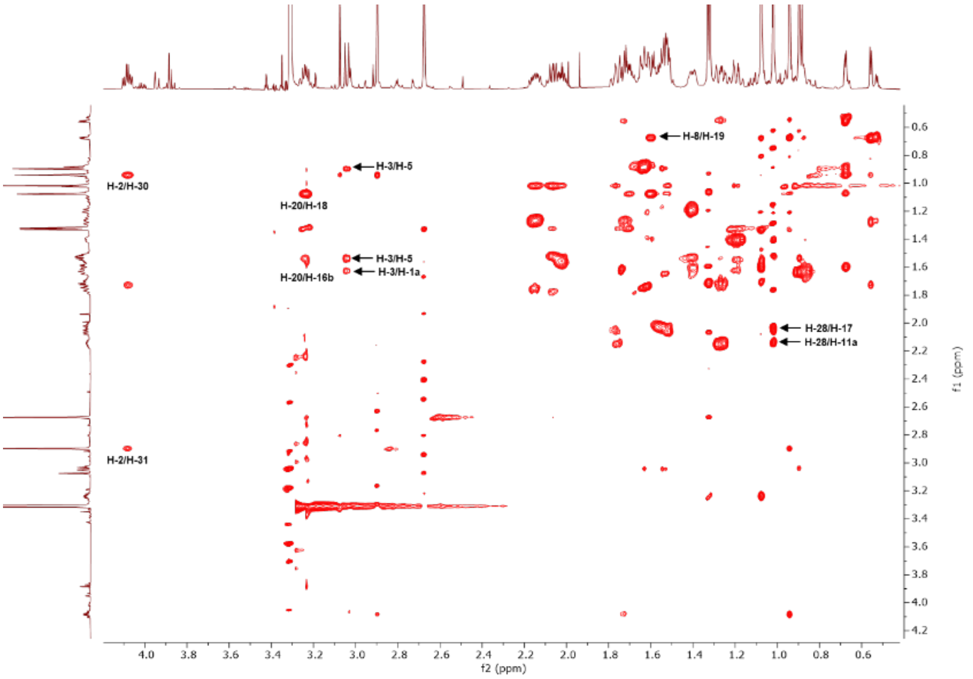

Figure S66. ^{1}H/^{1}H NOESY spectrum (key correlations) of cycloprotobuxoline-D *N*3-*trans*-formamide (**8a**) and cycloprotobuxoline-D *N*3-*cis*-formamide (**8b**) (CD_3OD, 600 MHz).

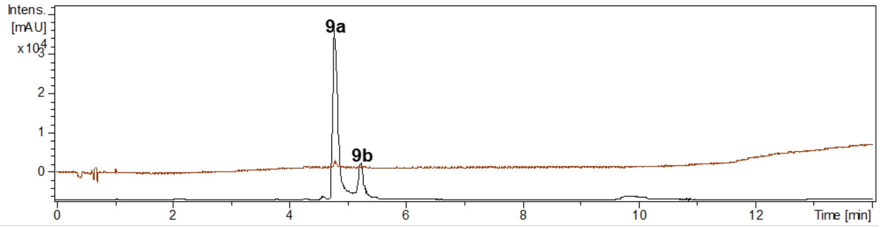

Figure S67. UHPLC/+ESI-QqTOF-MS/MS chromatogram of 16a-hydroxycycloprotobuxoline-C *N*3-*trans*-formamide (**9a**) and 16a-hydroxycycloprotobuxoline-C *N*3-*cis*-formamide (**9b**). Base peak chromatogram 200.0000–1000.0000 +All MS (black); UV chromatogram 200-400 nm (red).

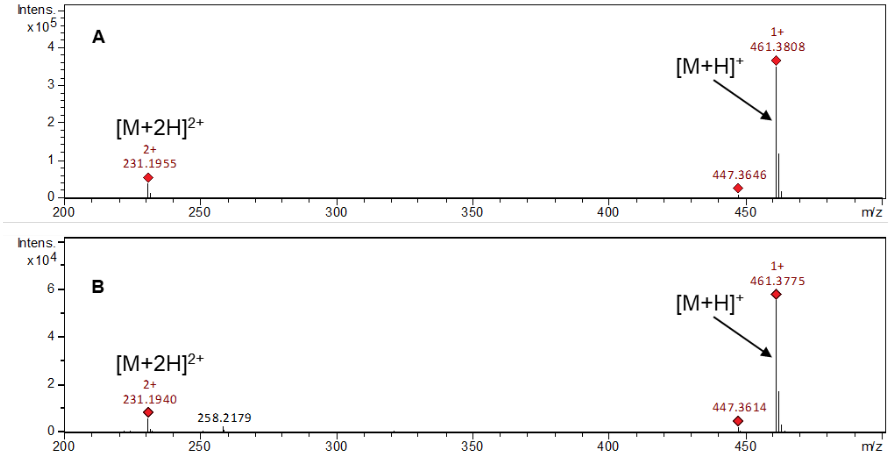

Figure S68. +ESI-QqTOF-MS spectrum of 16a-hydroxycycloprotobuxoline-C *N*3-*trans*-formamide (**9a**) (A); *m*/*z* 461.3808 $[M+H]^+$, 231.1955 $[M+2H]^{2+}$ and 16a-hydroxycycloprotobuxoline-C *N*3-*cis*-formamide (**9b**) (B); *m*/*z* 461.3775 $[M+H]^+$, 231.1940 $[M+2H]^{2+}$.

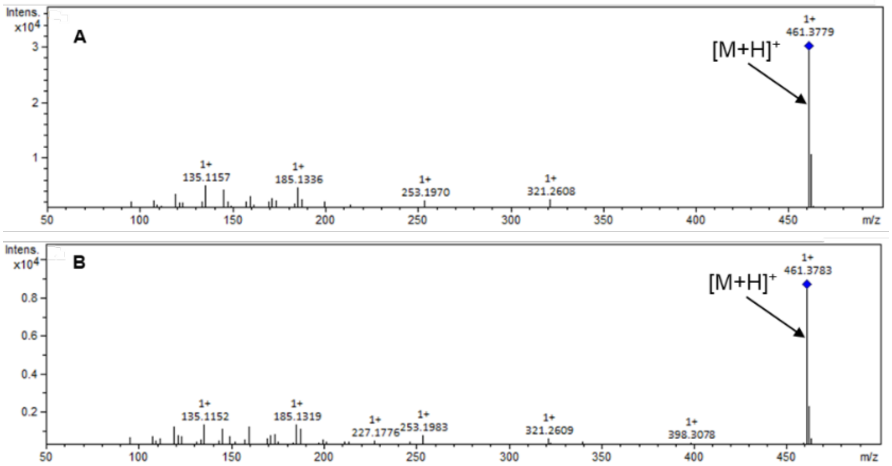

Figure S69. +ESI-QqTOF MS/MS spectrum of16a-hydroxycycloprotobuxoline-C *N*3-*trans*-formamide (**9a**) (A) and 16a-hydroxycycloprotobuxoline-C *N*3-*cis*-formamide (**9b**) (B).

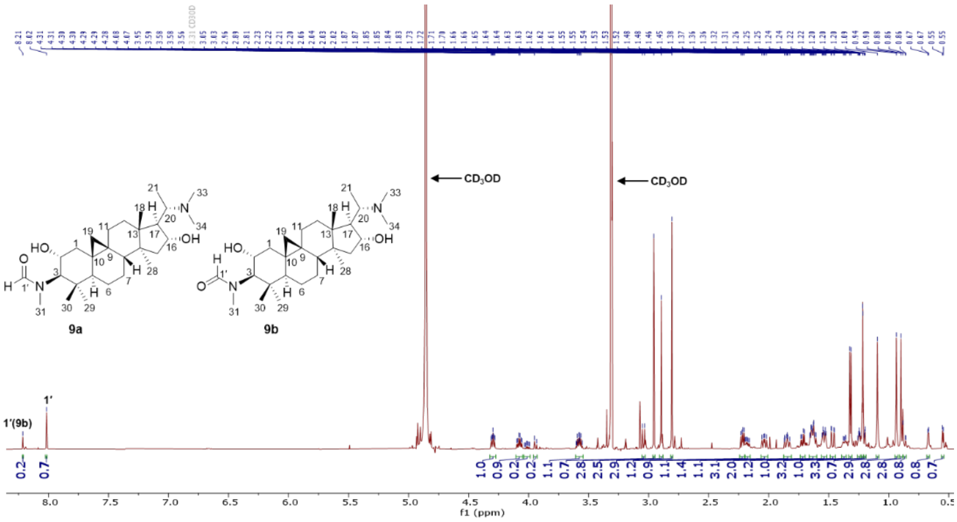

Figure S70. ^{1}H NMR spectrum of 16a-hydroxycycloprotobuxoline-C *N*3-*trans*-formamide (**9a**) and 16a-hydroxycycloprotobuxoline-C *N*3-*cis*-formamide (**9b**) (CD_3OD, 600 MHz).

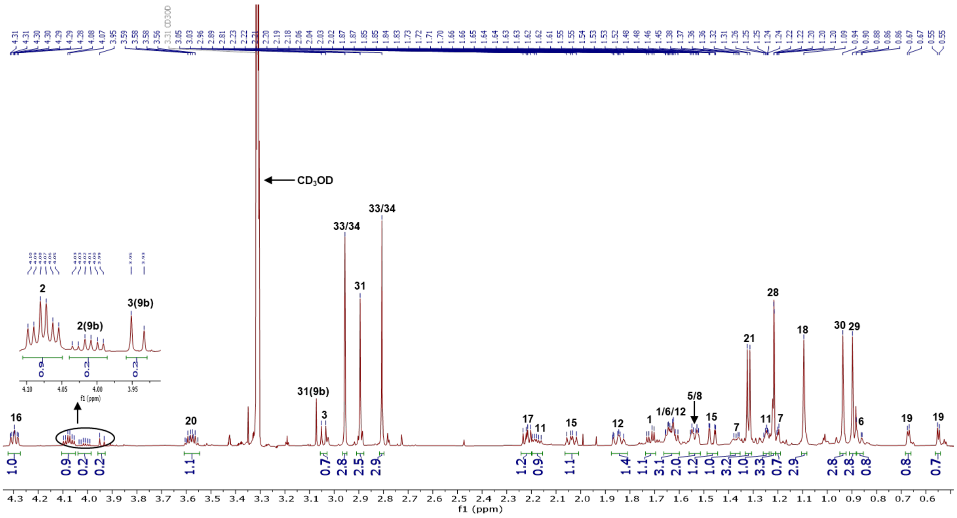

Figure S71. Detail of the ^{1}H NMR spectrum of 16a-hydroxycycloprotobuxoline-C *N*3-*trans*-formamide (**9a**) and 16a-hydroxycycloprotobuxoline-C *N*3-*cis*-formamide (**9b**) (CD_3OD, 600 MHz).

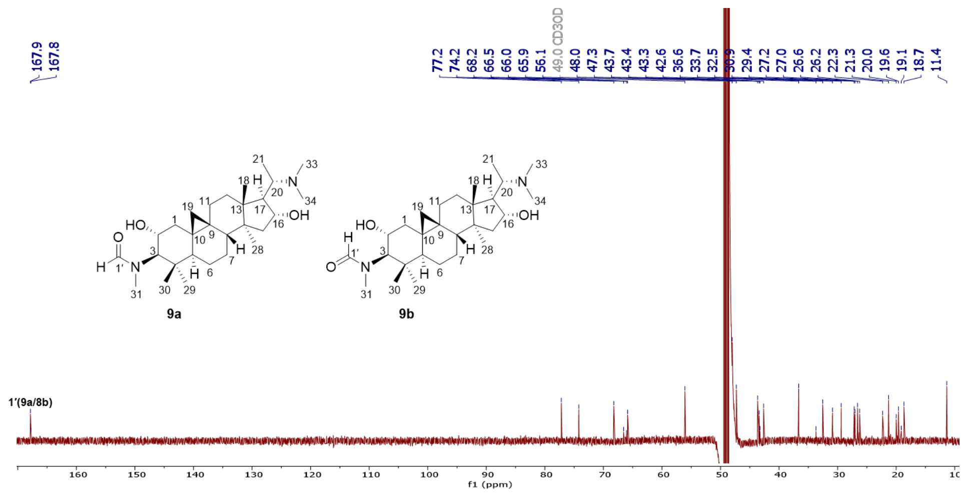

Figure S72. ^{13}C NMR spectrum of 16a-hydroxycycloprotobuxoline-C N_3-*trans*-formamide (**9a**) and 16a-hydroxycycloprotobuxoline-C N_3-*cis*-formamide (**9b**) (CD_3OD, 150 MHz).

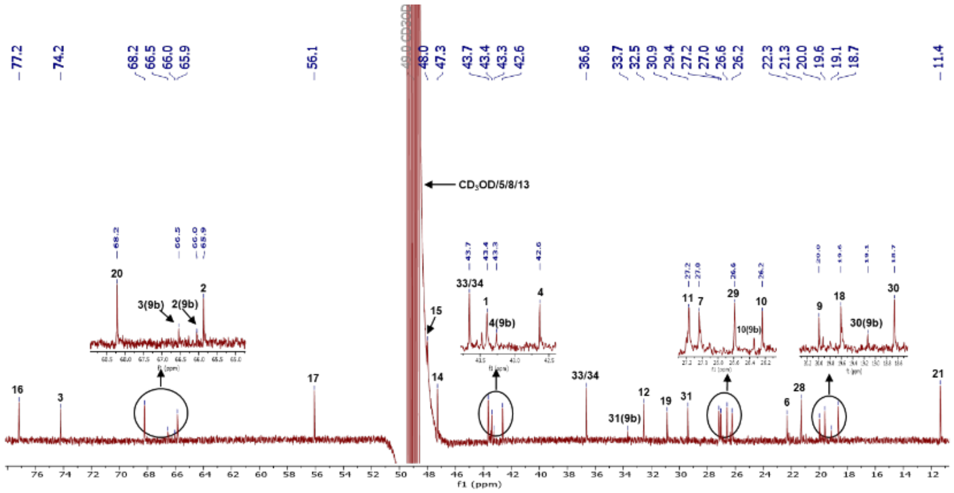

Figure S73. Detail of the ^{13}C NMR spectrum of 16a-hydroxycycloprotobuxoline-C N_3-*trans*-formamide (**9a**) and 16a-hydroxycycloprotobuxoline-C N_3-*cis*-formamide (**9b**) (CD_3OD, 150 MHz).

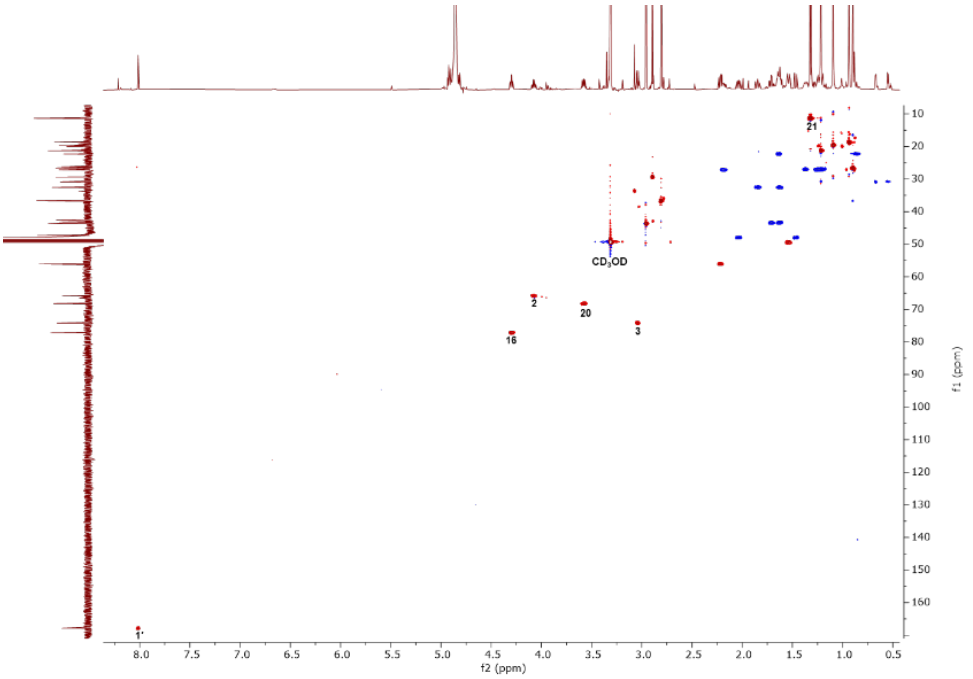

Figure S74. ^{1}H/^{13}C HSQC spectrum of 16a-hydroxycycloprotobuxoline-C N_3-*trans*-formamide (**9a**) and 16a-hydroxycycloprotobuxoline-C N_3-*cis*-formamide (**9b**) (CD_3OD, 600/150 MHz).

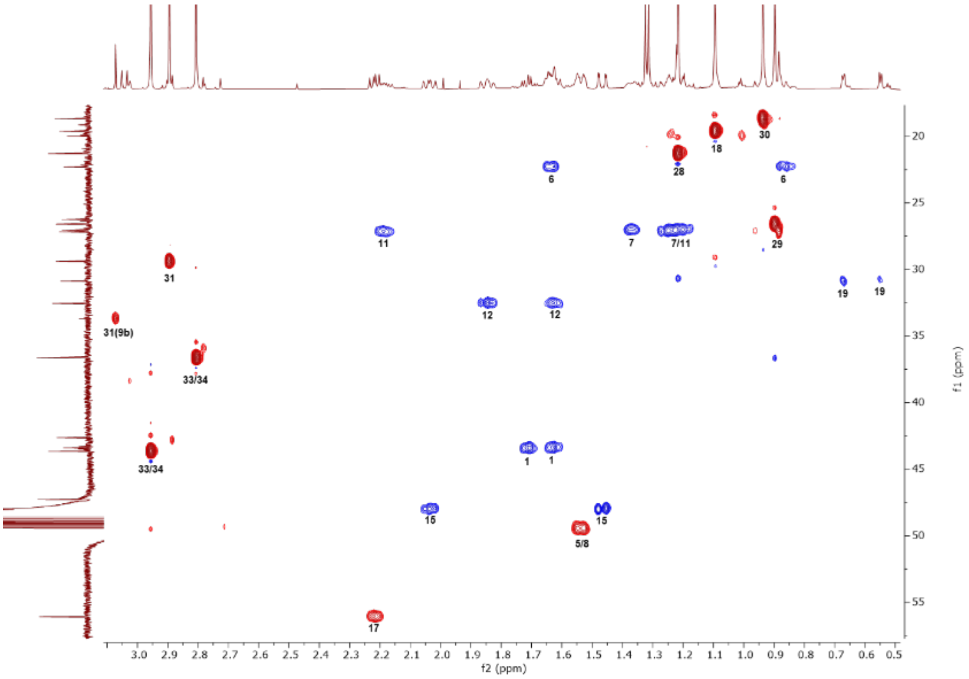

Figure S75. Detail of the $^{1}H/^{13}C$ HSQC spectrum of 16a-hydroxycycloprotobuxoline-C *N*3-*trans*-formamide (**9a**) and 16a-hydroxycycloprotobuxoline-C *N*3-*cis*-formamide (**9b**) (CD_3OD, 600/150 MHz).

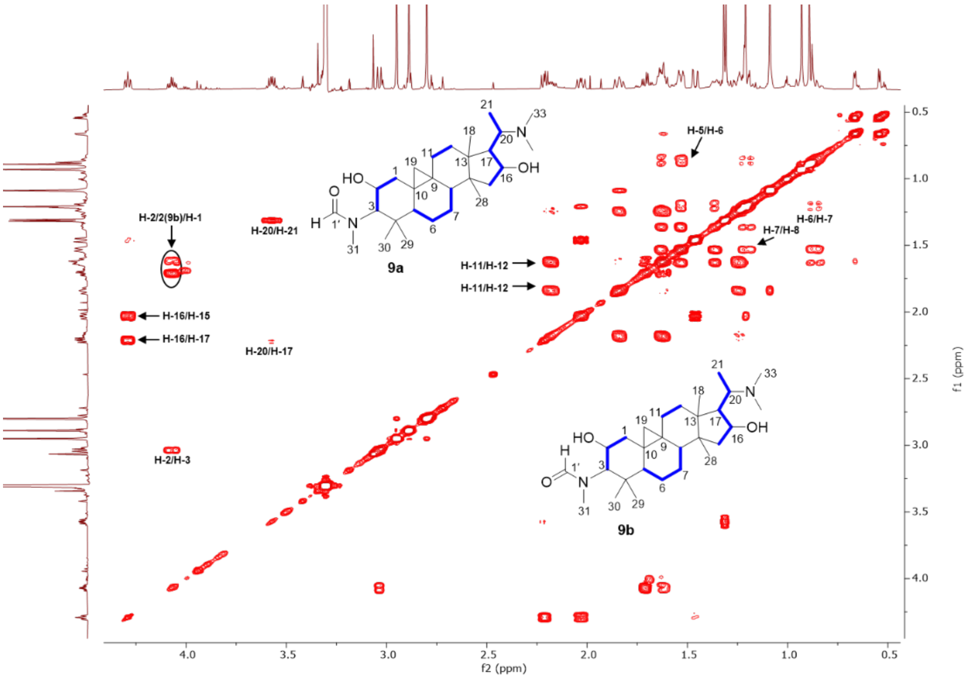

Figure S76. $^{1}H/^{1}H$ COSY spectrum (key correlations) of 16a-hydroxycycloprotobuxoline-C *N*3-*trans*-formamide (**9a**) and 16a-hydroxycycloprotobuxoline-C *N*3-*cis*-formamide (**9b**) (CD_3OD, 600 MHz).

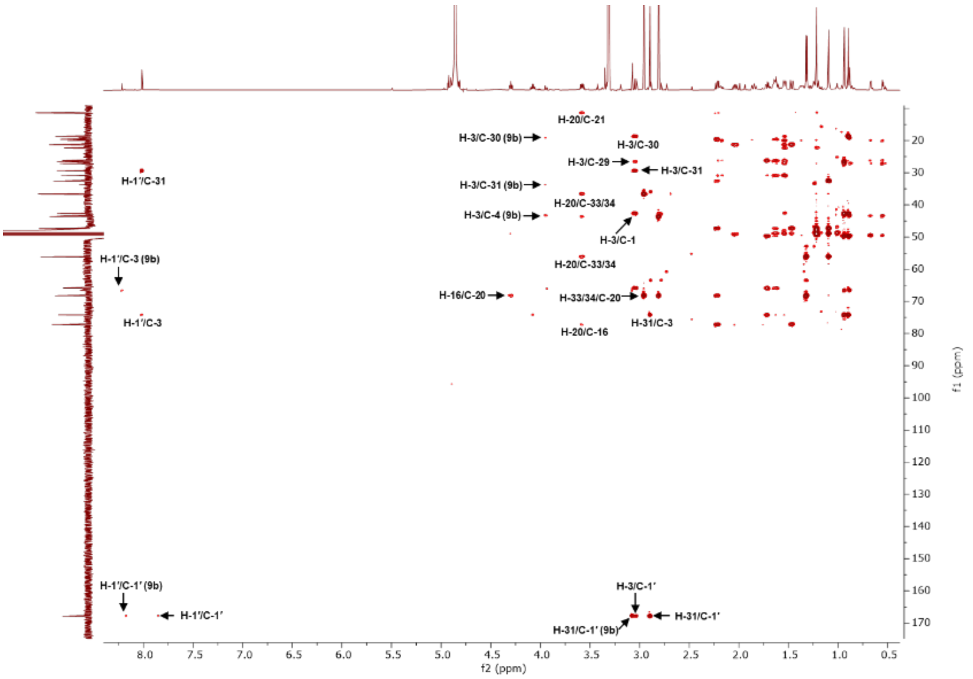

Figure S77. $^{1}H/^{13}C$ HMBC spectrum (key correlations) of 16a-hydroxycycloprotobuxoline-C N_3-*trans*-formamide (**9a**) and 16a-hydroxycycloprotobuxoline-C N_3-*cis*-formamide (**9b**) (CD_3OD, 600/150 MHz).

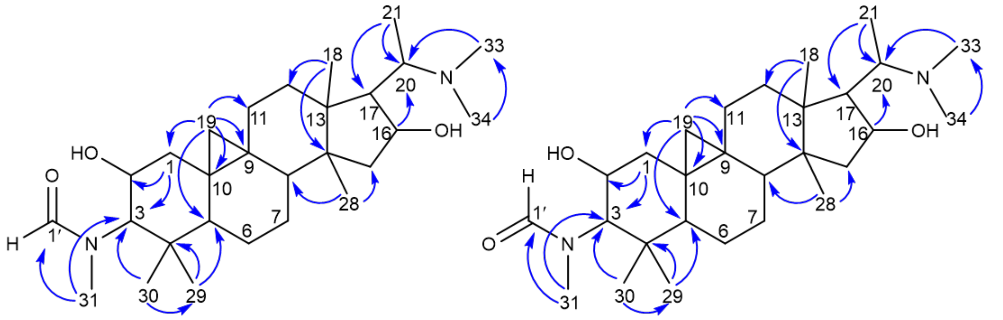

Figure S78. Key HMBC (blue arrows) correlations of 16a-hydroxycycloprotobuxoline-C N_3-*trans*-formamide (**9a**) and 16a-hydroxycycloprotobuxoline-C N_3-*cis*-formamide (**9b**).

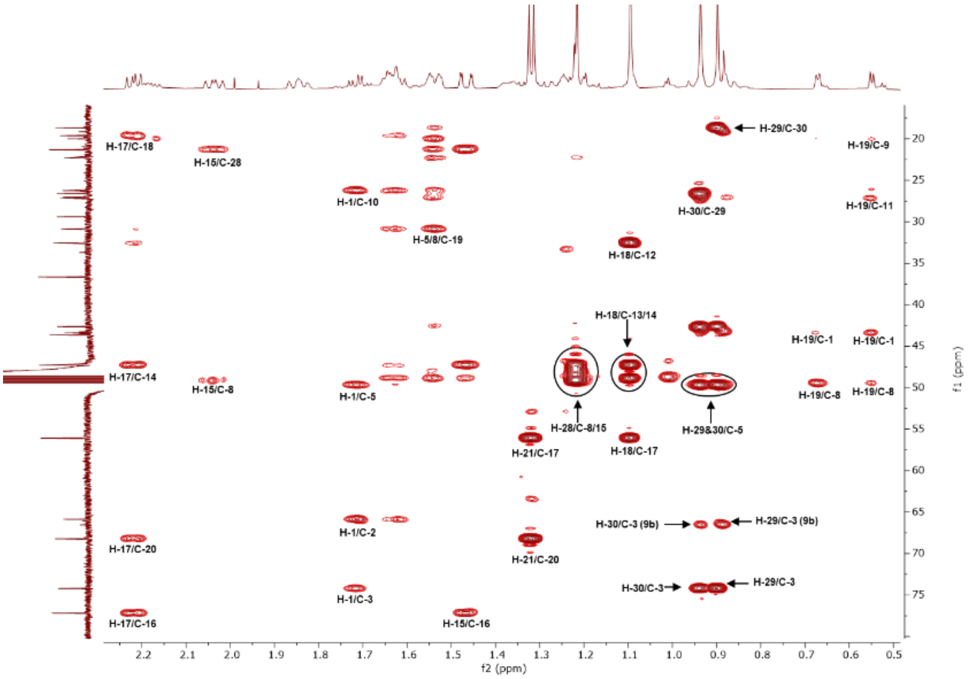

Figure S79. Detail of the $^{1}H/^{13}C$ HMBC spectrum (key correlations) of 16a-hydroxycycloprotobuxoline-C *N*3-*trans*-formamide (**9a**) and 16a-hydroxycycloprotobuxoline-C *N*3-*cis*-formamide (**9b**) (CD_3OD, 600/150 MHz).

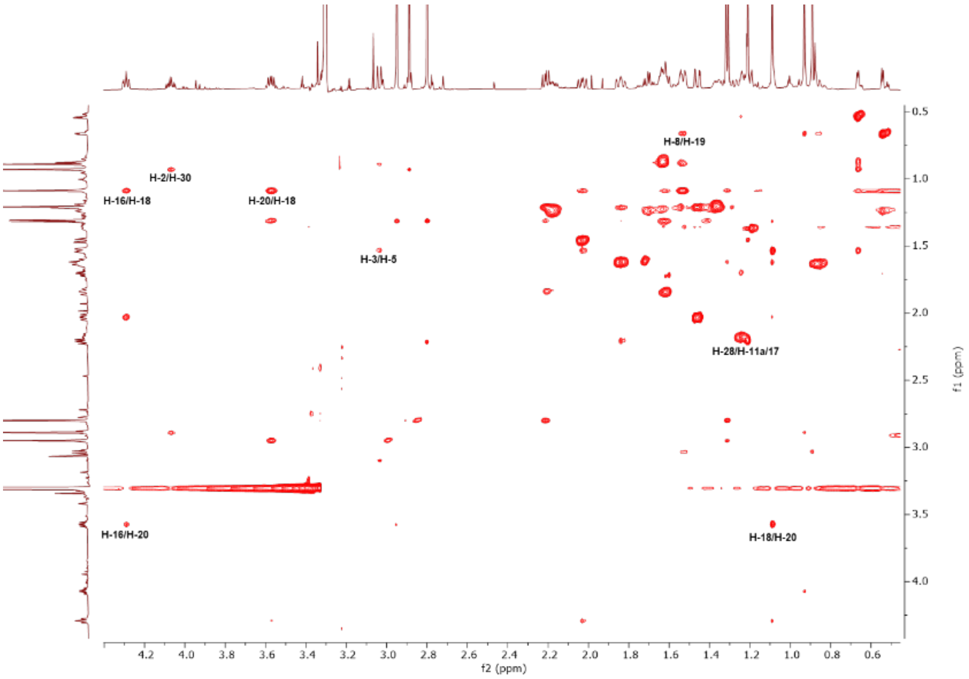

Figure S80. $^{1}H/^{1}H$ NOESY spectrum (key correlations) of 16a-hydroxycycloprotobuxoline-C *N*3-*trans*-formamide (**9a**) and 16a-hydroxycycloprotobuxoline-C *N*3-*cis*-formamide (**9b**) (CD_3OD, 600 MHz).

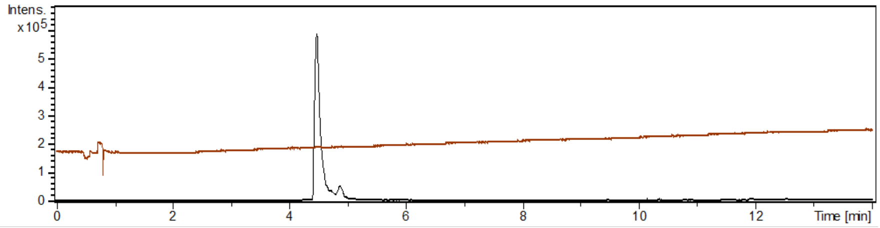

Figure S81. UHPLC/+ESI-QqTOF-MS/MS chromatogram of *N*3-demethyl cyclonataminol (**11**). Base peak chromatogram 200.0000–1000.0000 +All MS (black); UV chromatogram 200-400 nm (red).

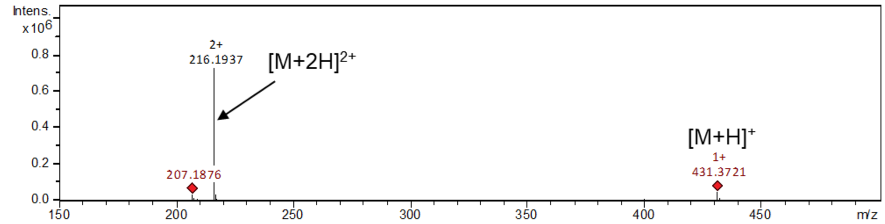

Figure S82. +ESI-QqTOF-MS spectrum of *N*3-demethyl cyclonataminol (**11**); *m/z* 431.3721 [M+H]$^+$, 216.1937 [M+2H]$^{2+}$.

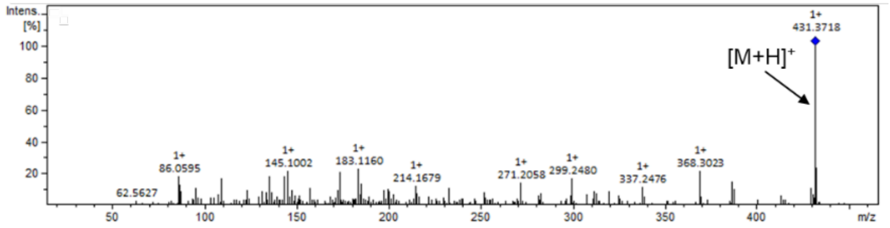

Figure S83. +ESI-QqTOF MS/MS spectrum of *N*3-demethyl cyclonataminol (**11**).

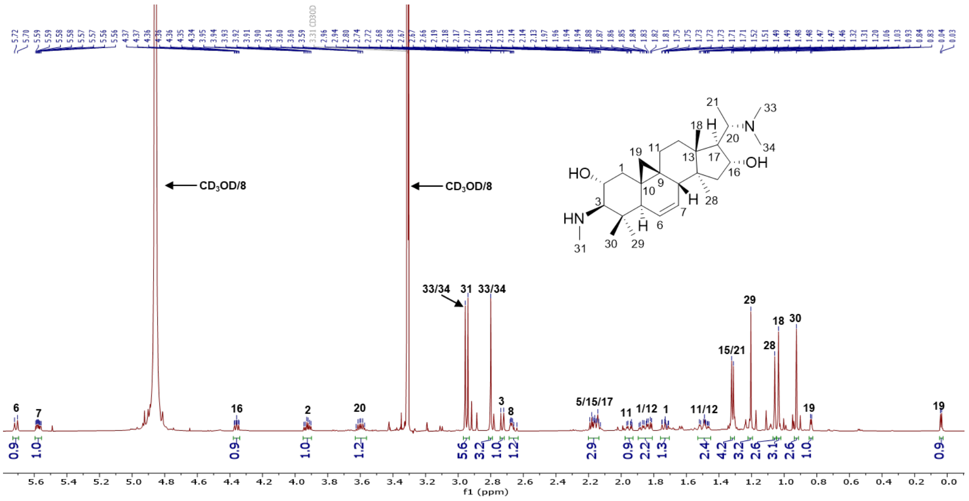

Figure S84. ^{1}H NMR spectrum of *N*3-demethyl cyclonataminol (**11**) (CD_3OD, 600 MHz).

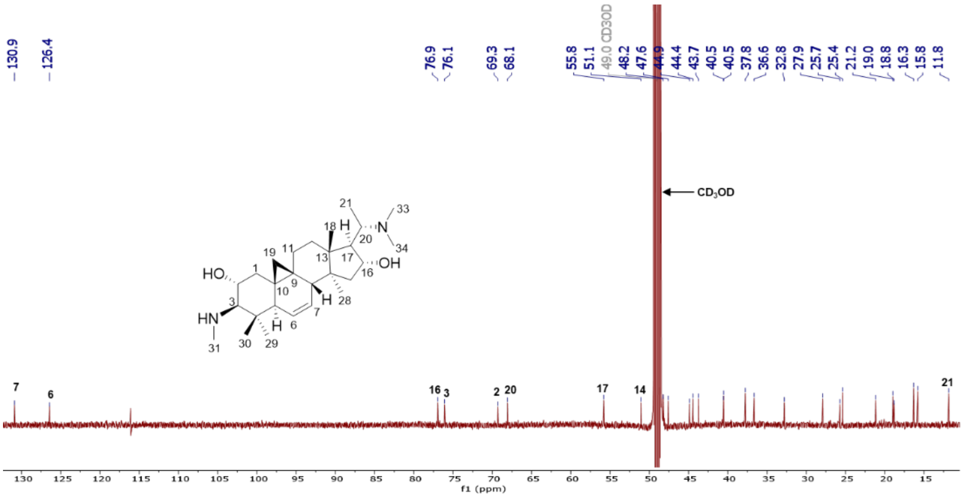

Figure S85. ^{13}C NMR spectrum of *N*3-demethyl cyclonataminol (**11**) (CD_3OD, 150 MHz).

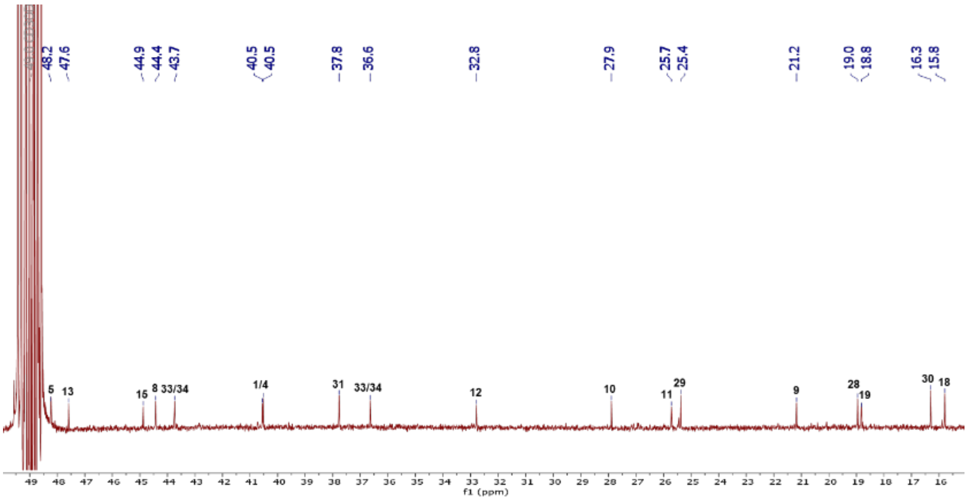

Figure S86. Detail of the ^{13}C NMR spectrum of *N*3-demethyl cyclonataminol (**11**) (CD_3OD, 150 MHz).

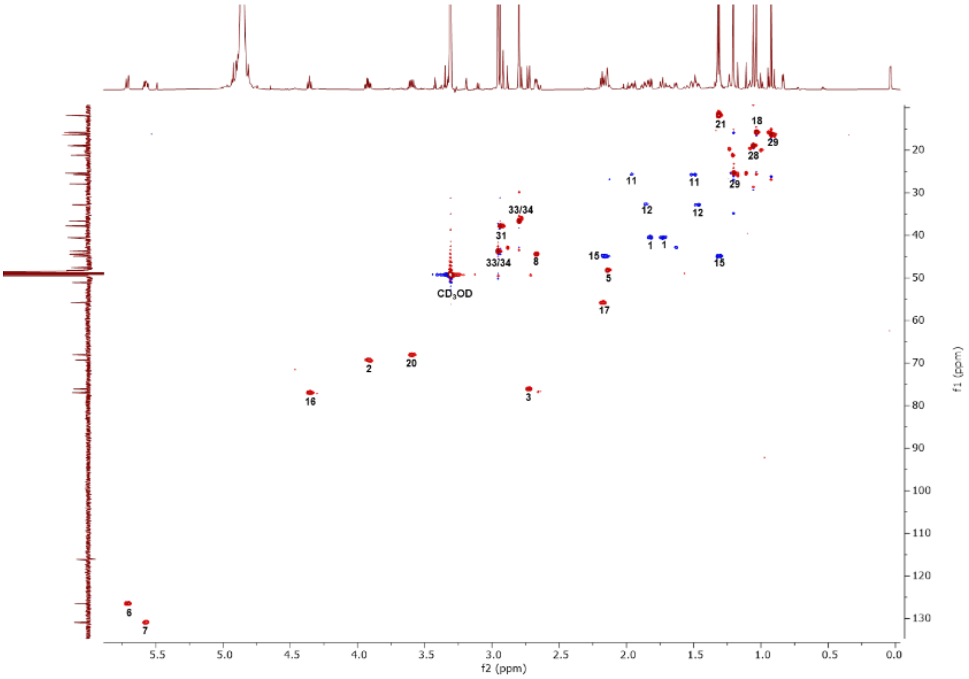

Figure S87. ^{1}H/^{13}C HSQC spectrum of *N*3-demethyl cyclonataminol (**11**) (CD_3OD, 600/150 MHz).

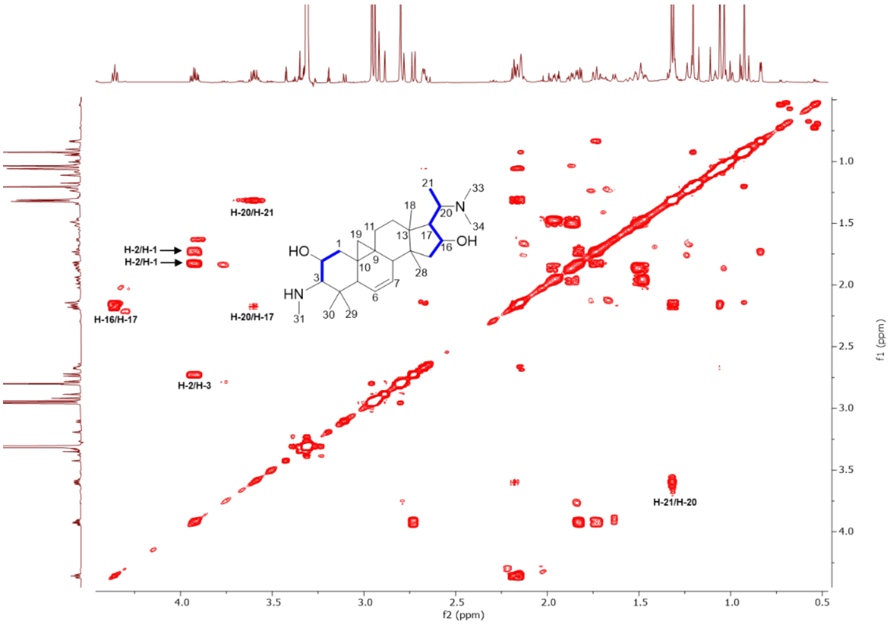

Figure S88. $^1H/^1H$ COSY spectrum (key correlations) of *N*3-demethyl cyclonataminol (**11**) (CD_3OD, 600 MHz).

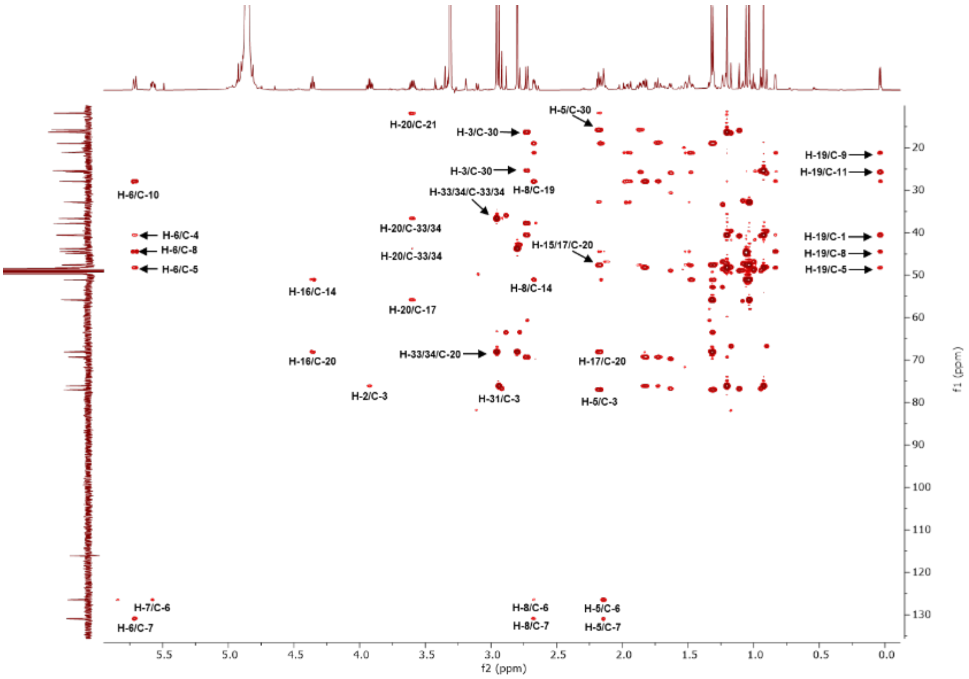

Figure S89. $^1H/^{13}C$ HMBC spectrum (key correlations) of *N*3-demethyl cyclonataminol (**11**) (CD_3OD, 600/150 MHz).

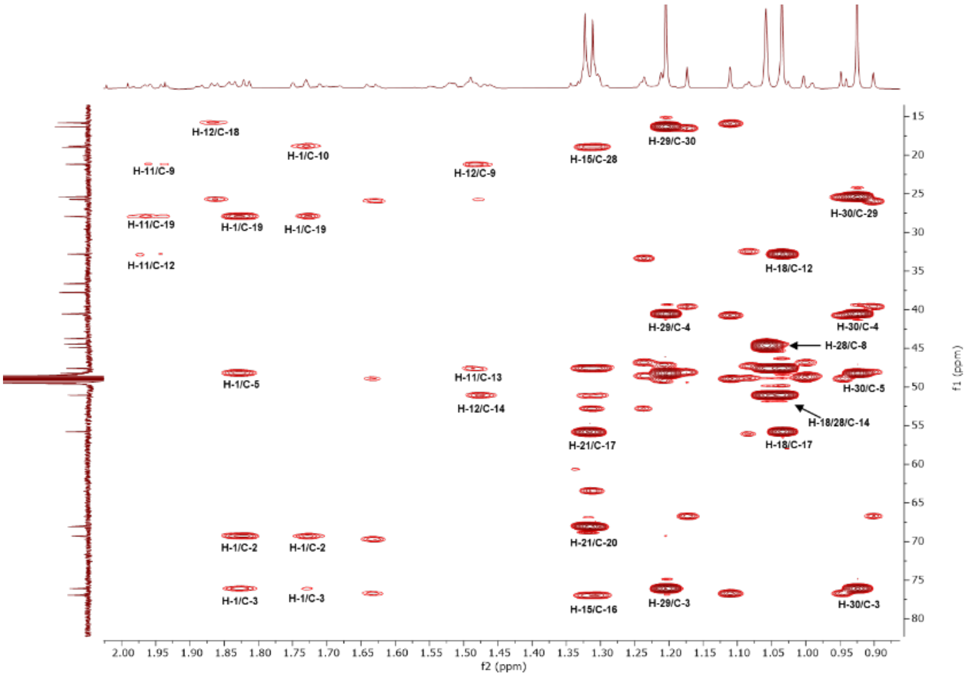

Figure S90. Detail of the $^{1}H/^{13}C$ HMBC spectrum (key correlations) of *N*3-demethyl cyclonataminol (**11**) (CD3OD, 600/150 MHz).

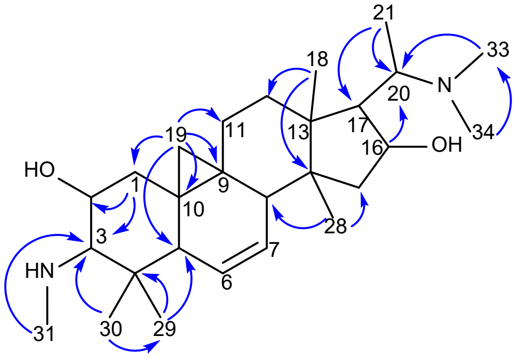

Figure S91. Key HMBC (blue arrows) correlations of *N*3-demethyl cyclonataminol (**11**).

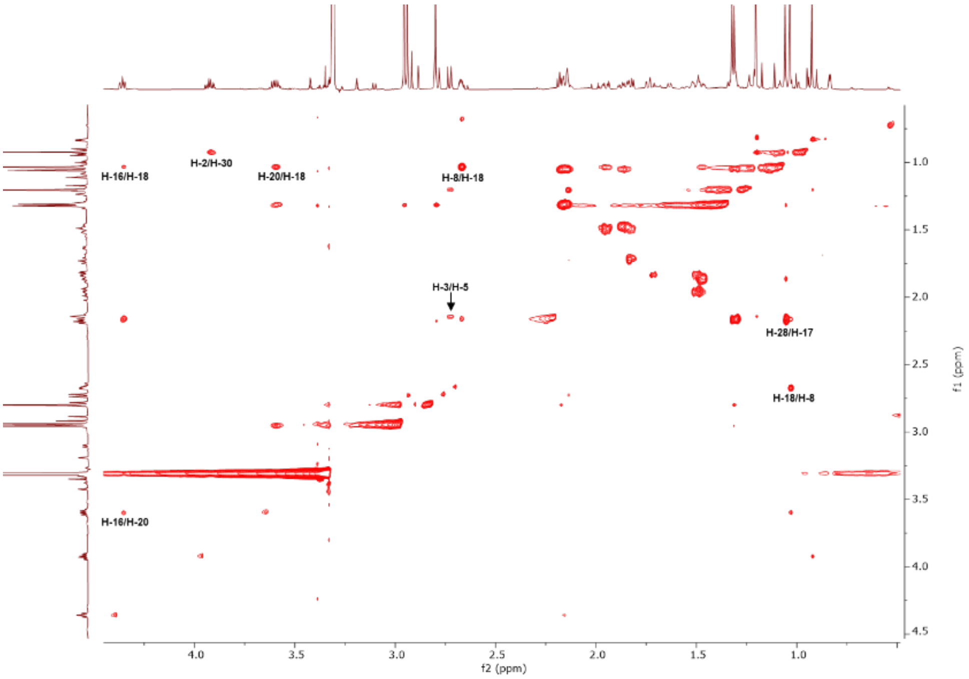

Figure S92. $^1H/^1H$ NOESY spectrum (key correlations) of *N*3-demethyl cyclonataminol (**11**) (CD_3OD, 600 MHz).

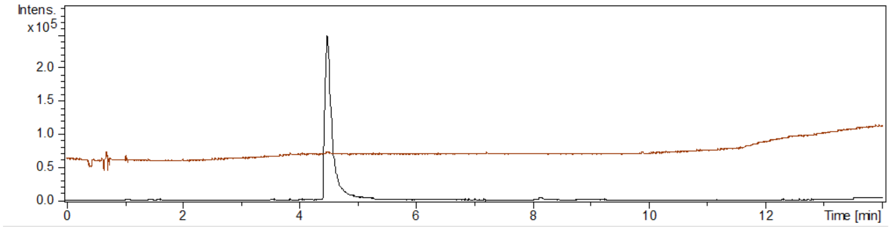

Figure S93. UHPLC/+ESI-QqTOF-MS/MS chromatogram of deoxycyclovirobuxeine-B (**12**). Base peak chromatogram 200.0000–1000.0000 +All MS (black); UV chromatogram 200-400 nm (red).

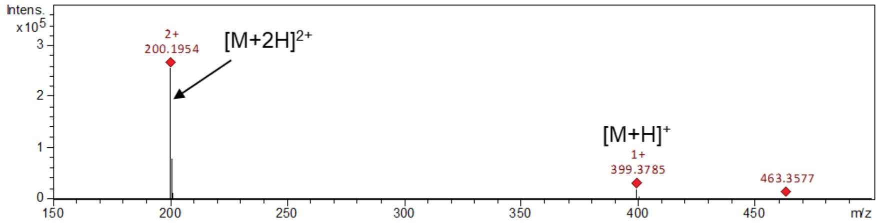

Figure S94. +ESI-QqTOF-MS spectrum of deoxycyclovirobuxeine-B (**12**); *m/z* 399.3785 $[M+H]^+$, 200.1954 $[M+2H]^{2+}$.

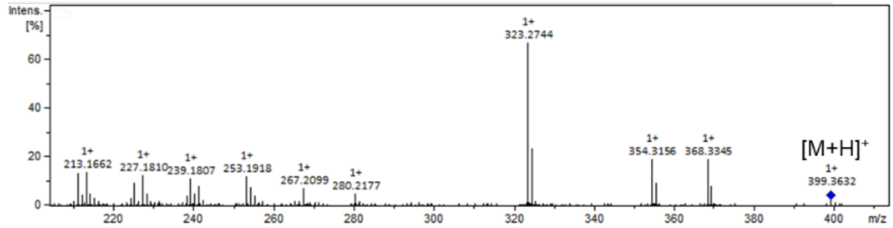

Figure S95. +ESI-QqTOF MS/MS spectrum of deoxycyclovirobuxeine-B (**12**).

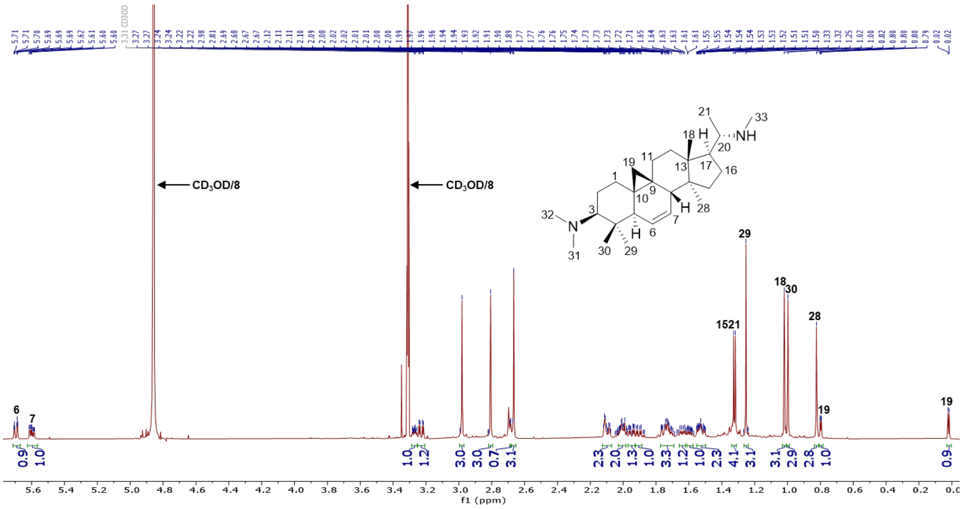

Figure S96. ^{1}H NMR spectrum of deoxycyclovirobuxeine-B (**12**) (CD_3OD, 600 MHz).

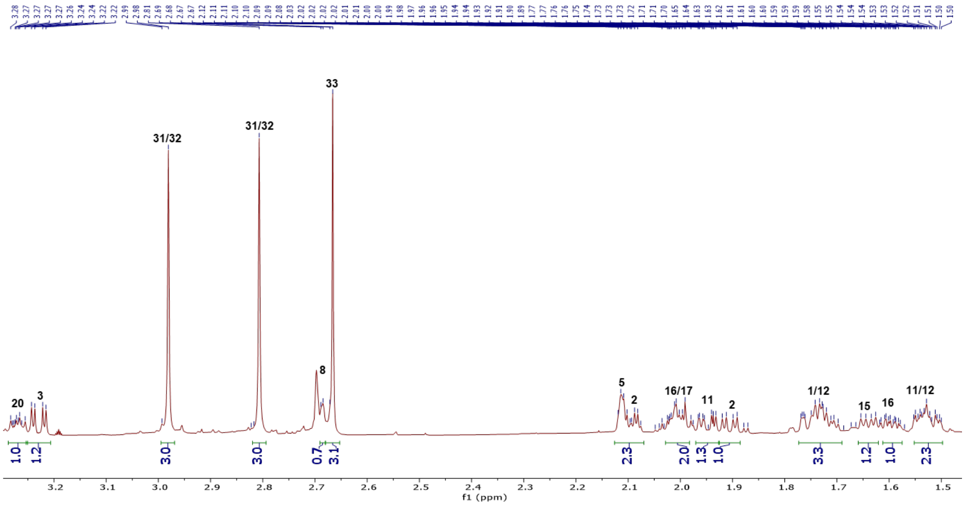

Figure S97. Detail of the ^{1}H NMR spectrum of deoxycyclovirobuxeine-B (**12**) (CD_3OD, 600 MHz).

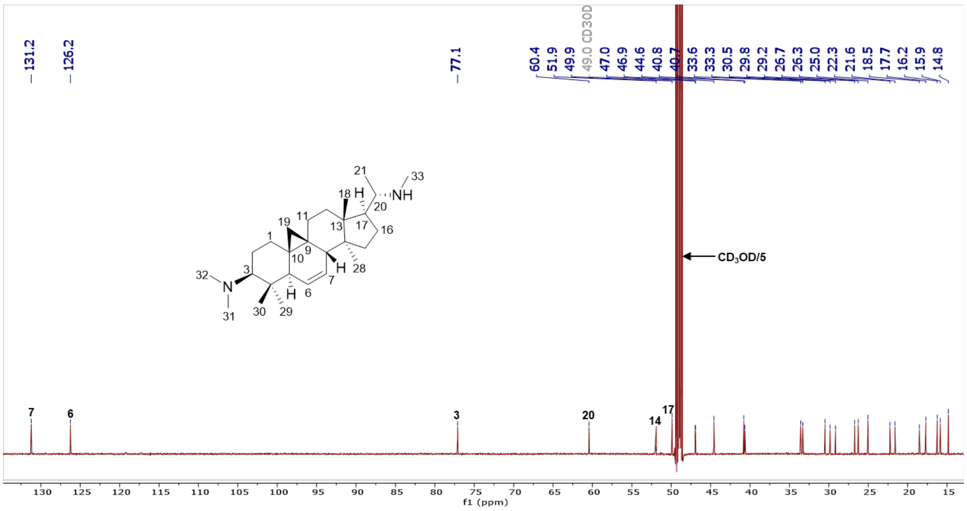

Figure S98. ^{13}C NMR spectrum of deoxycyclovirobuxeine-B (**12**) (CD_3OD, 150 MHz).

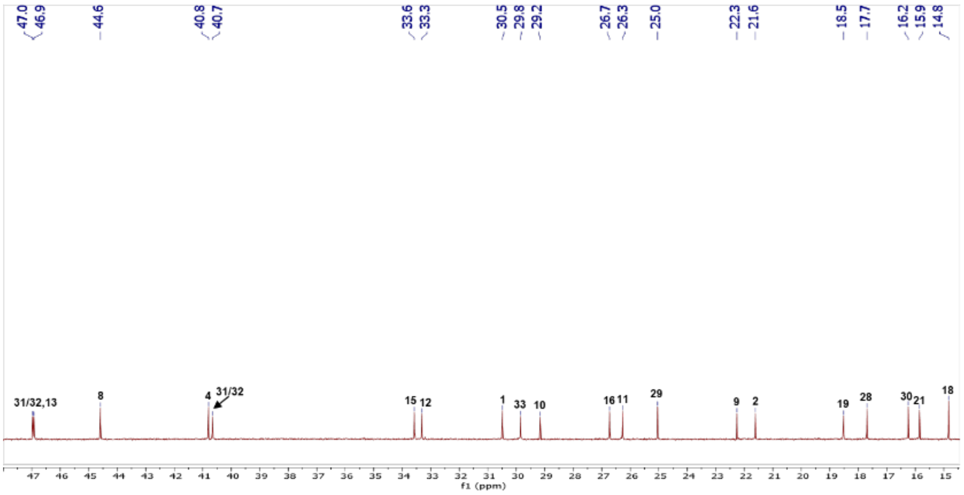

Figure S99. Detail of the ^{13}C NMR spectrum of deoxycyclovirobuxeine-B (**12**) (CD_3OD, 150 MHz).

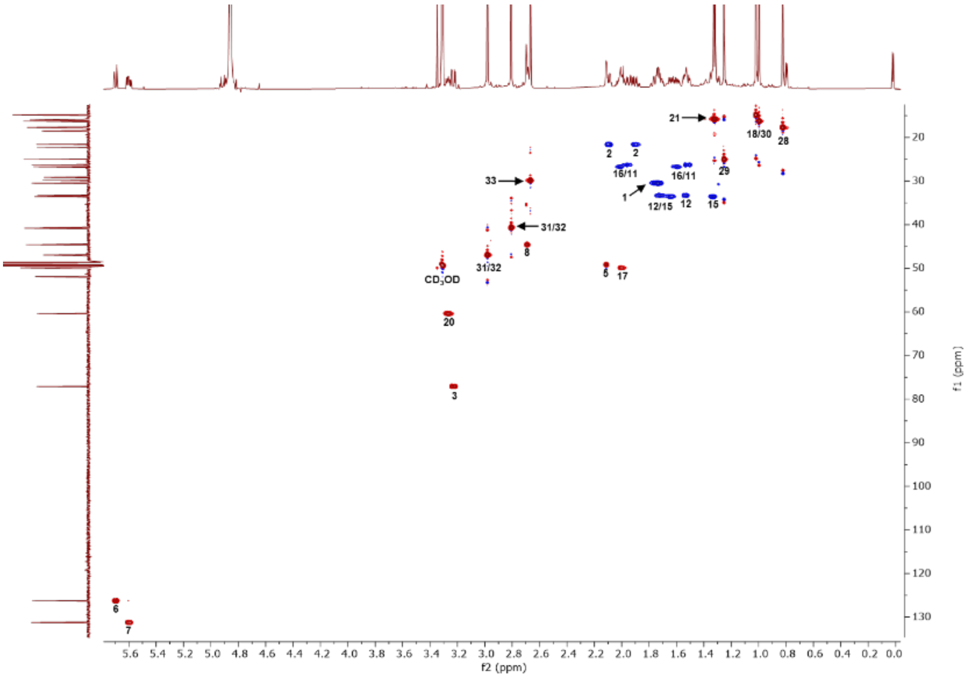

Figure S100. $^{1}H/^{13}C$ HSQC spectrum of deoxycyclovirobuxeine-B (**12**) (CD_3OD, 600/150 MHz).

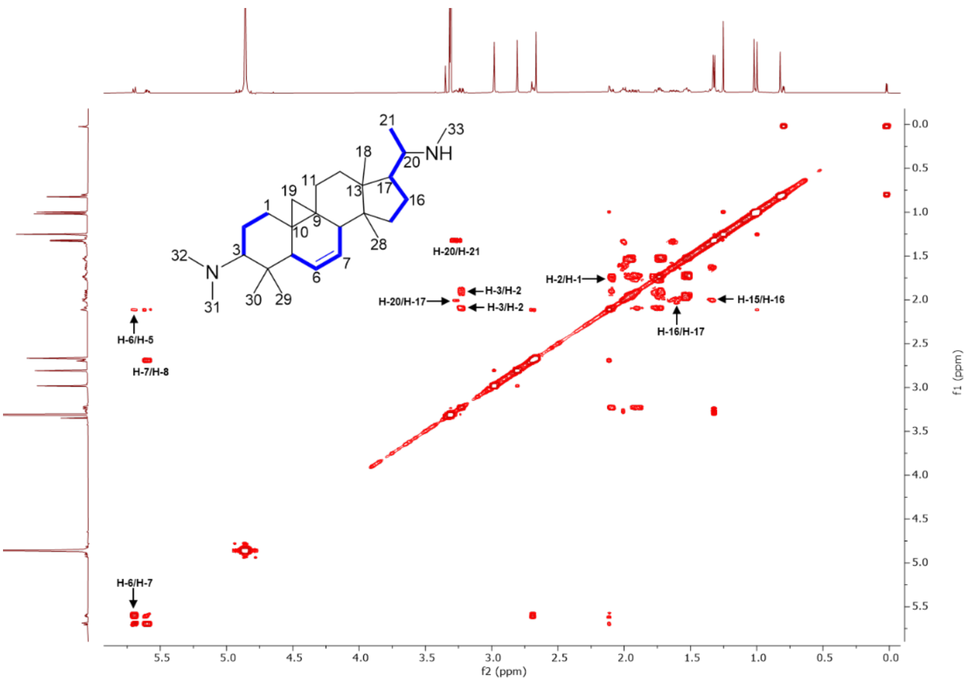

Figure S101. ${}^{1}H/{}^{1}H$ COSY spectrum (key correlations) of deoxycyclovirobuxeine-B (**12**) (CD_3OD, 600 MHz).

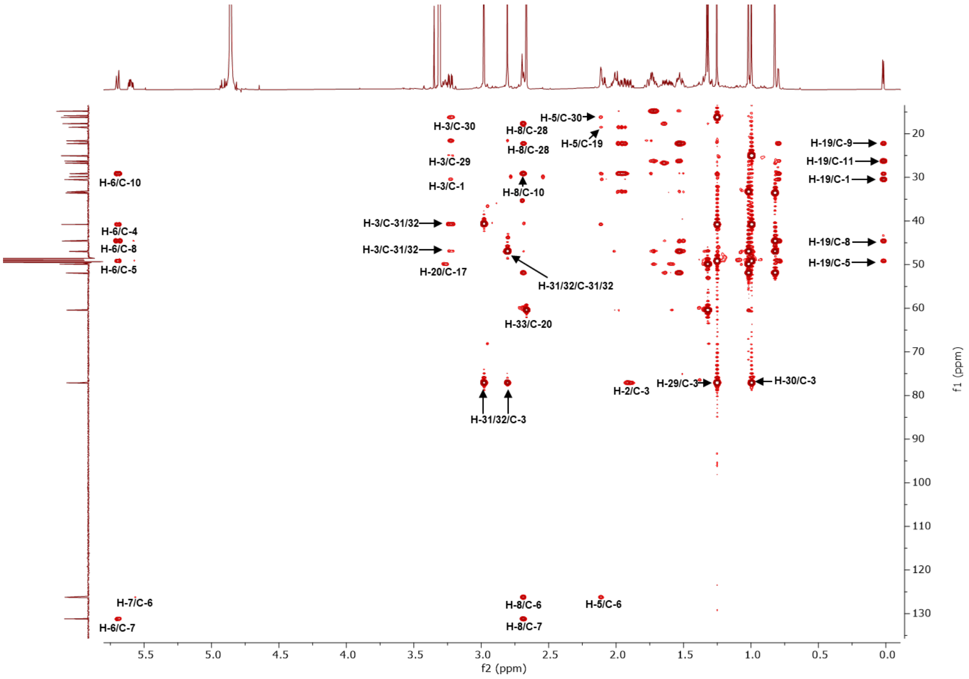

Figure S102. ${}^{1}H/{}^{13}C$ HMBC spectrum (key correlations) of deoxycyclovirobuxeine-B (**12**) (CD_3OD, 600/150 MHz).

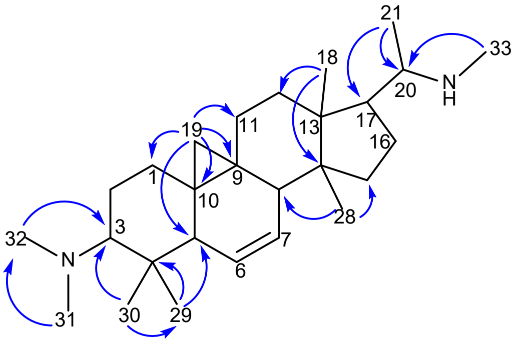

Figure S103. Key HMBC (blue arrows) correlations of deoxycyclovirobuxeine-B (**12**).

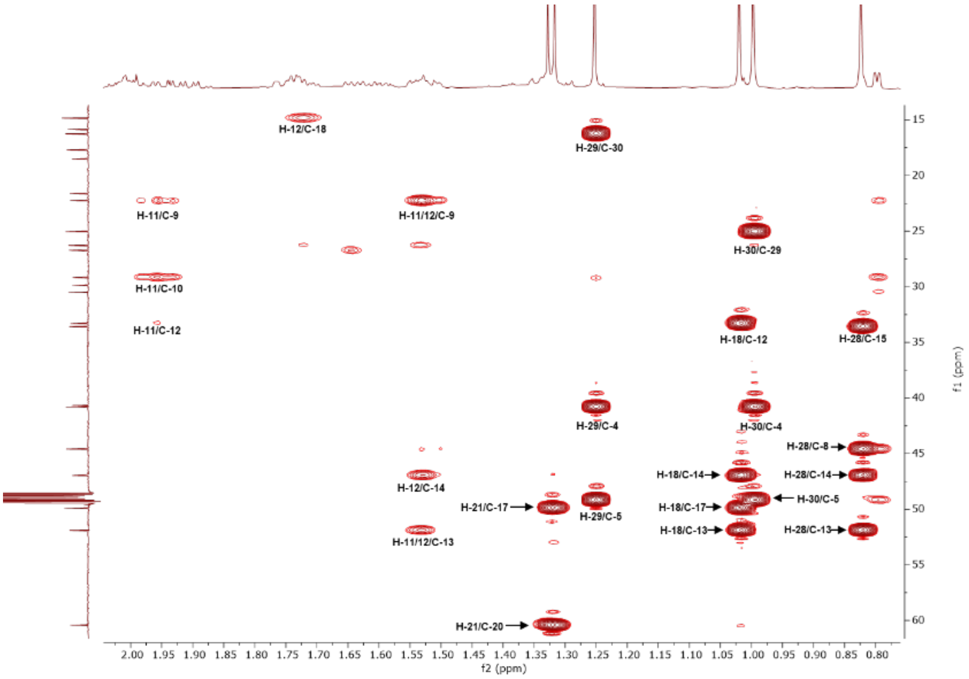

Figure S104. Detail of the $^1H/^{13}C$ HMBC spectrum (key correlations) of deoxycyclovirobuxeine-B (**12**) (CD_3OD, 600/150 MHz).

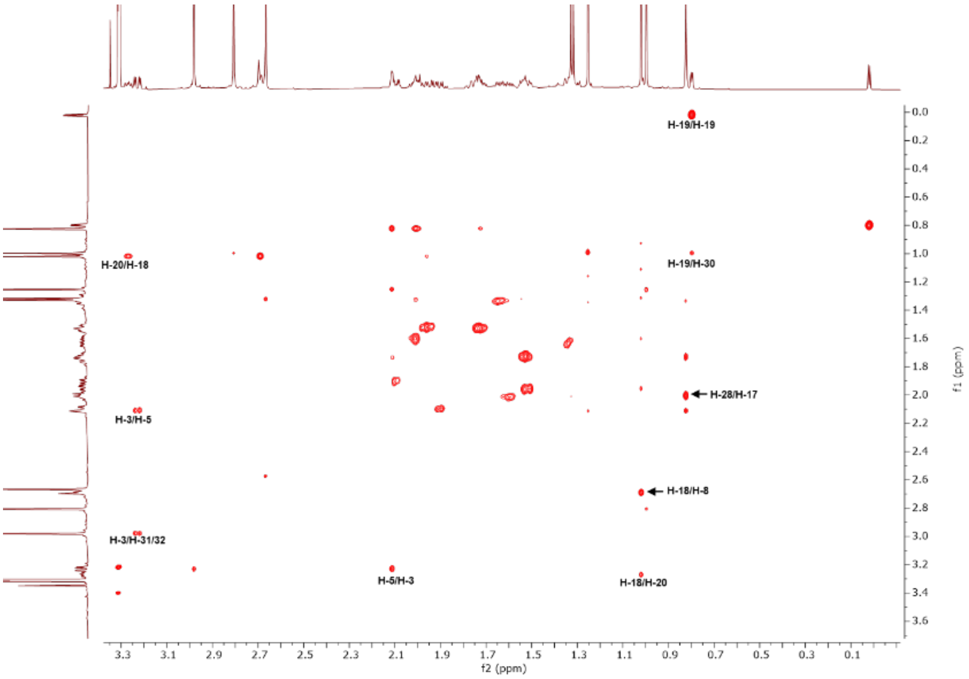

Figure S105. $^1H/^1H$ NOESY spectrum (key correlations) of deoxycyclovirobuxeine-B (**12**) (CD_3OD, 600 MHz).

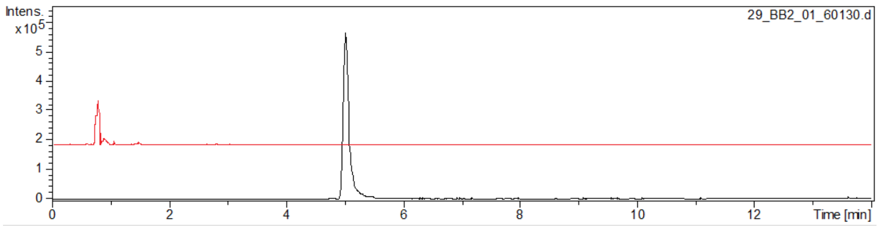

Figure S106. UHPLC/+ESI-QqTOF-MS/MS chromatogram of *N*20-demethyl deoxycyclobuxoxazine A (**15**). Base peak chromatogram 200.0000–1000.0000 +All MS (black); UV chromatogram 200-400 nm (red).

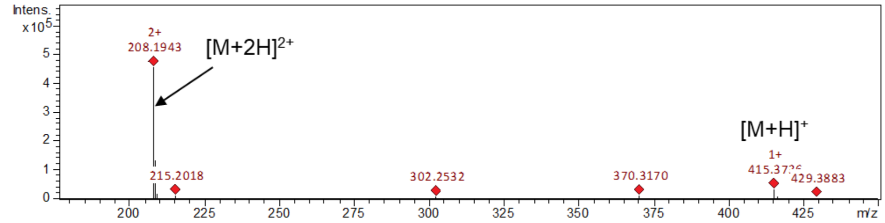

Figure S107. +ESI-QqTOF-MS spectrum of *N*20-demethyl deoxycyclobuxoxazine A (**15**); *m/z* 415.3736 [M+H]+, 208.1943 [M+2H]2+.

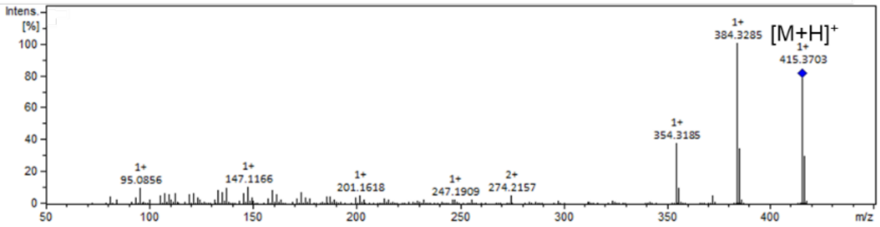

Figure S108. +ESI-QqTOF MS/MS spectrum of *N*20-demethyl deoxycyclobuxoxazine A (**15**).

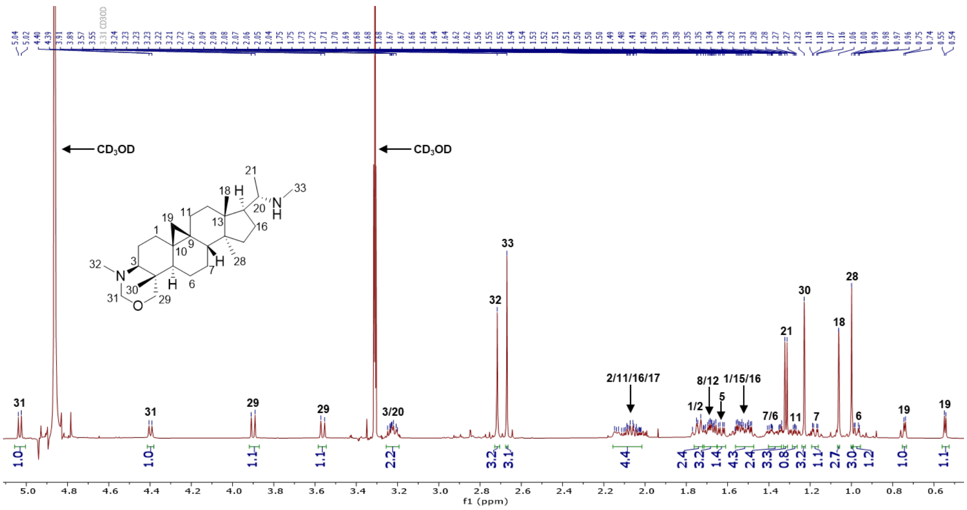

Figure S109. ^{1}H NMR spectrum of *N*20-demethyl deoxycyclobuxoxazine A (**15**) (CD_3OD, 600 MHz).

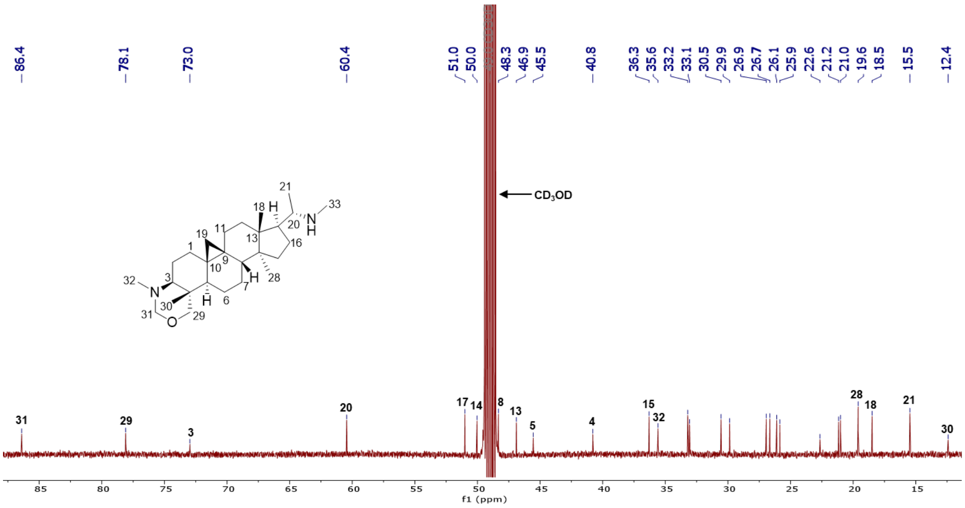

Figure S110. ^{13}C NMR spectrum of *N*20-demethyl deoxycyclobuxoxazine A (**15**) (CD_3OD, 150 MHz).

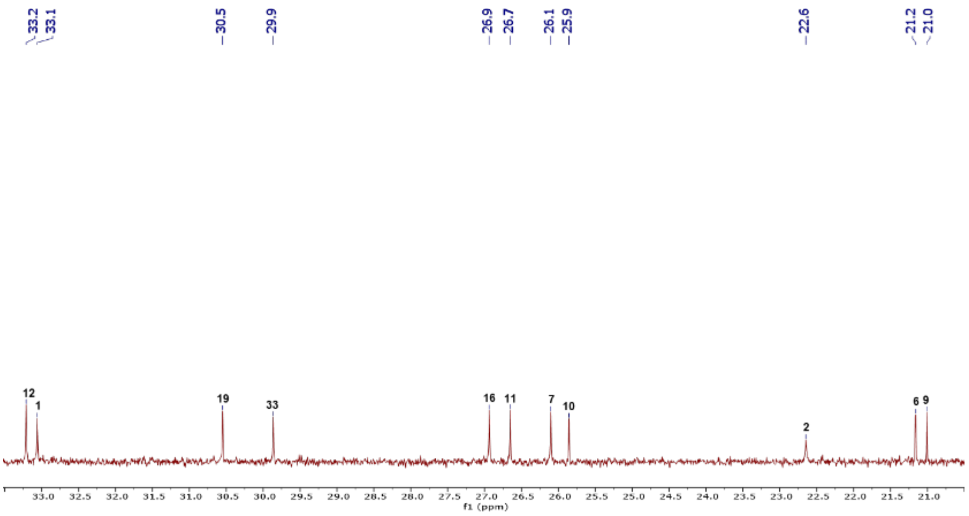

Figure S111. Detail of the ^{13}C NMR spectrum of *N*$_{20}$-demethyl deoxycyclobuxoxazine A (**15**) (CD_3OD, 150 MHz).

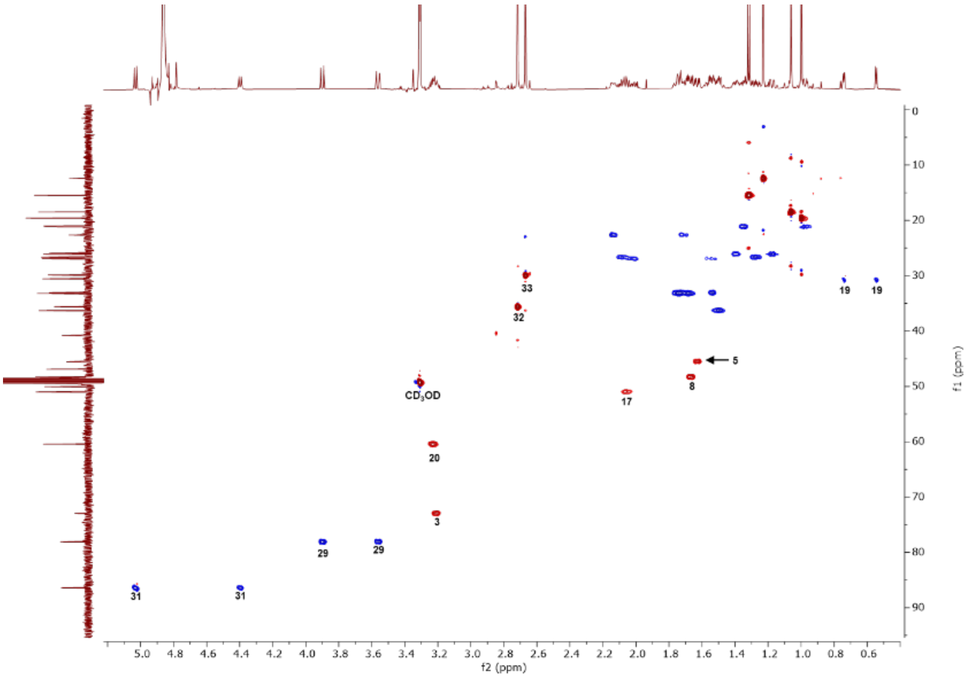

Figure S112. $^{1}H/^{13}C$ HSQC spectrum of *N*$_{20}$-demethyl deoxycyclobuxoxazine A (**15**) (CD_3OD, 600/150 MHz).

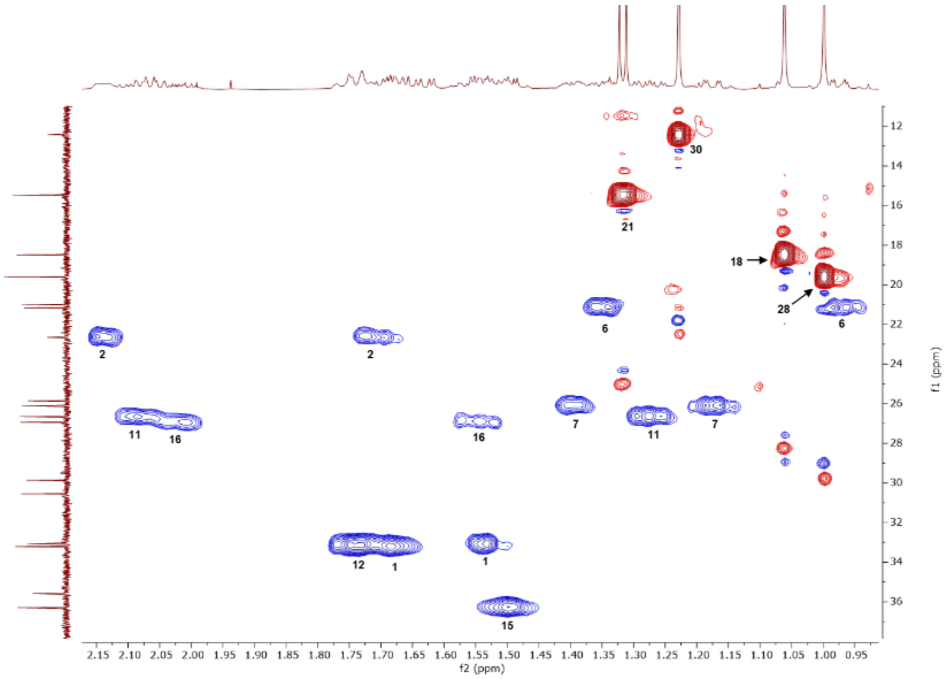

Figure S113. Detail of the $^{1}H/^{13}C$ HSQC spectrum of *N*$_{20}$-demethyl deoxycyclobuxoxazine A (**15**) (CD_3OD, 600/150 MHz).

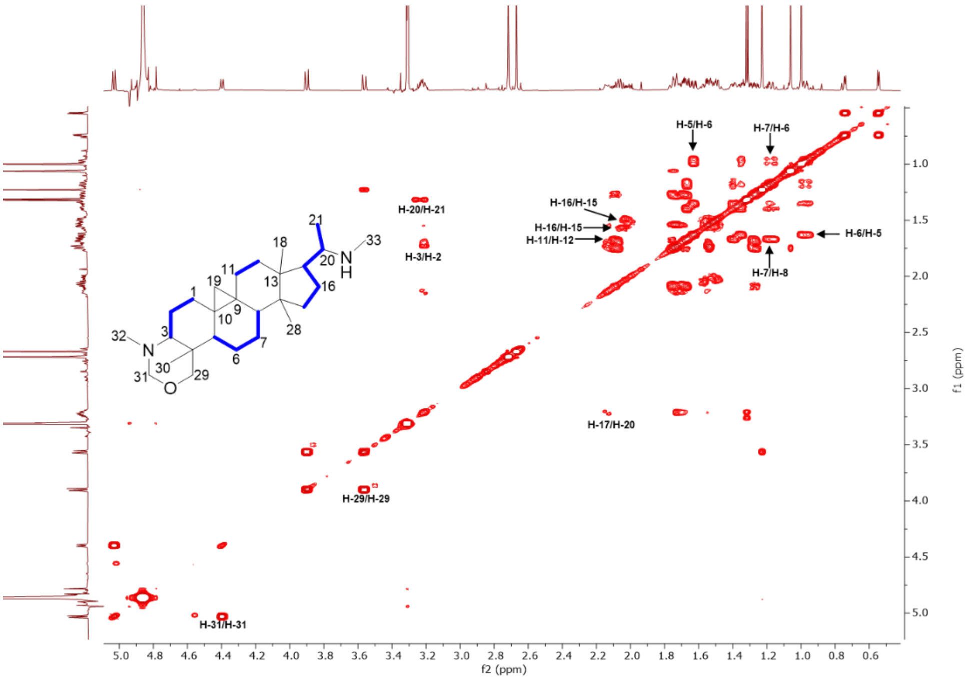

Figure S114. $^{1}H/^{1}H$ COSY spectrum (key correlations) of *N*$_{20}$-demethyl deoxycyclobuxoxazine A (**15**) (CD_3OD, 600 MHz).

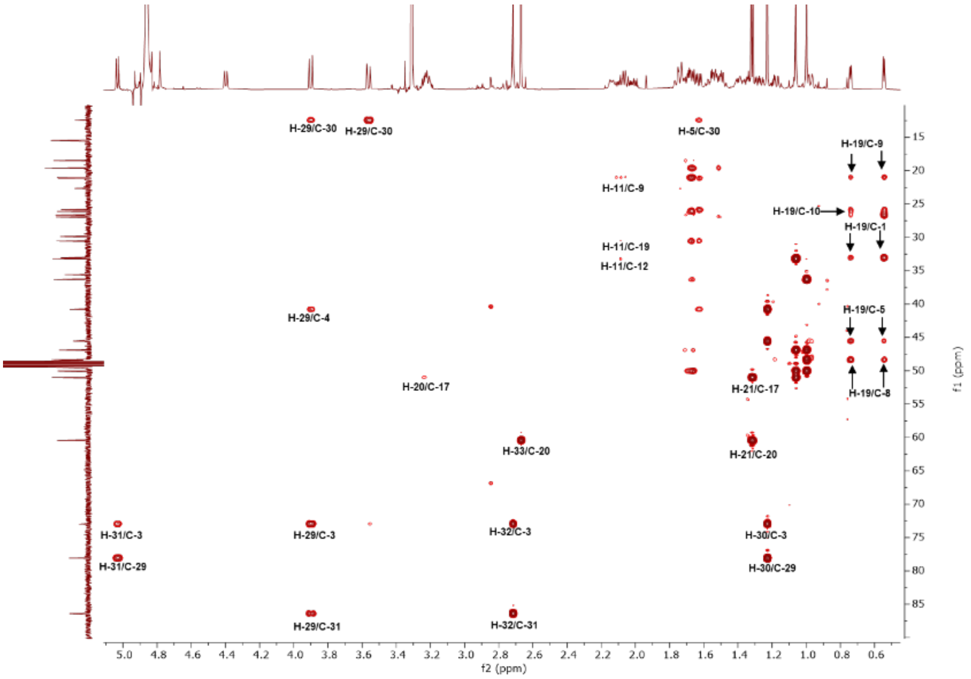

Figure S115. $^{1}H/^{13}C$ HMBC spectrum (key correlations) of N_{20}-demethyl deoxycyclobuxoxazine A (**15**) (CD_3OD, 600/150 MHz).

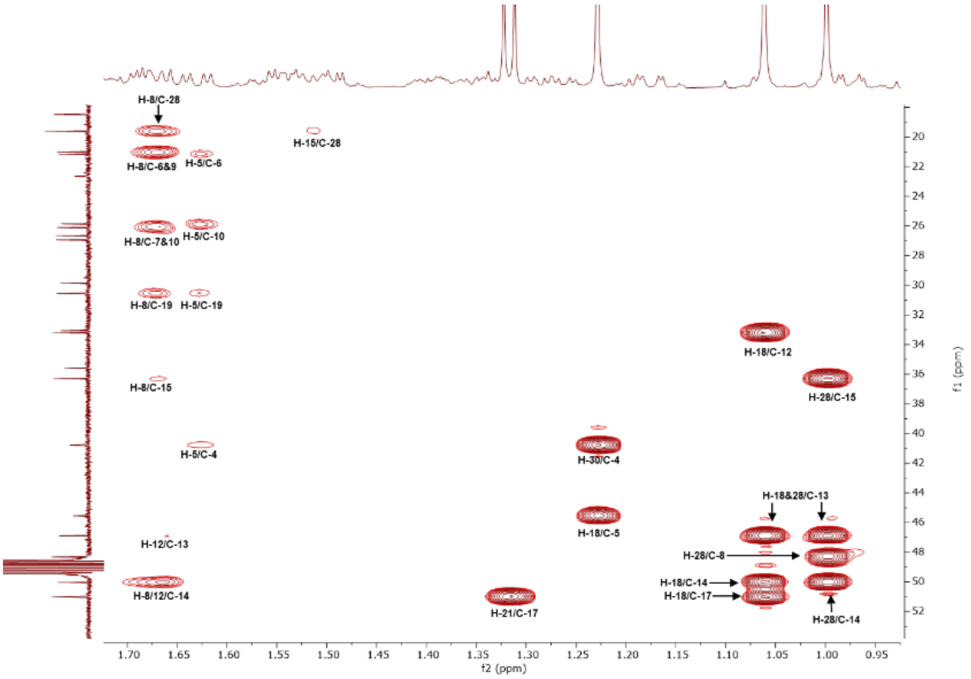

Figure S116. Detail of the $^{1}H/^{13}C$ HMBC spectrum (key correlations) of N_{20}-demethyl deoxycyclobuxoxazine A (**15**) (CD_3OD, 600/150 MHz).

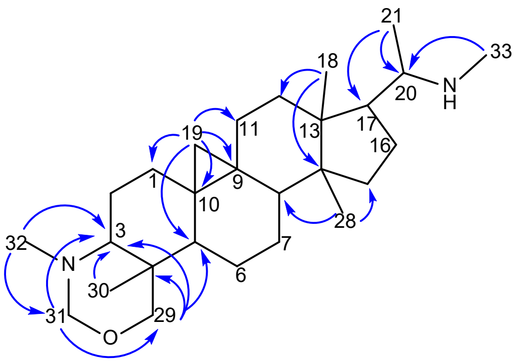

Figure S117. Key HMBC (blue arrows) correlations of *N*$_{20}$-demethyl deoxycyclobuxoxazine A (**15**).

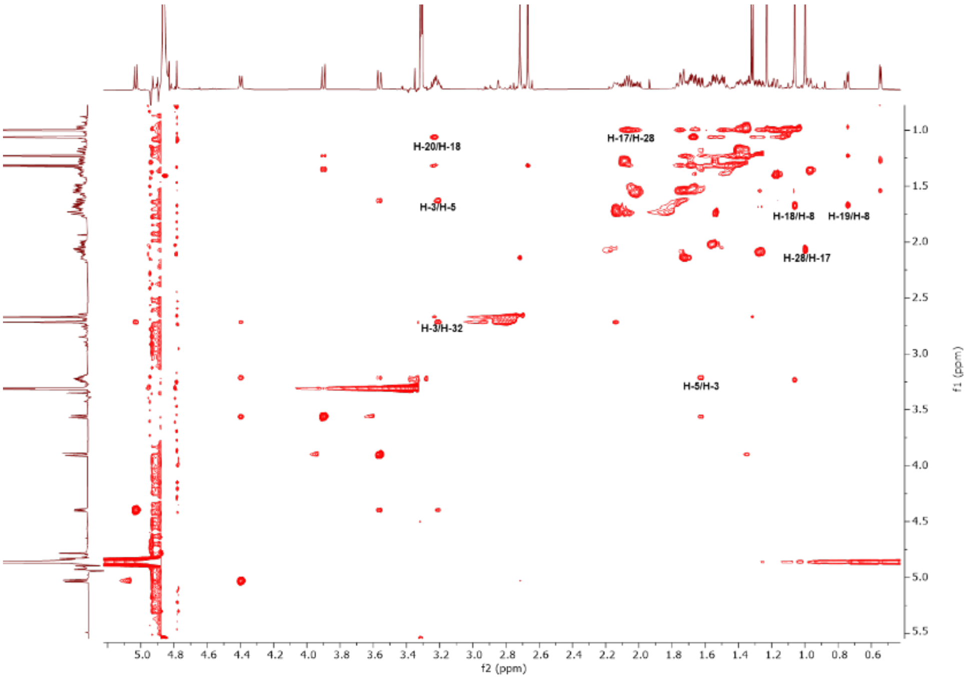

Figure S118. ^{1}H/^{1}H NOESY spectrum (key correlations) of *N*$_{20}$-demethyl deoxycyclobuxoxazine A (**15**) (CD_3OD, 600 MHz).

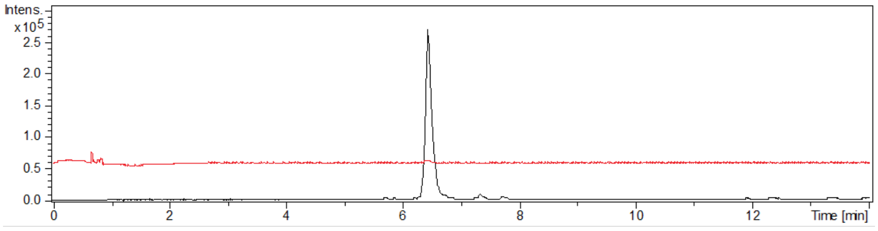

Figure S119. UHPLC/+ESI-QqTOF-MS/MS chromatogram of obtusibuxeine A (**16**). Base peak chromatogram 200.0000–1000.0000 +All MS (black); UV chromatogram 200-400 nm (red).

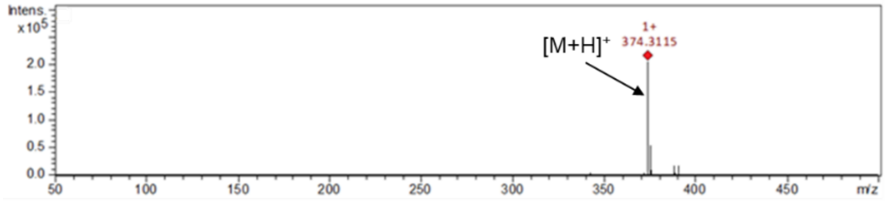

Figure S120. +ESI-QqTOF-MS spectrum of obtusibuxeine A (**16**); *m/z* 374.3115 [M+H]$^+$.

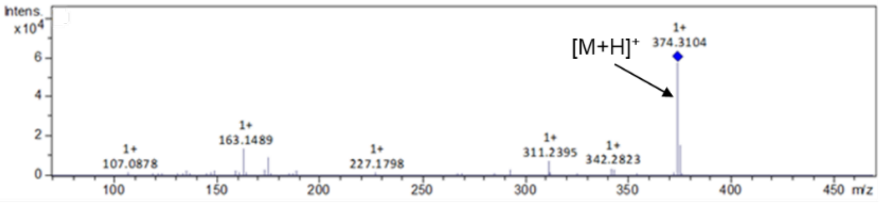

Figure S121. +ESI-QqTOF MS/MS spectrum of obtusibuxeine A (**16**).

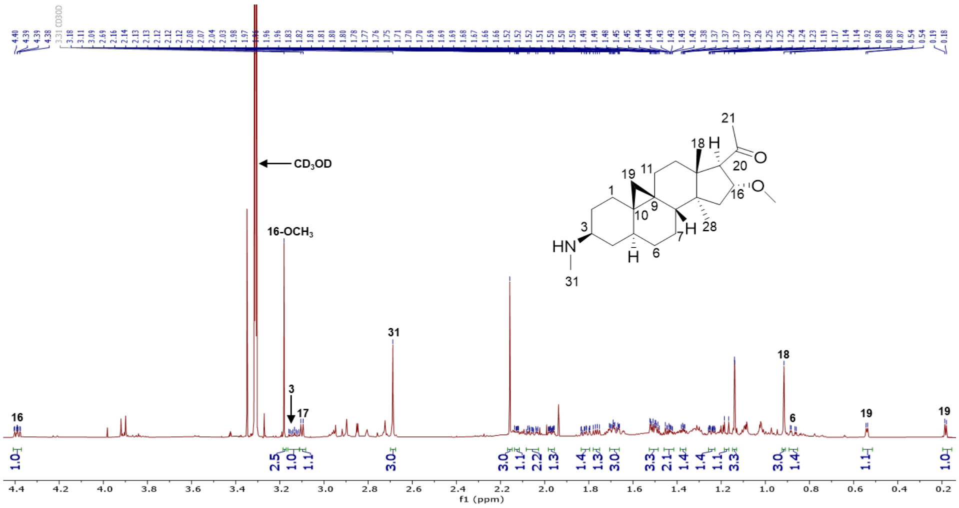

Figure S122. ^{1}H NMR spectrum of obtusibuxeine A (**16**) (CD_3OD, 600 MHz).

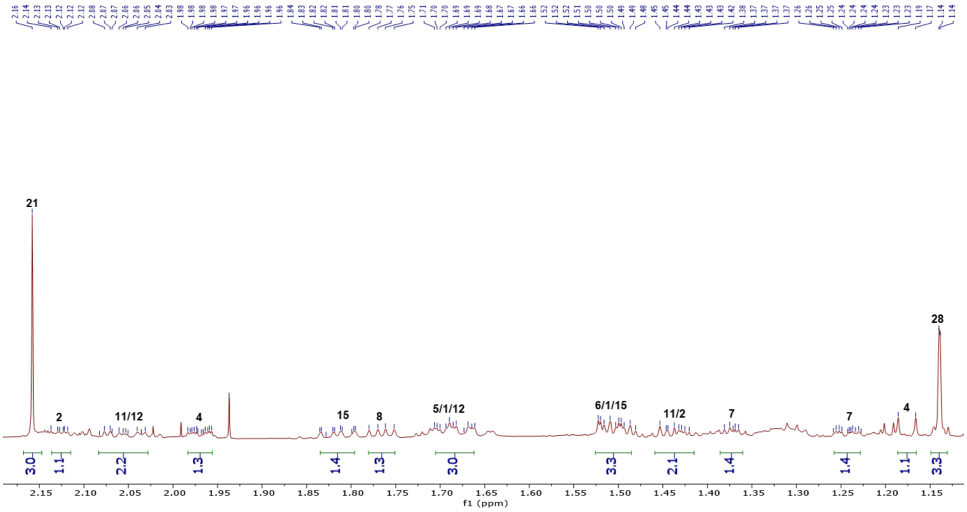

Figure S123. Detail of the ^{1}H NMR spectrum of obtusibuxeine A (**16**) (CD_3OD, 600 MHz).

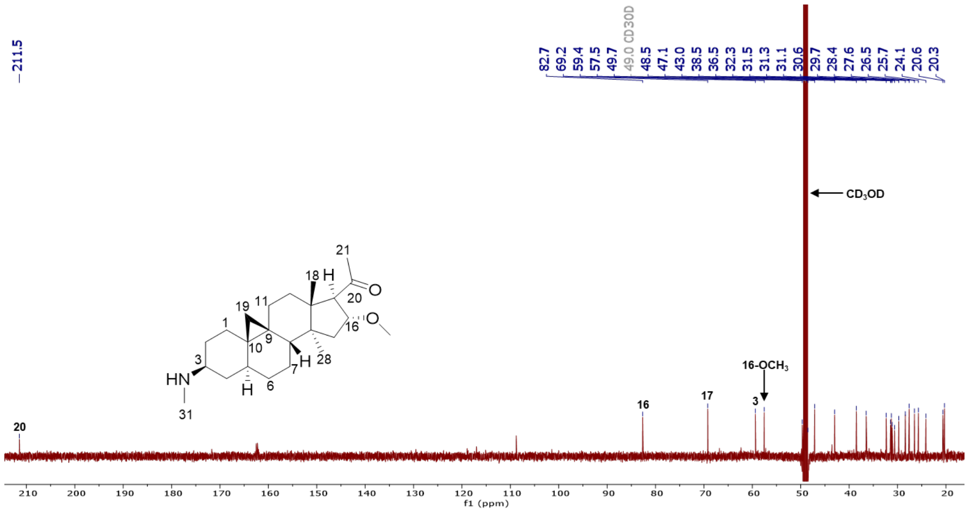

Figure S124. ^{13}C NMR spectrum of obtusibuxeine A (**16**) (CD_3OD, 150 MHz).

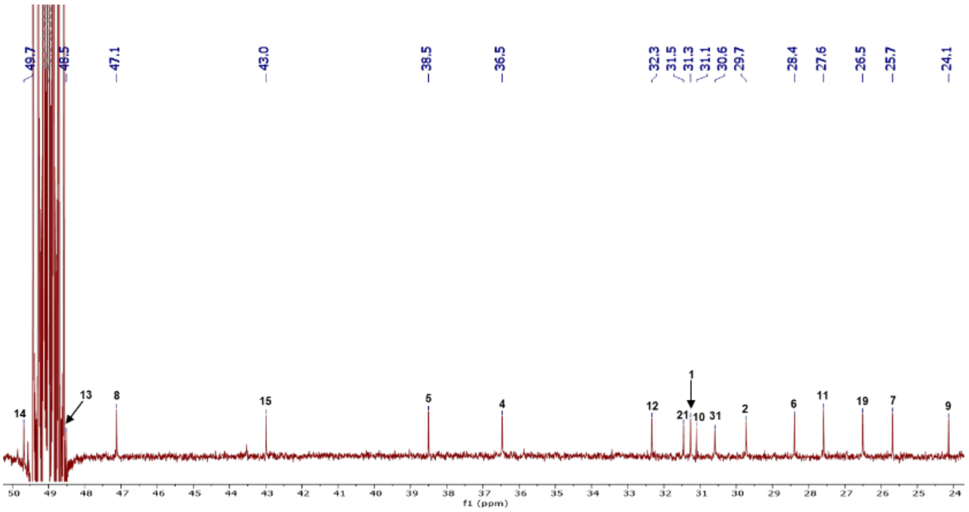

Figure S125. Detail of the ^{13}C NMR spectrum of obtusibuxeine A (**16**) (CD_3OD, 150 MHz).

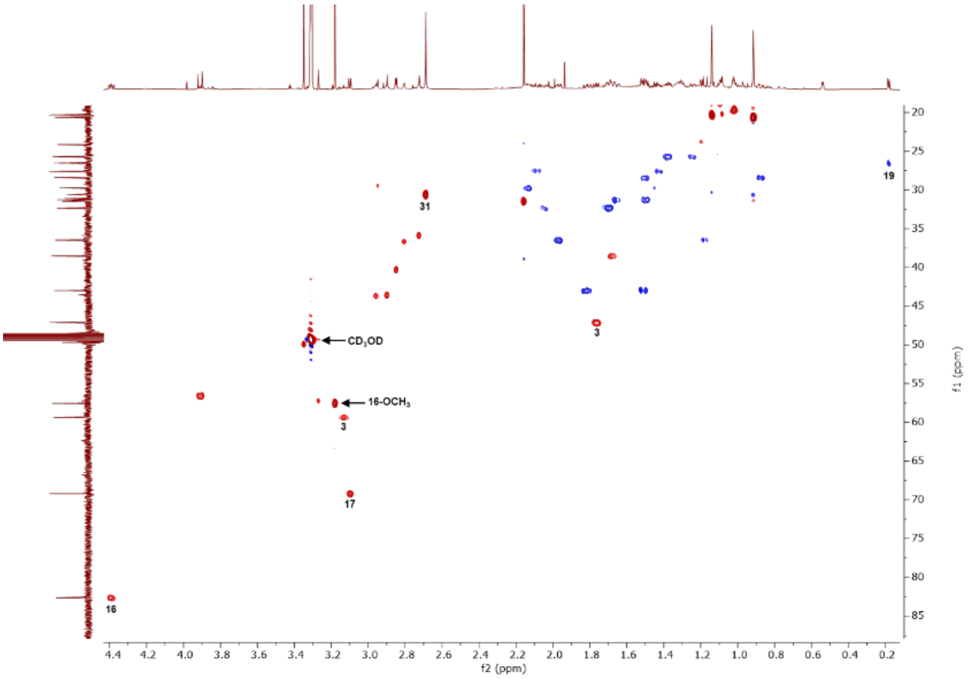

Figure S126. $^{1}H/^{13}C$ HSQC spectrum of obtusibuxeine A (**16**) (CD_3OD, 600/150 MHz).

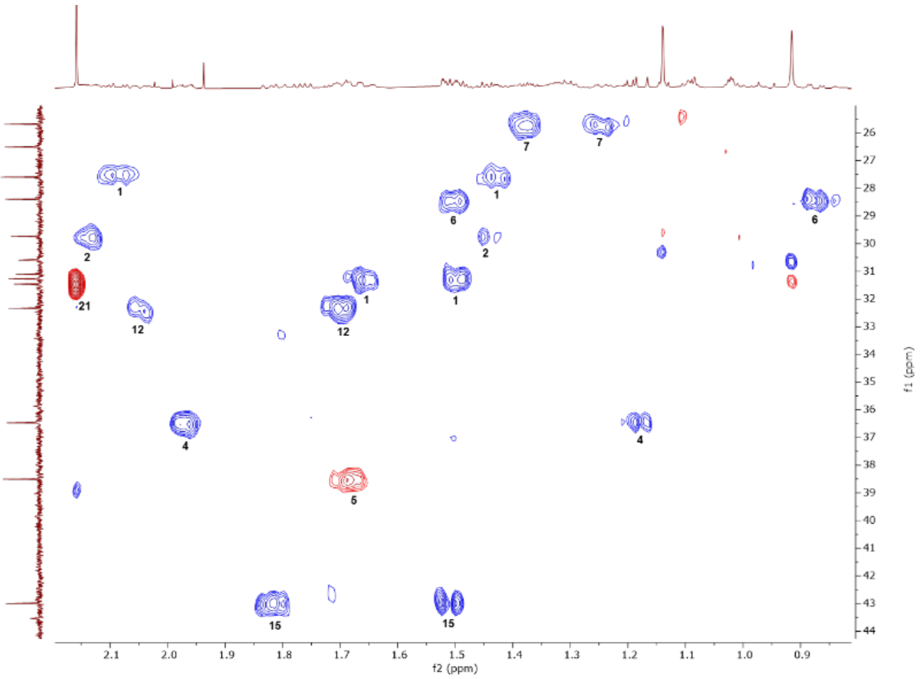

Figure S127. Detail of the ^{1}H/^{13}C HSQC spectrum of obtusibuxeine A (**16**) (CD_3OD, 600/150 MHz).

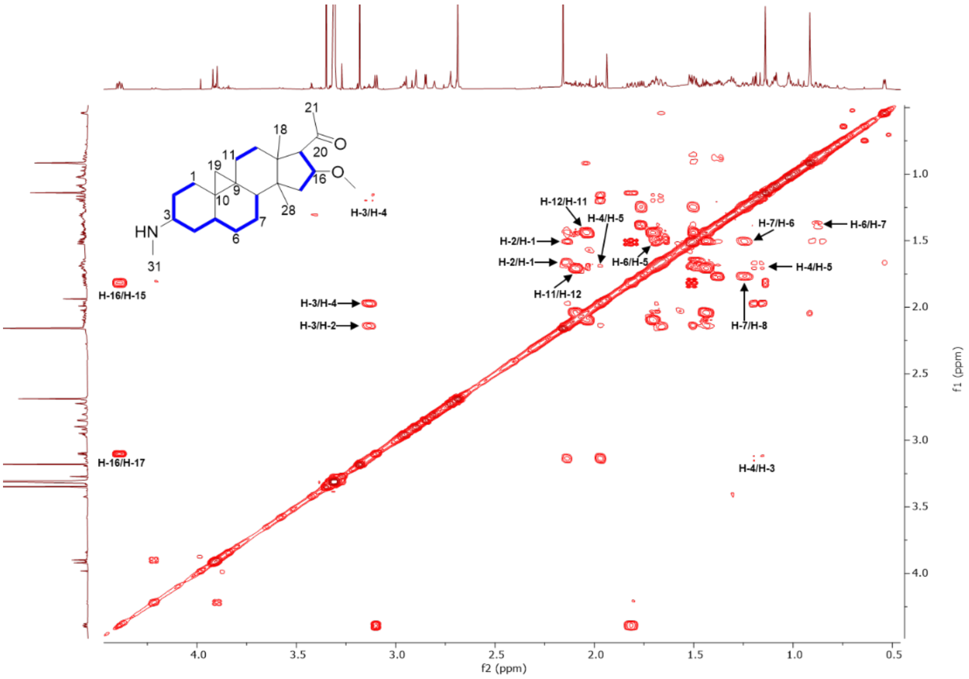

Figure S128. ^{1}H/^{1}H COSY spectrum (key correlations) of obtusibuxeine A (**16**) (CD_3OD, 600 MHz).

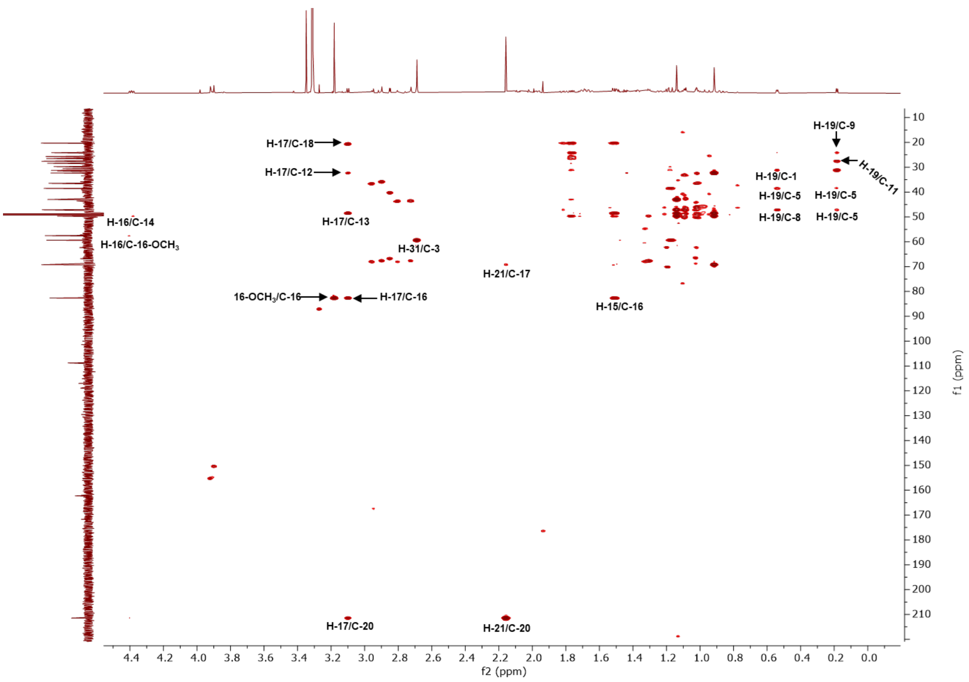

Figure S129. ^{1}H/^{13}C HMBC spectrum (key correlations) of obtusibuxeine A (**16**) (CD_3OD, 600/150 MHz).

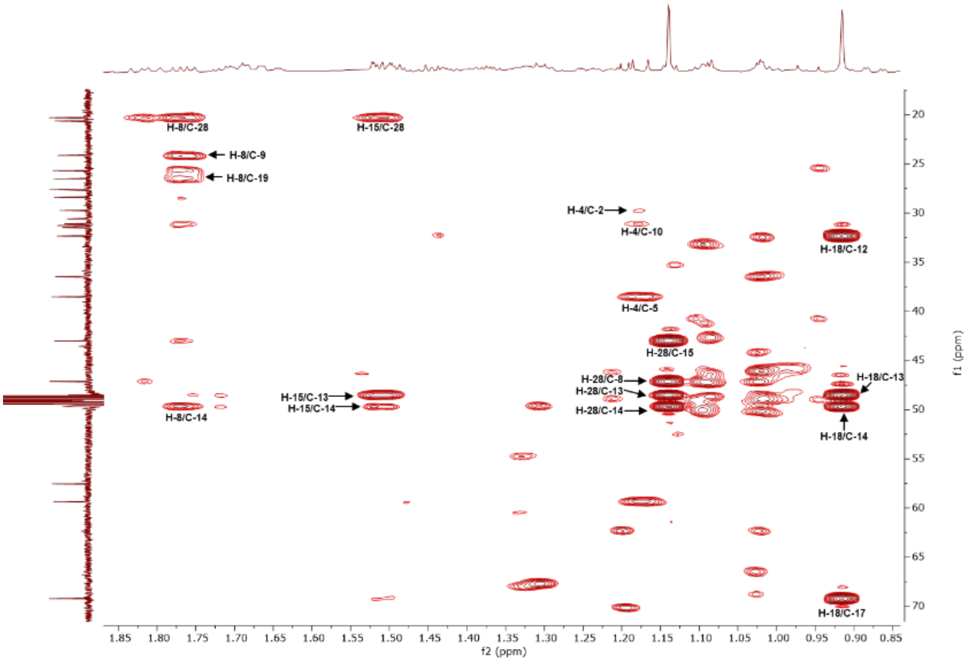

Figure S130. Detail of the ^{1}H/^{13}C HMBC spectrum (key correlations) of obtusibuxeine A (**16**) (CD_3OD, 600/150 MHz).

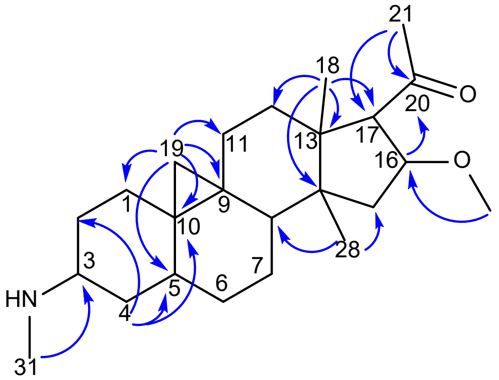

Figure S131. Key HMBC (blue arrows) correlations of obtusibuxeine A (**16**).

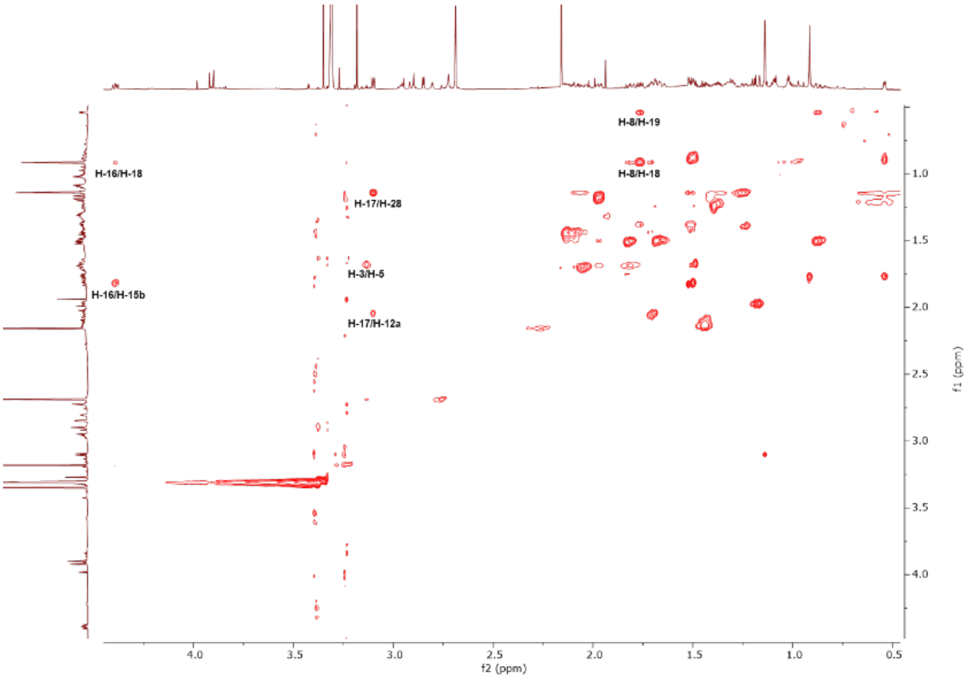

Figure S132. ^{1}H/^{1}H NOESY spectrum (key correlations) of obtusibuxeine A (**16**) (CD_3OD, 600 MHz).

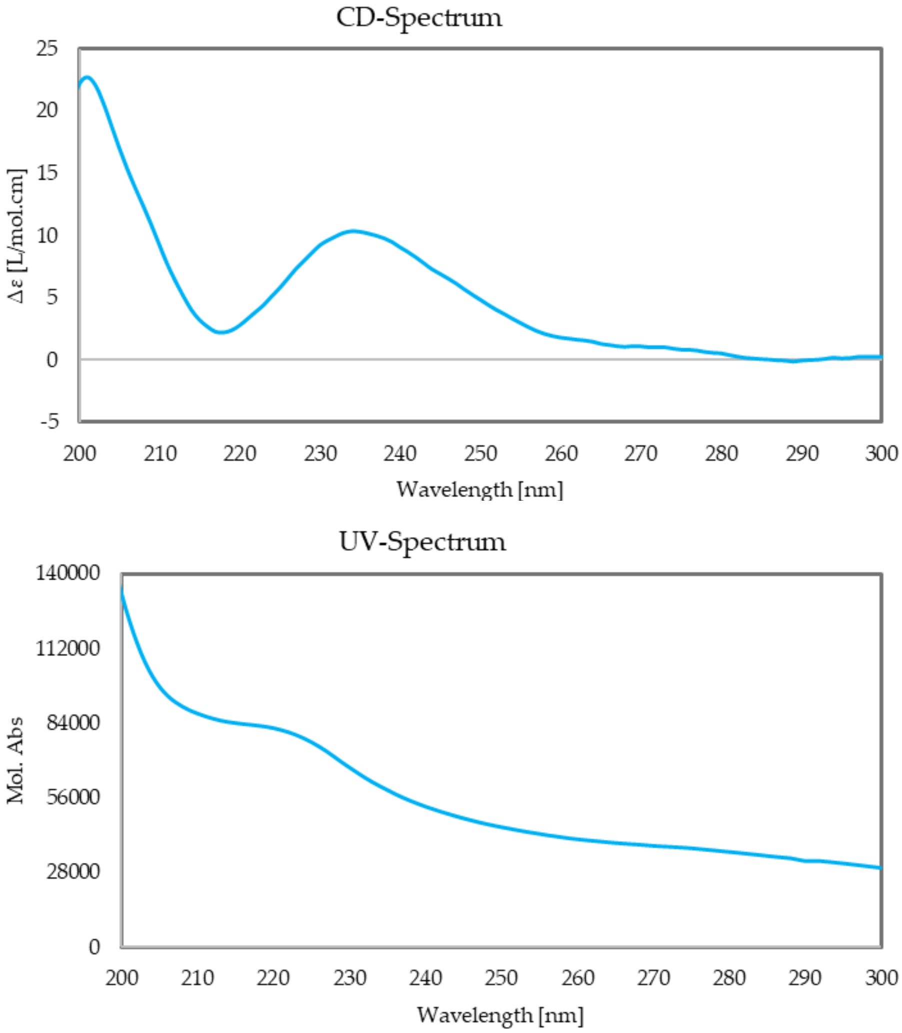

Figure S133. CD and UV spectra of O[10]*-obtusifuranamine-A (***17***) in MeOH (0.1 mg/mL).* CD (λ[nm], Δε): 270, ≈1 (sh); 234, 10.4; 218, 2.2; 202, 22.7; 200, 22.1 (last reading); UV (λ[nm], log ε): 222, 4.91 (sh); 200, 5.12 (last reading).

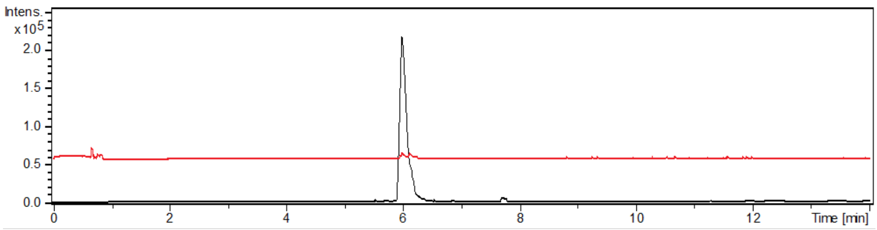

Figure S134. UHPLC/+ESI-QqTOF-MS/MS chromatogram of *O*[10]-obtusifuranamine-A (**17**). Base peak chromatogram 200.0000–1000.0000 +All MS (black); UV chromatogram 200-400 nm (red).

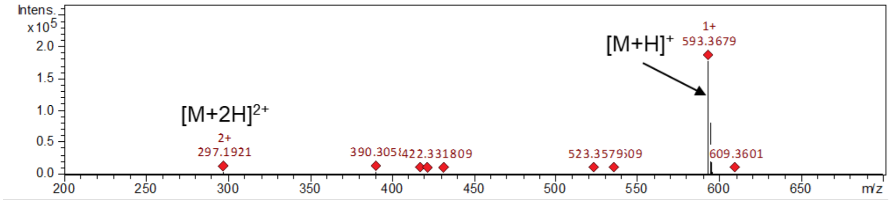

Figure S135. +ESI-QqTOF-MS spectrum of *O*[10]-obtusifuranamine-A (**17**); *m/z* 593.3679 $[M+H]^+$, 297.1921 $[M+2H]^{2+}$.

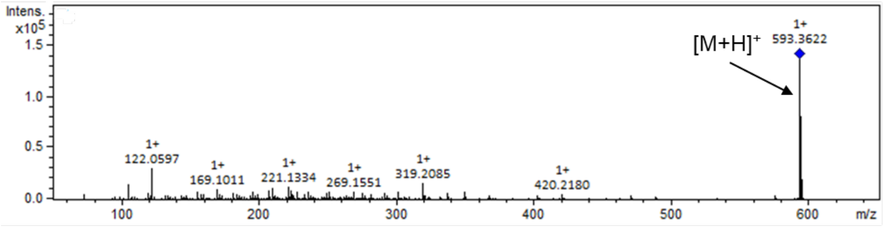

Figure S136. +ESI-QqTOF MS/MS spectrum of *O*[10]-obtusifuranamine-A (**17**).

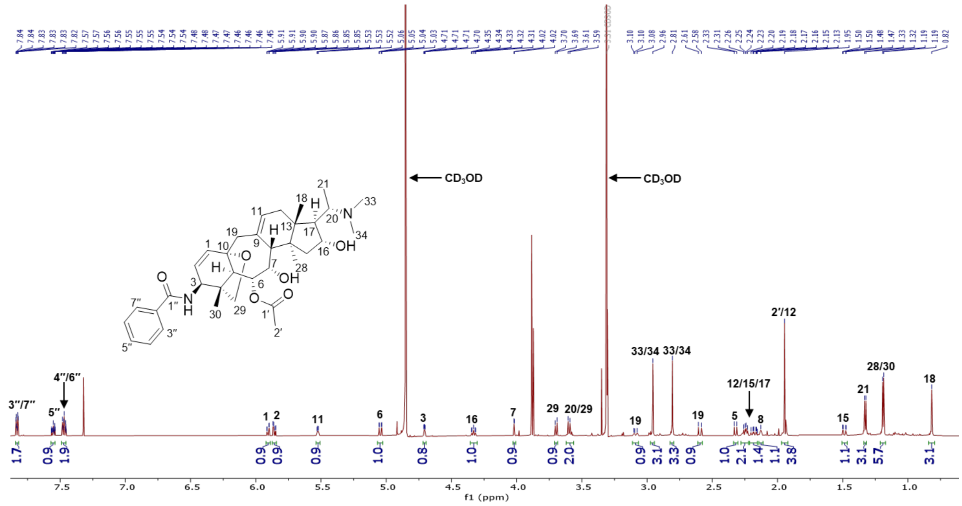

Figure S137. ^{1}H NMR spectrum of *O*[10]-obtusifuranamine-A (**17**) (CD_3OD, 600 MHz).

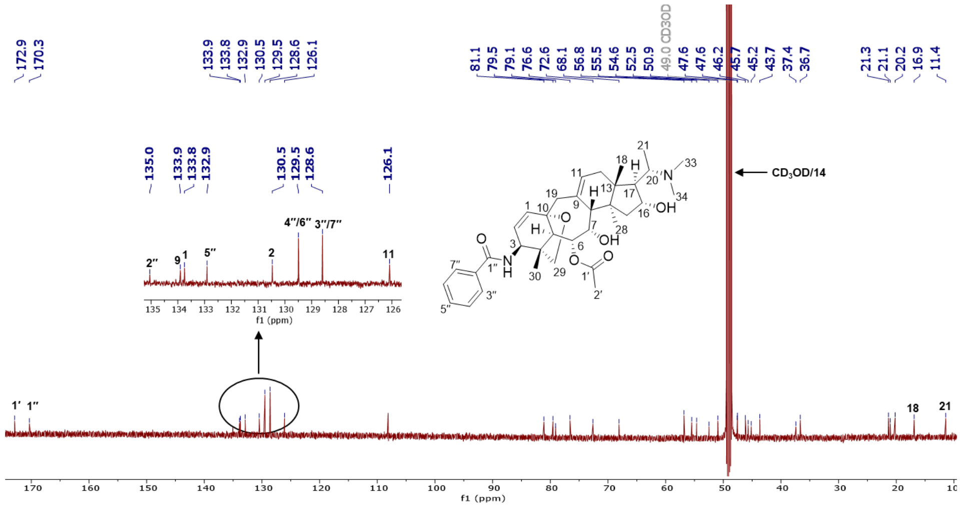

Figure S138. ^{13}C NMR spectrum of *O*[10]-obtusifuranamine-A (**17**) (CD_3OD, 150 MHz).

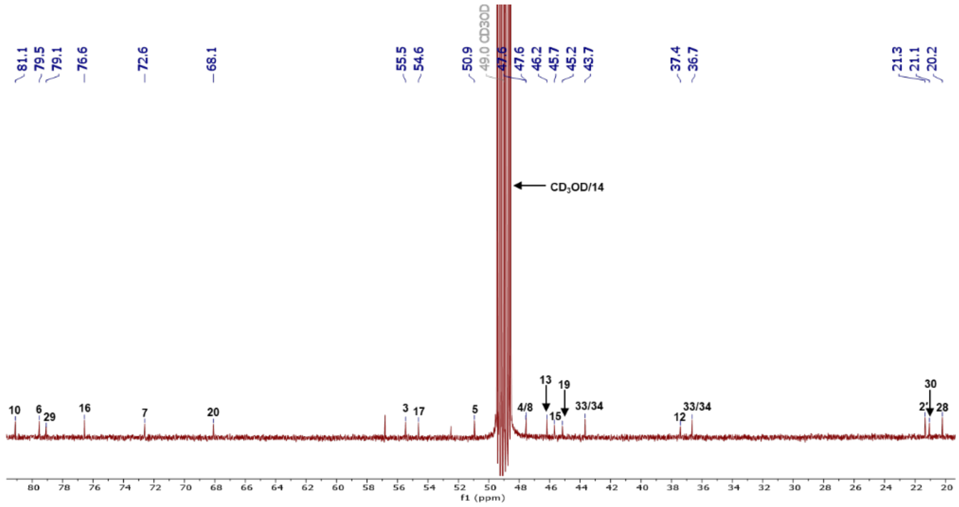

Figure S139. Detail of the ^{13}C NMR spectrum of *O*[10]-obtusifuranamine-A (**17**) (CD_3OD, 150 MHz).

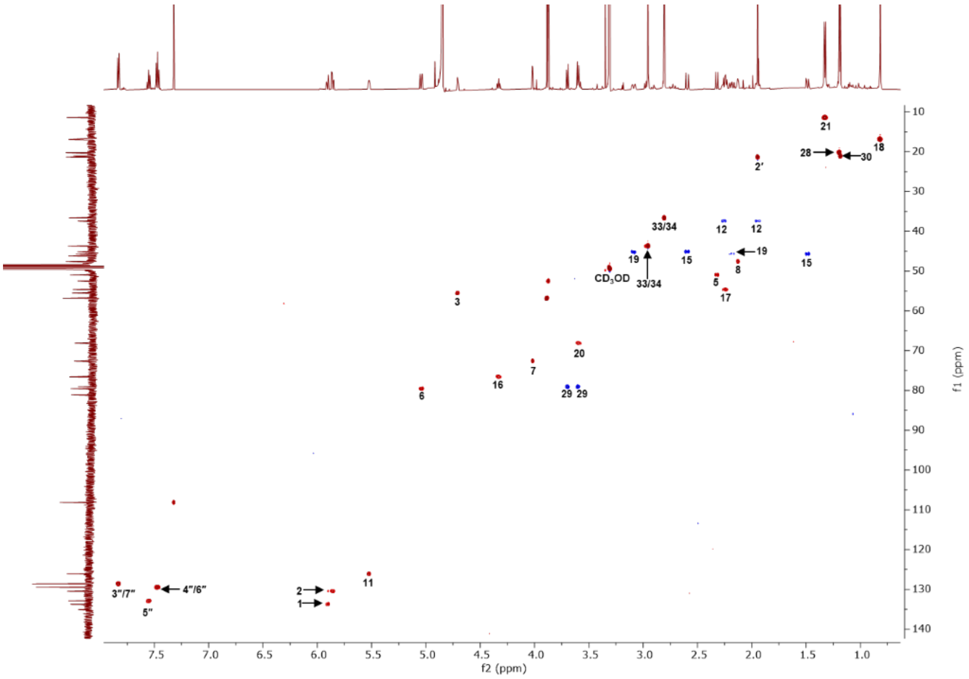

Figure S140. $^{1}H/^{13}C$ HSQC spectrum of *O*[10]-obtusifuranamine-A (**17**) (CD_3OD, 600/150 MHz).

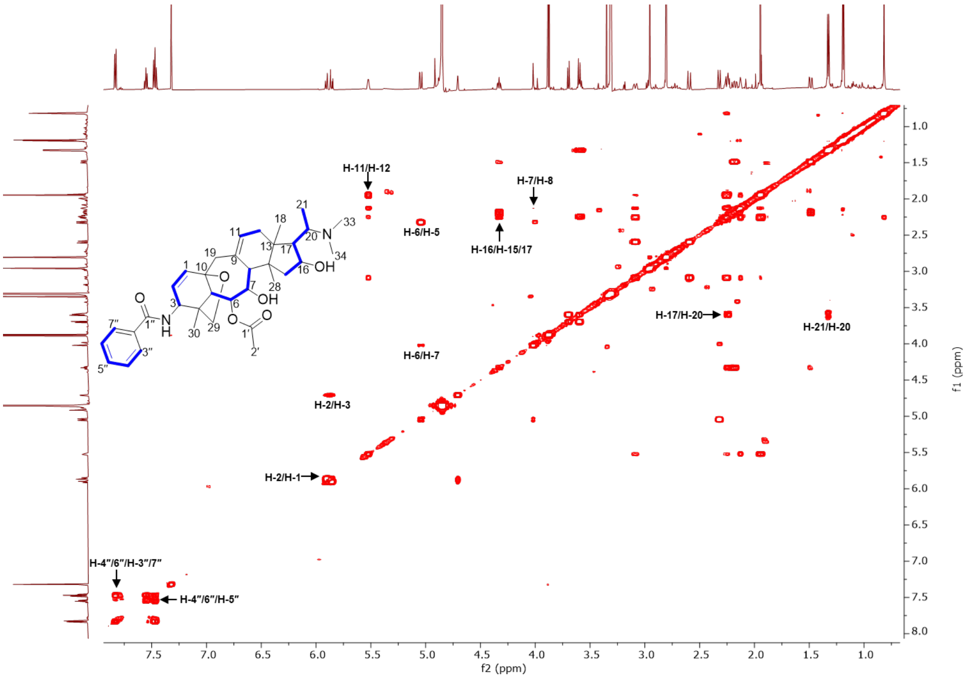

Figure S141. ^{1}H/^{1}H COSY spectrum (key correlations) of *O*[10]-obtusifuranamine-A (**17**) (CD_3OD, 600 MHz).

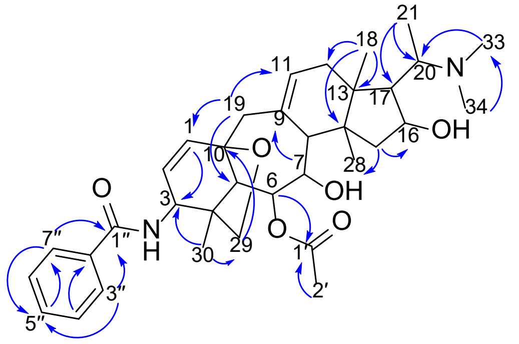

Figure S142. Key HMBC (blue arrows) correlations of *O*[10]-obtusifuranamine-A (**17**).

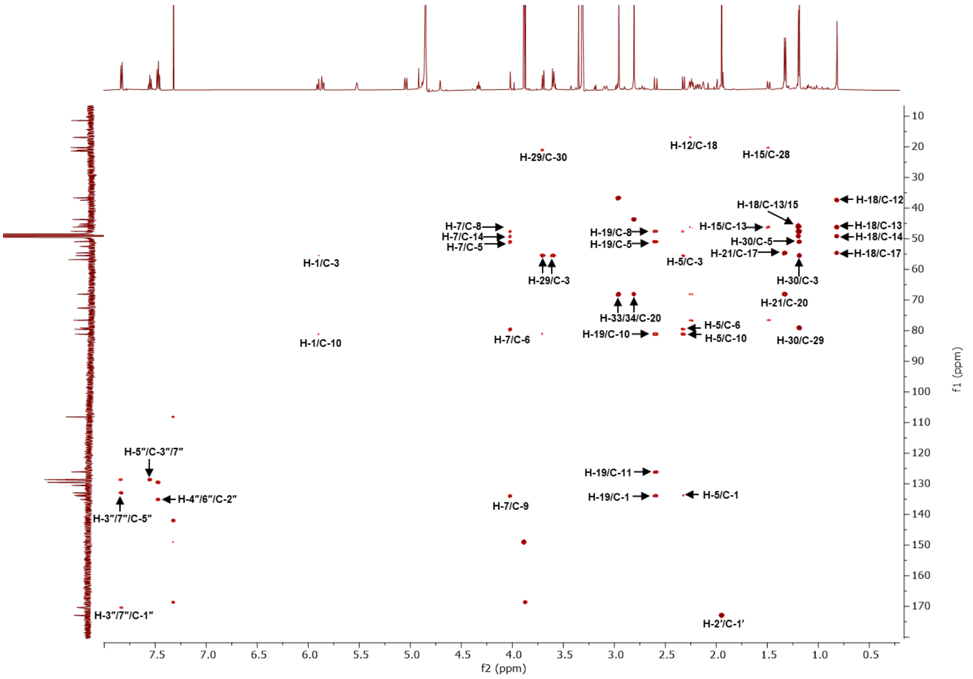

Figure S143. $^{1}H/^{13}C$ HMBC spectrum (key correlations) of *O*[10]-obtusifuranamine-A (**17**) (CD_3OD, 600/150 MHz).

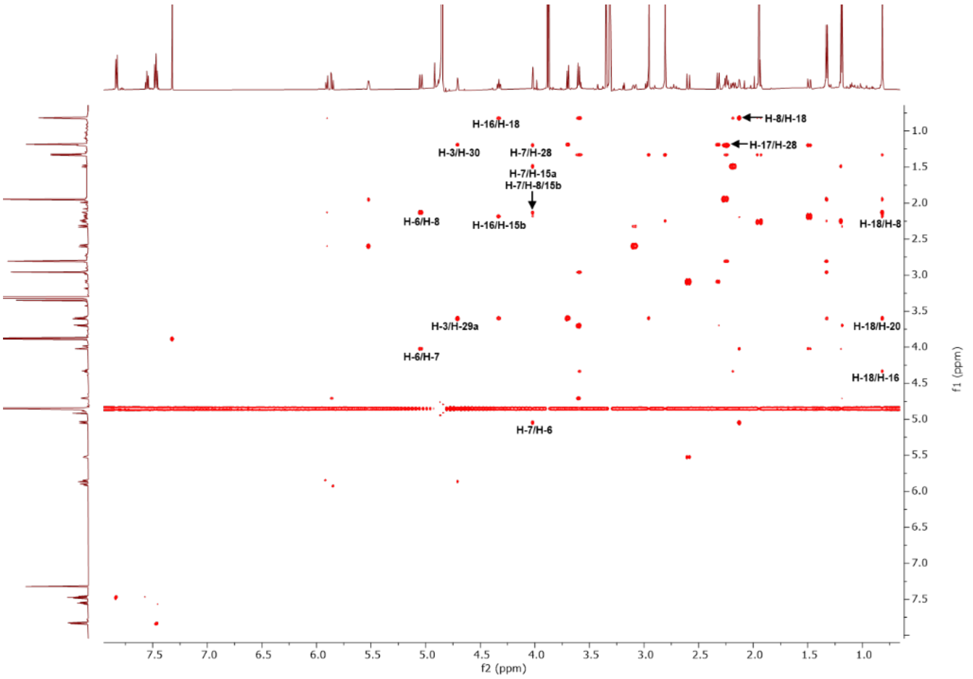

Figure S144. $^{1}H/^{1}H$ NOESY spectrum (key correlations) of *O*[10]-obtusifuranamine-A (**17**) (CD_3OD, 600 MHz).

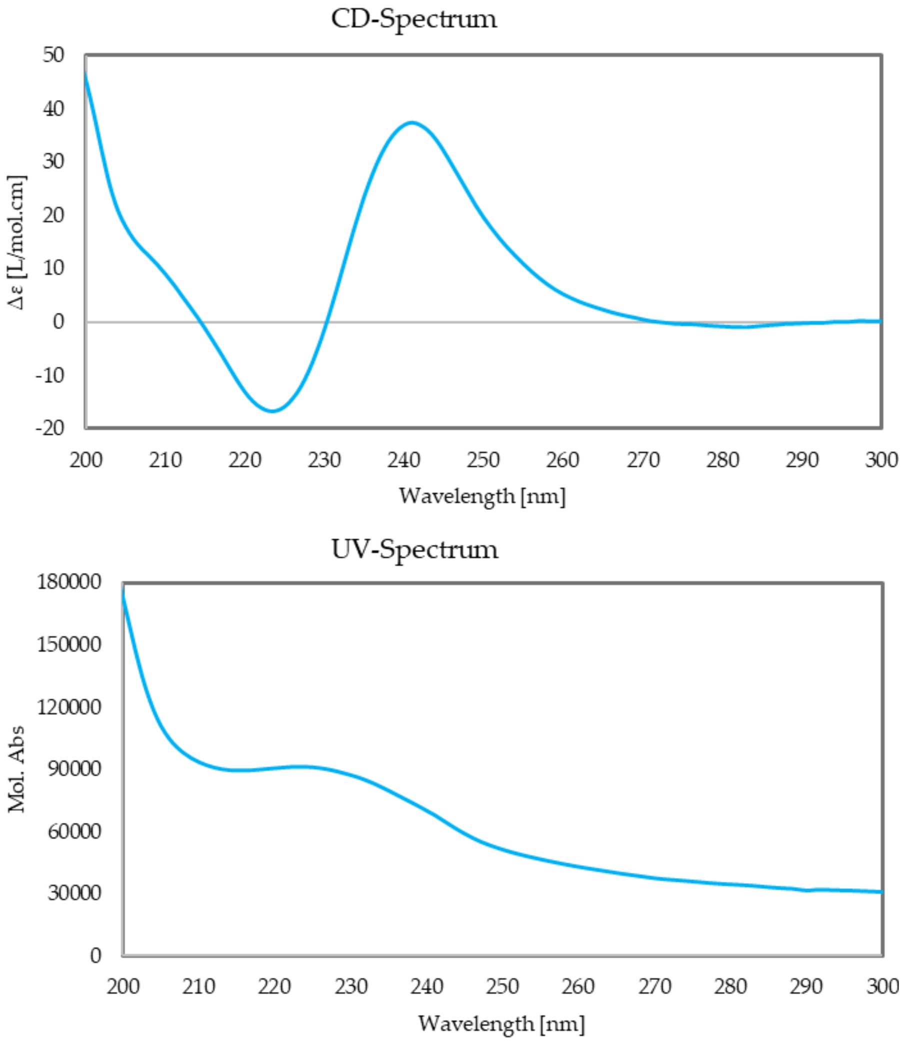

Figure S145. CD and UV spectra of *O*[10]-obtusifuranamine-B (**18**) in MeOH (0.1 mg/mL). CD (λ[nm], Δε): 241, 37.3; 223, -16.8; 210, 8.9 (sh), 200, 45.3 (last reading); UV (λ[nm], log ε): 223, 4.96; 200, 5.24 (last reading).

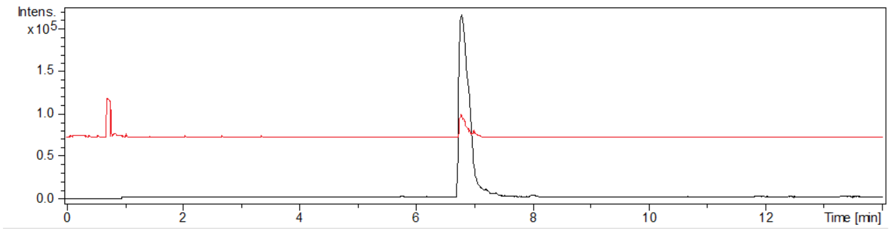

Figure S146. UHPLC/+ESI-QqTOF-MS/MS chromatogram of *O*[10]-obtusifuranamine-B (**18**). Base peak chromatogram 200.0000–1000.0000 +All MS (black); UV chromatogram 200-400 nm (red).

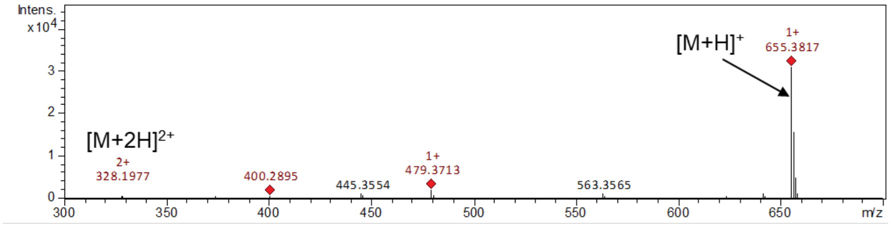

Figure S147. +ESI-QqTOF-MS spectrum of *O*[10]-obtusifuranamine-B (**18**); *m*/*z* 655.3817 $[M+H]^+$, 328.1977 $[M+2H]^{2+}$.

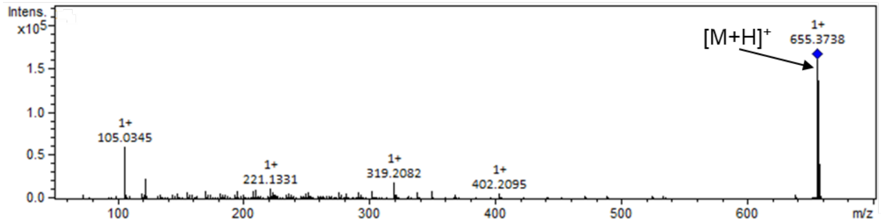

Figure S148. +ESI-QqTOF MS/MS spectrum of *O*[10]-obtusifuranamine-B (**18**).

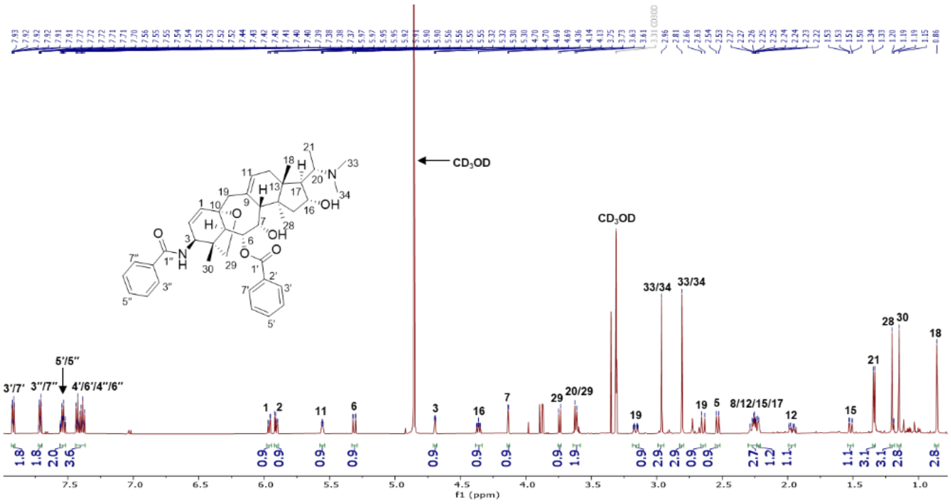

Figure S149. ^{1}H NMR spectrum of *O*[10]-obtusifuranamine-B (**18**) (CD_3OD, 600 MHz).

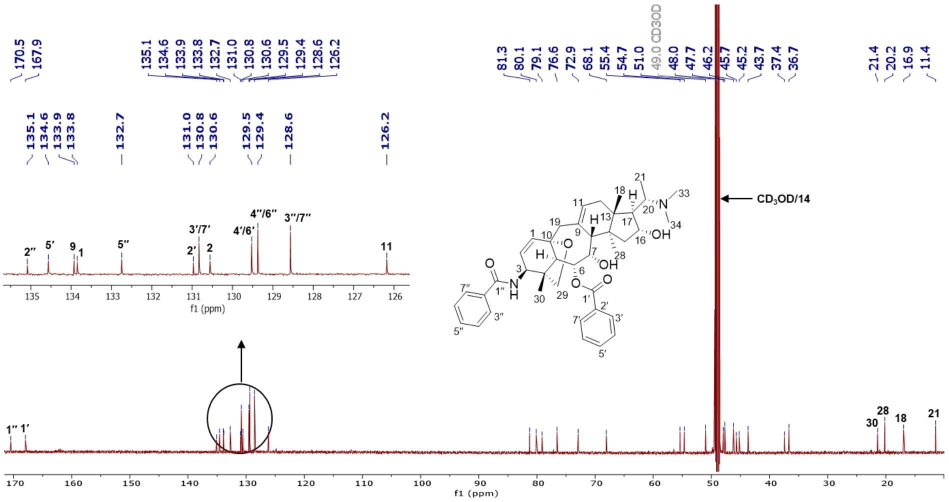

Figure S150. ^{13}C NMR spectrum of *O*[10]-obtusifuranamine-B (**18**) (CD_3OD, 150 MHz).

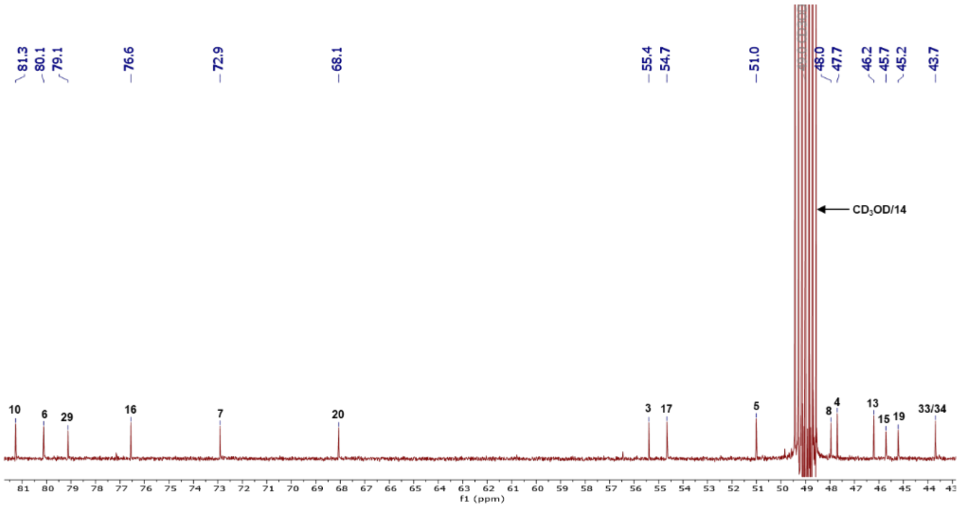

Figure S151. Detail of the ^{13}C NMR spectrum of *O*[10]-obtusifuranamine-B (**18**) (CD_3OD, 150 MHz).

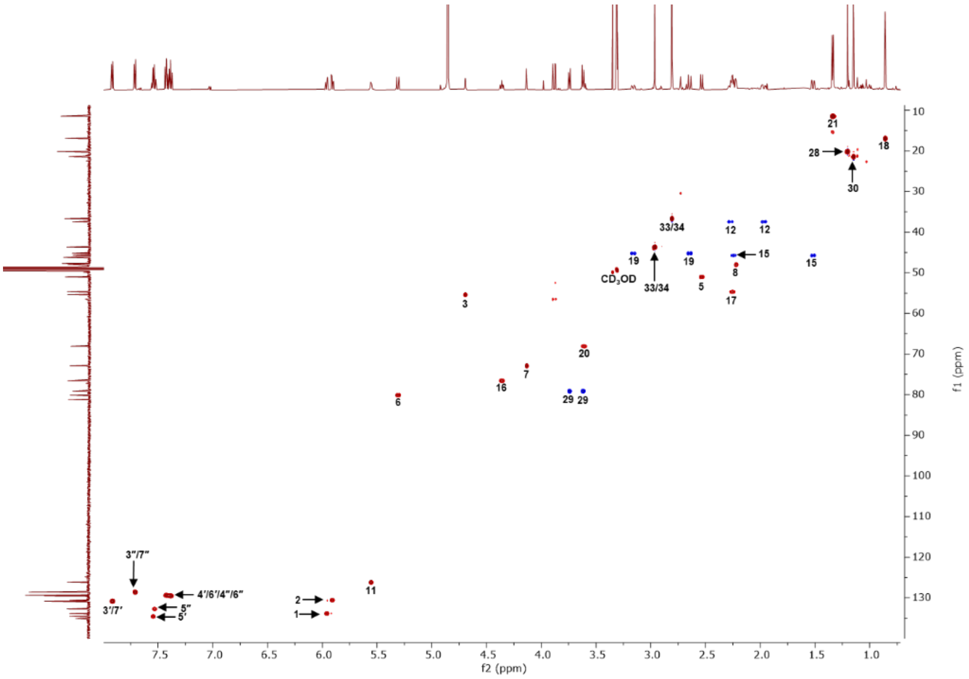

Figure 152. $^{1}H/^{13}C$ HSQC spectrum of *O*[10]-obtusifuranamine-B (**18**) (CD_3OD, 600/150 MHz).

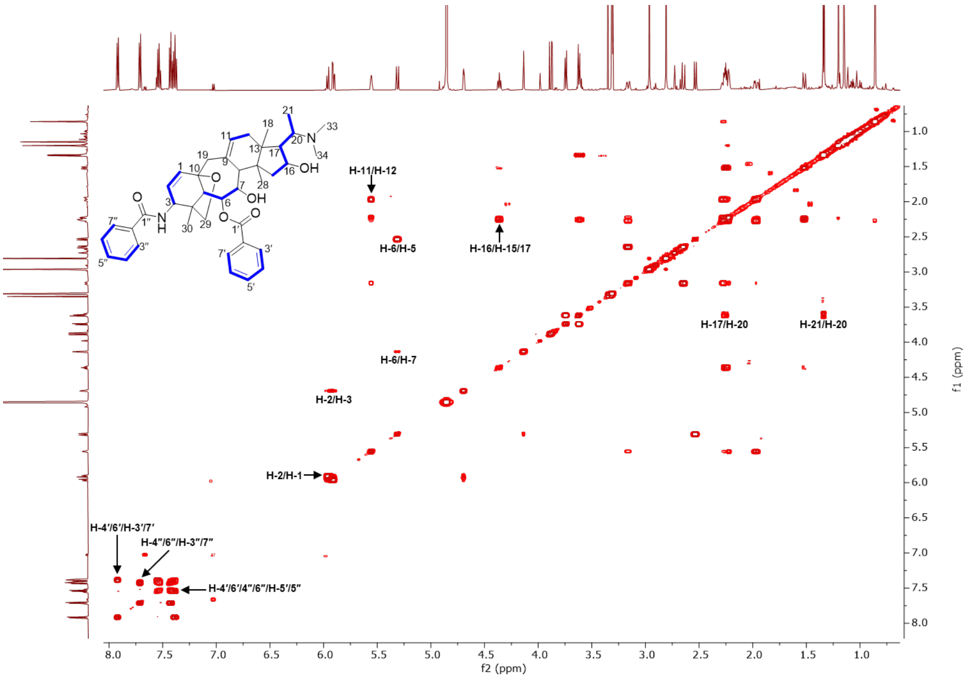

Figure S153. $^{1}H/^{1}H$ COSY spectrum (key correlations) of *O*^{10}-obtusifuranamine-B (**18**) (CD_3OD, 600 MHz).

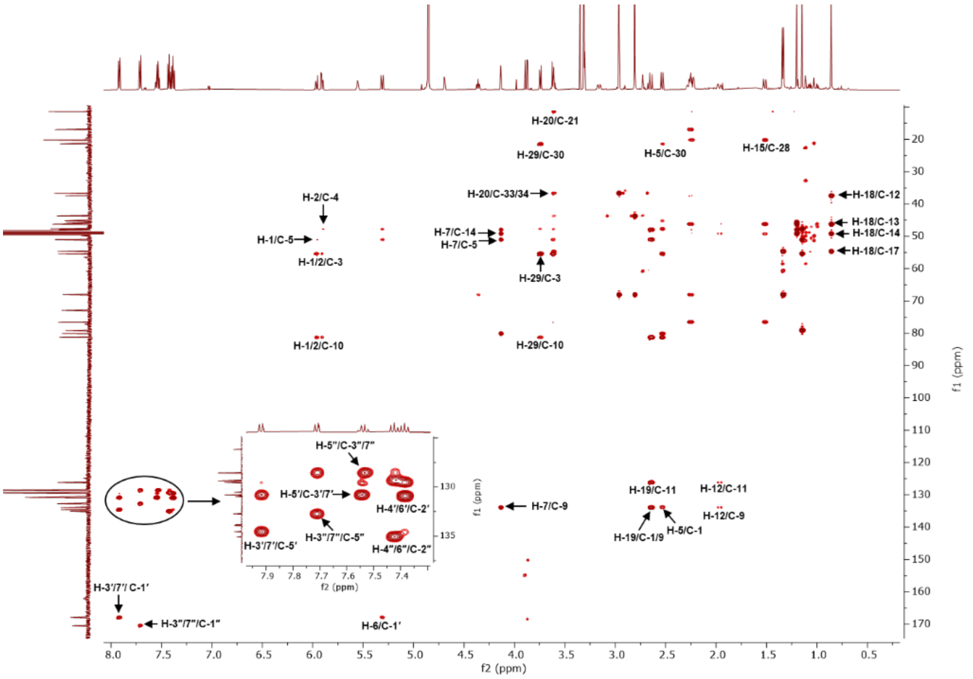

Figure S154. $^{1}H/^{13}C$ HMBC spectrum (key correlations) of *O*^{10}-obtusifuranamine-B (**18**) (CD_3OD, 600/150 MHz).

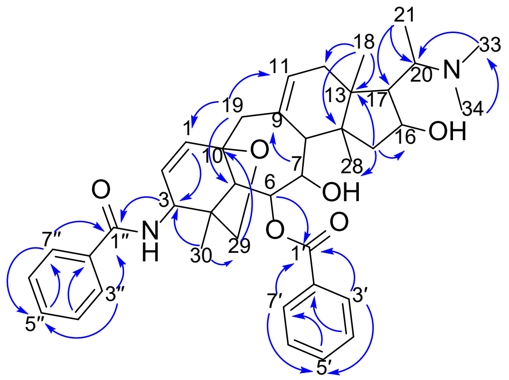

Figure S155. Key HMBC (blue arrows) correlations of *O*[10]-obtusifuranamine-B (**18**).

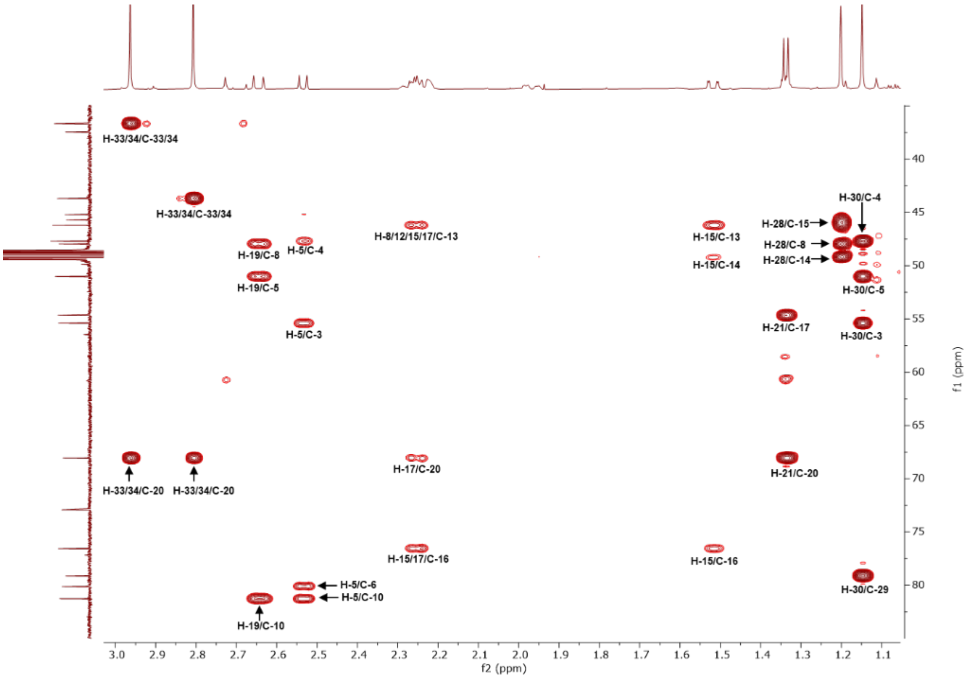

Figure S156. Detail of the $^{1}H/^{13}C$ HMBC spectrum (key correlations) of *O*[10]-obtusifuranamine-B (**18**) (CD_3OD, 600/150 MHz).

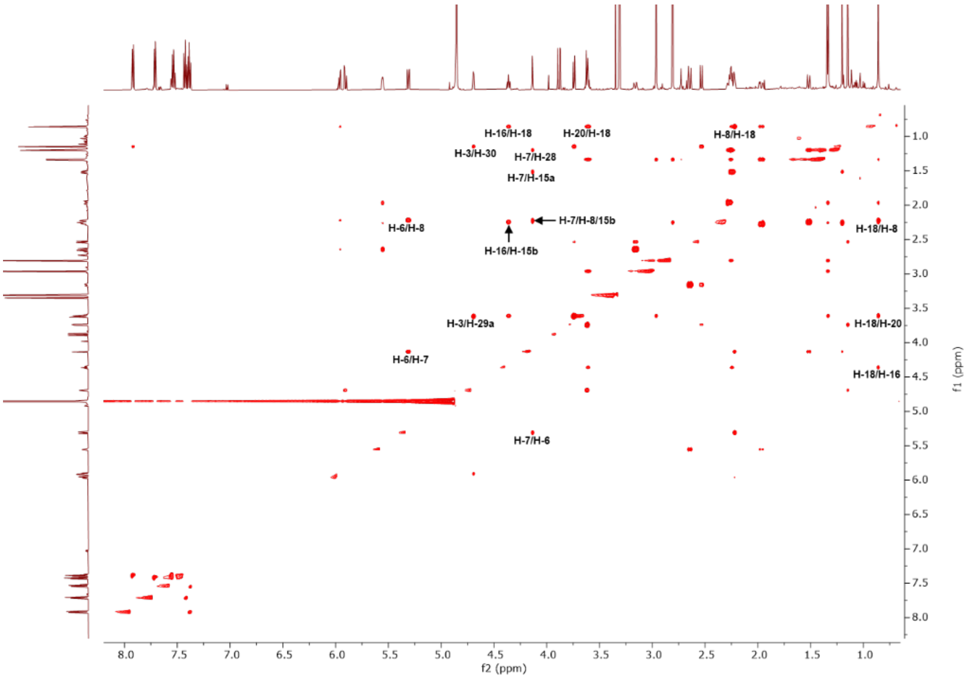

Figure S157. ^{1}H/^{1}H NOESY spectrum (key correlations) of *O*[10]-obtusifuranamine-B (**18**) (CD_3OD, 600 MHz).

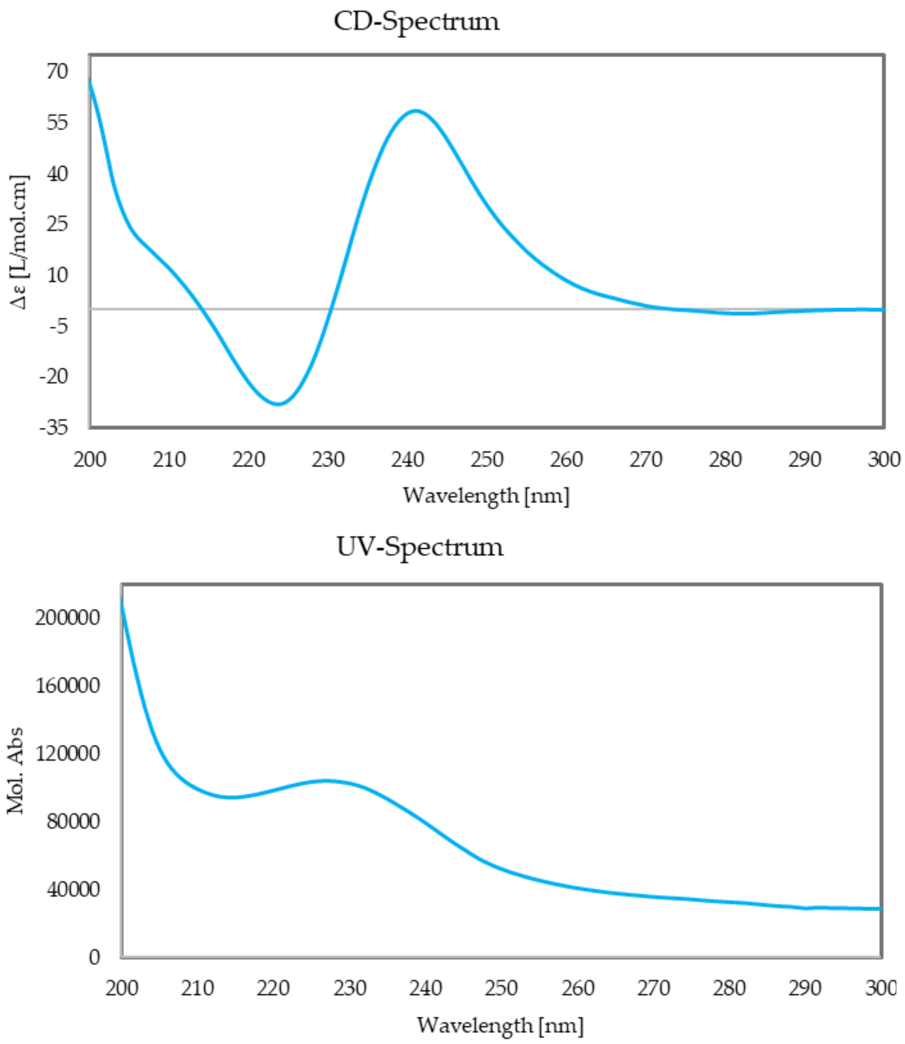

Figure S158. CD and UV spectra of 16-deoxy-*O*[10]-obtusifuranamine-B (**19**) in MeOH (0.1 mg/mL). CD (λ[nm], Δε): 241, 58.6; 224, -27.8; 210, 12 (sh); 200, 66.6 (last reading); UV (λ[nm], log ε): 227, 5.02; 200, 5.31 (last reading).

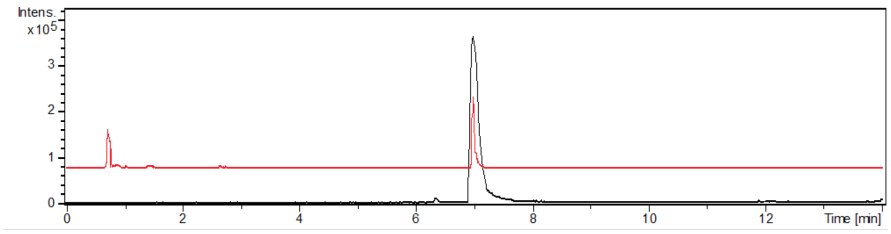

Figure S159. UHPLC/+ESI-QqTOF-MS/MS chromatogram of 16-deoxy-*O*[10]-obtusifuranamine-B (**19**). Base peak chromatogram 200.0000–1000.0000 +All MS (black); UV chromatogram 200-400 nm (red).

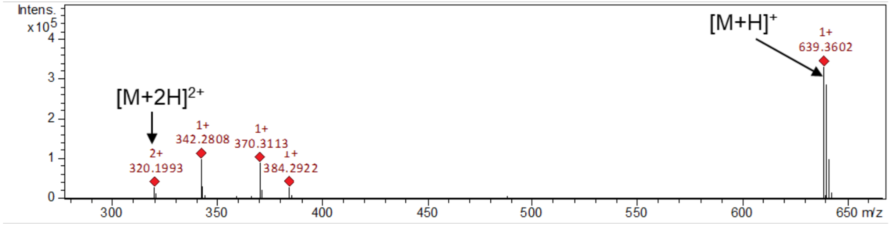

Figure S160. +ESI-QqTOF-MS spectrum of 16-deoxy-*O*[10]-obtusifuranamine-B (**19**); *m/z* 639.3602 $[M+H]^+$, 320.1993 $[M+2H]^{2+}$.

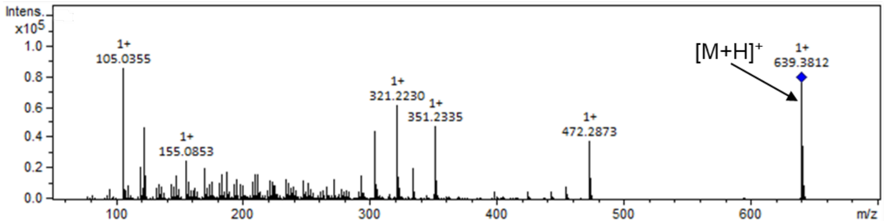

Figure S161. +ESI-QqTOF MS/MS spectrum of 16-deoxy-*O*[10]-obtusifuranamine-B (**19**).

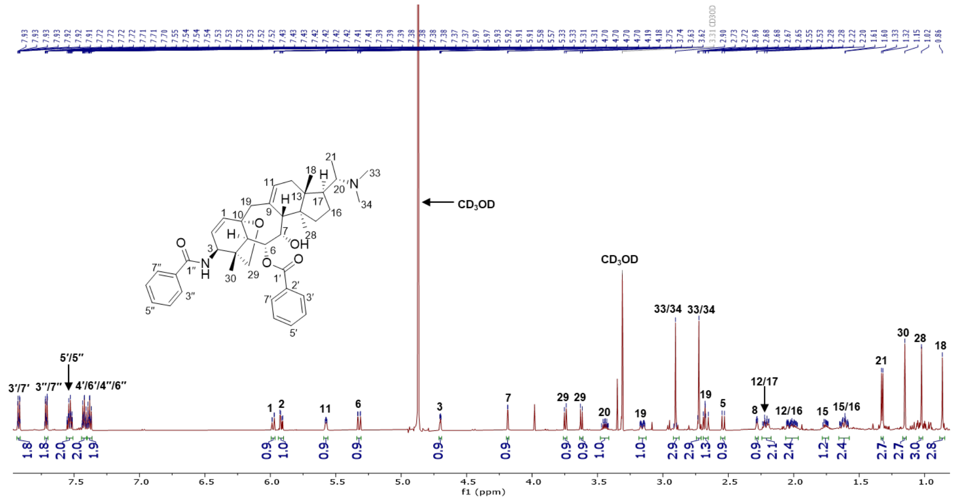

Figure S162. ^{1}H NMR spectrum of 16-deoxy-*O*[10]-obtusifuranamine-B (**19**) (CD_3OD, 600 MHz).

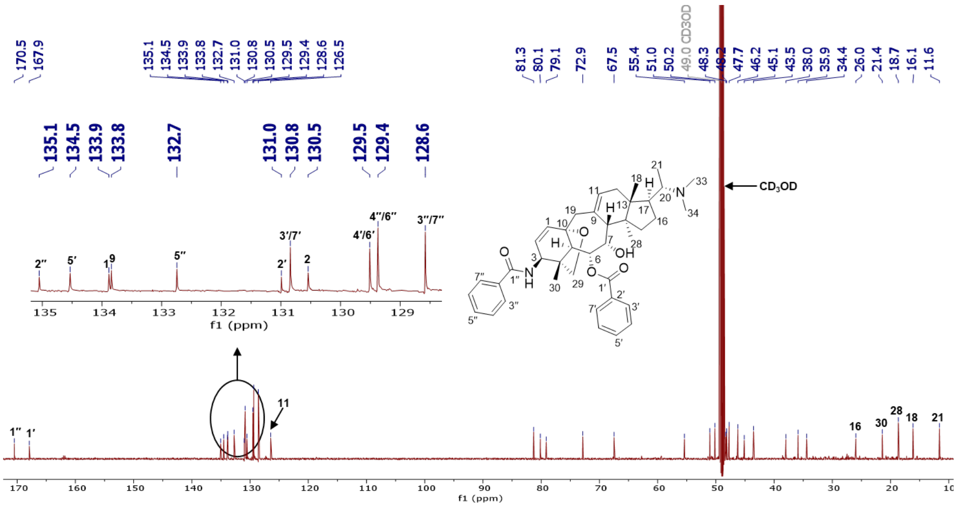

Figure S163. ^{13}C NMR spectrum of 16-deoxy-*O*[10]-obtusifuranamine-B (**19**) (CD_3OD, 150 MHz).

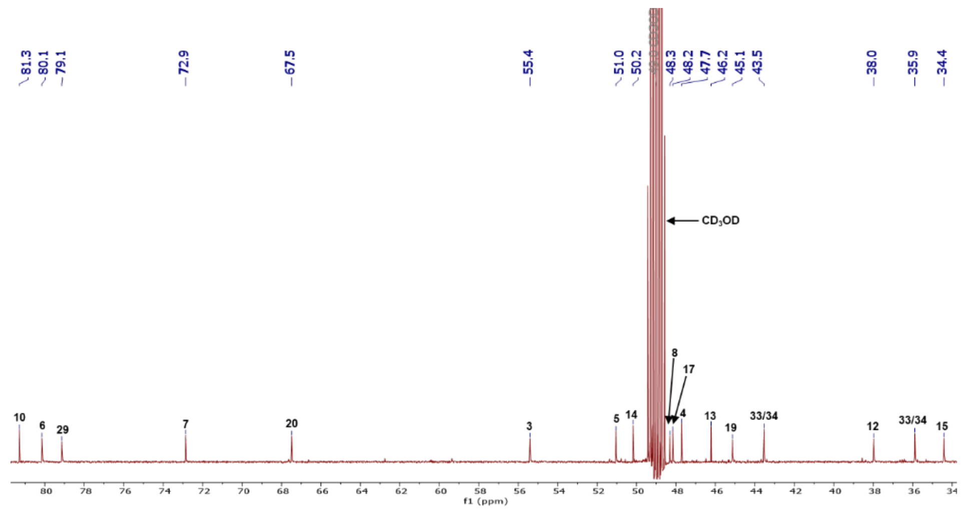

Figure S164. Detail of the ^{13}C NMR spectrum of 16-deoxy-*O*[10]-obtusifuranamine-B (**19**) (CD_3OD, 150 MHz).

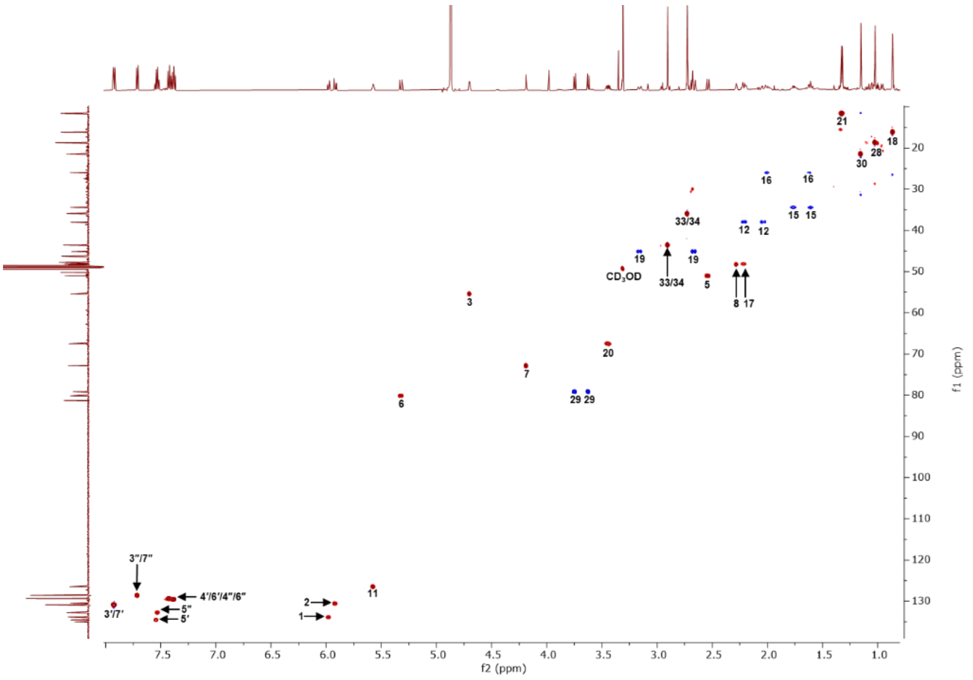

Figure S165. $^{1}H/^{13}C$ HSQC spectrum of 16-deoxy-*O*[10]-obtusifuranamine-B (**19**) (CD_3OD, 600/150 MHz).

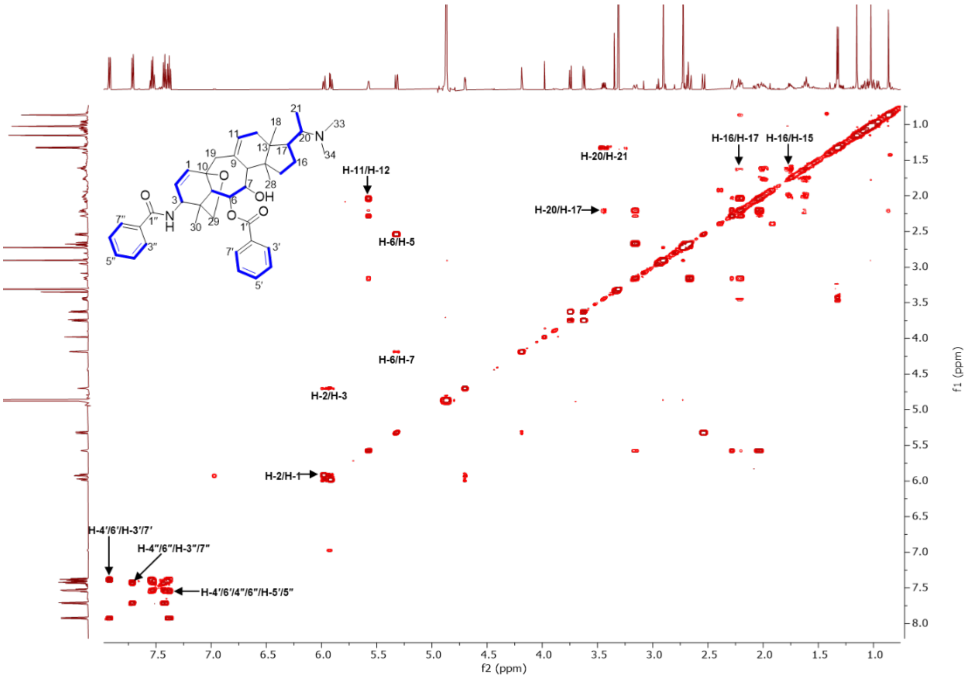

Figure S166. $^{1}H/^{1}H$ COSY spectrum (key correlations) of 16-deoxy-*O*[10]-obtusifuranamine-B (**19**) (CD_3OD, 600 MHz).

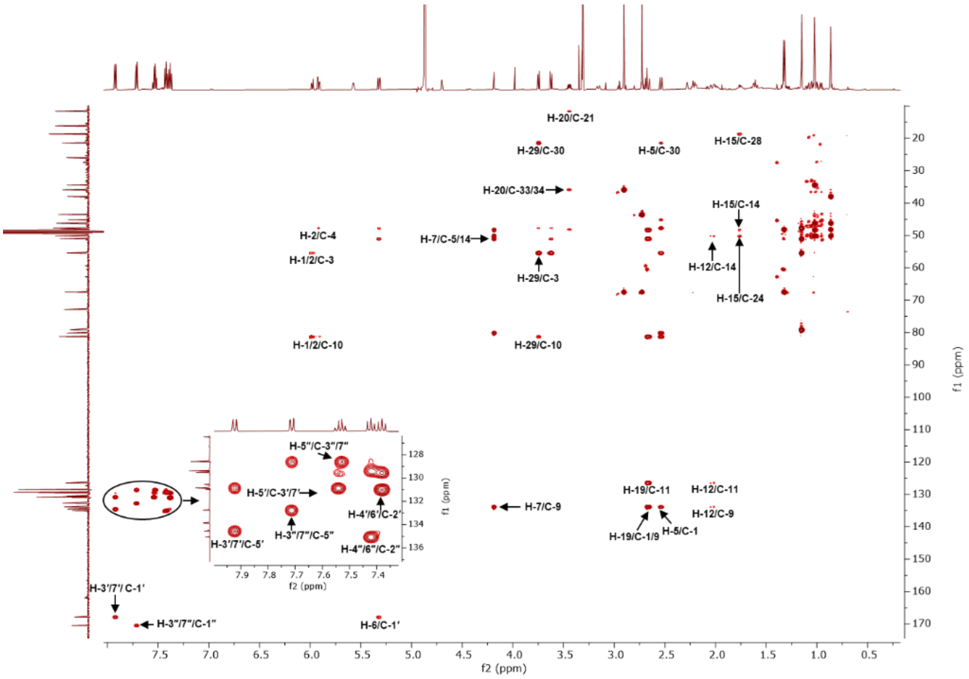

Figure S167. $^{1}H/^{13}C$ HMBC spectrum (key correlations) of 16-deoxy-*O*[10]-obtusifuranamine-B (**19**) (CD_3OD, 600/150 MHz).

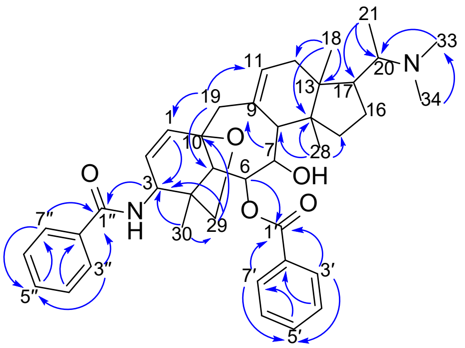

Figure S168. Key HMBC (blue arrows) correlations of 16-deoxy-*O*[10]-obtusifuranamine-B (**19**).

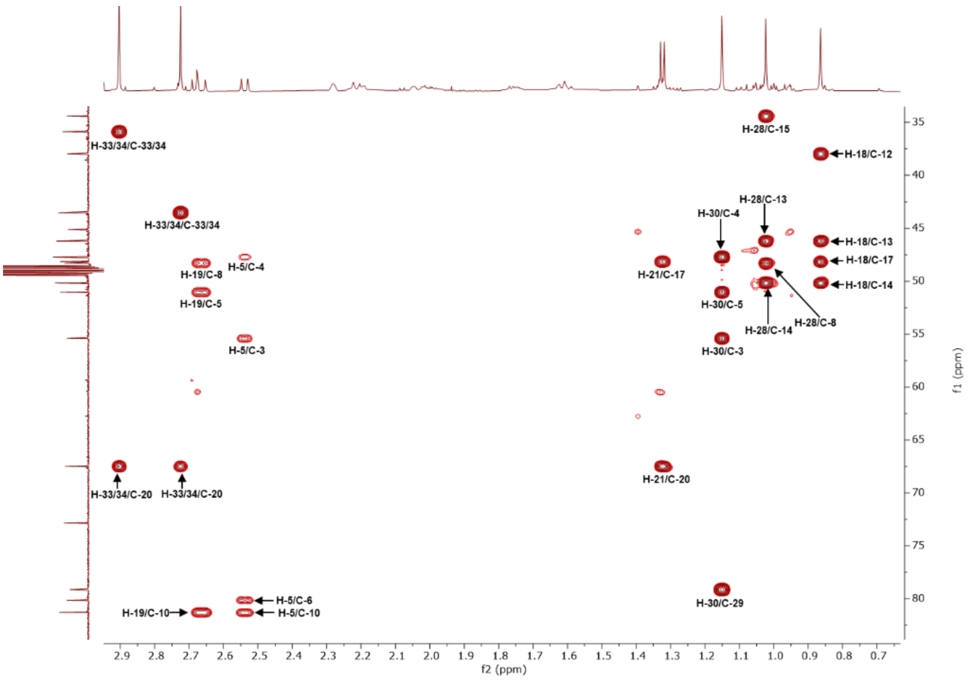

Figure S169. $^1H/^{13}C$ HMBC spectrum (key correlations) of 16-deoxy-*O*[10]-obtusifuranamine-B (**19**) (CD_3OD, 600/150 MHz).

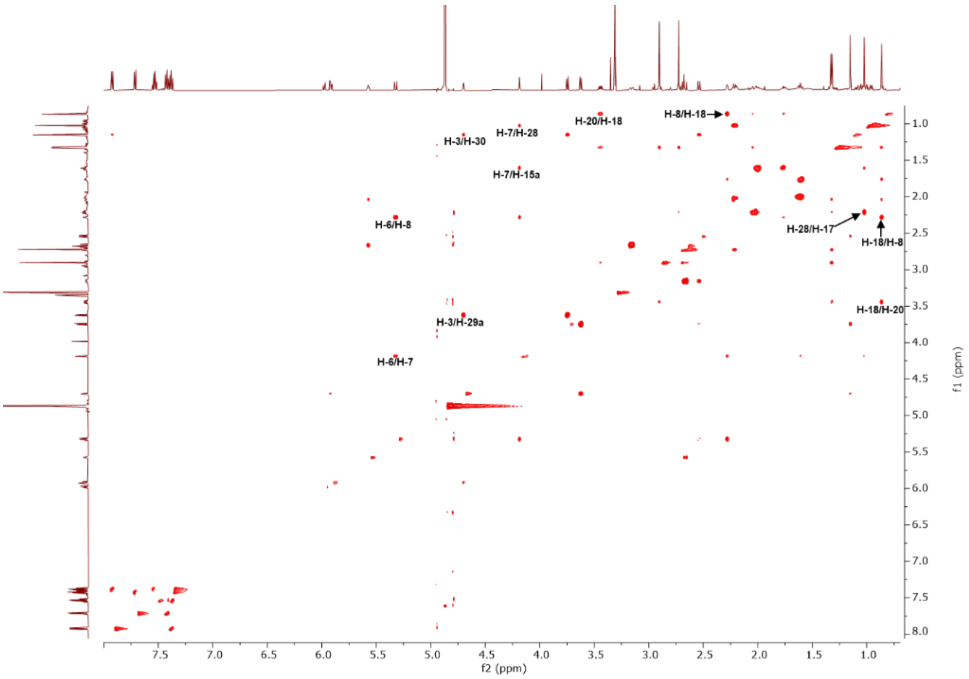

Figure S170. $^1H/^1H$ NOESY spectrum (key correlations) of 16-deoxy-*O*[10]-obtusifuranamine-B (**19**) (CD_3OD, 600 MHz).

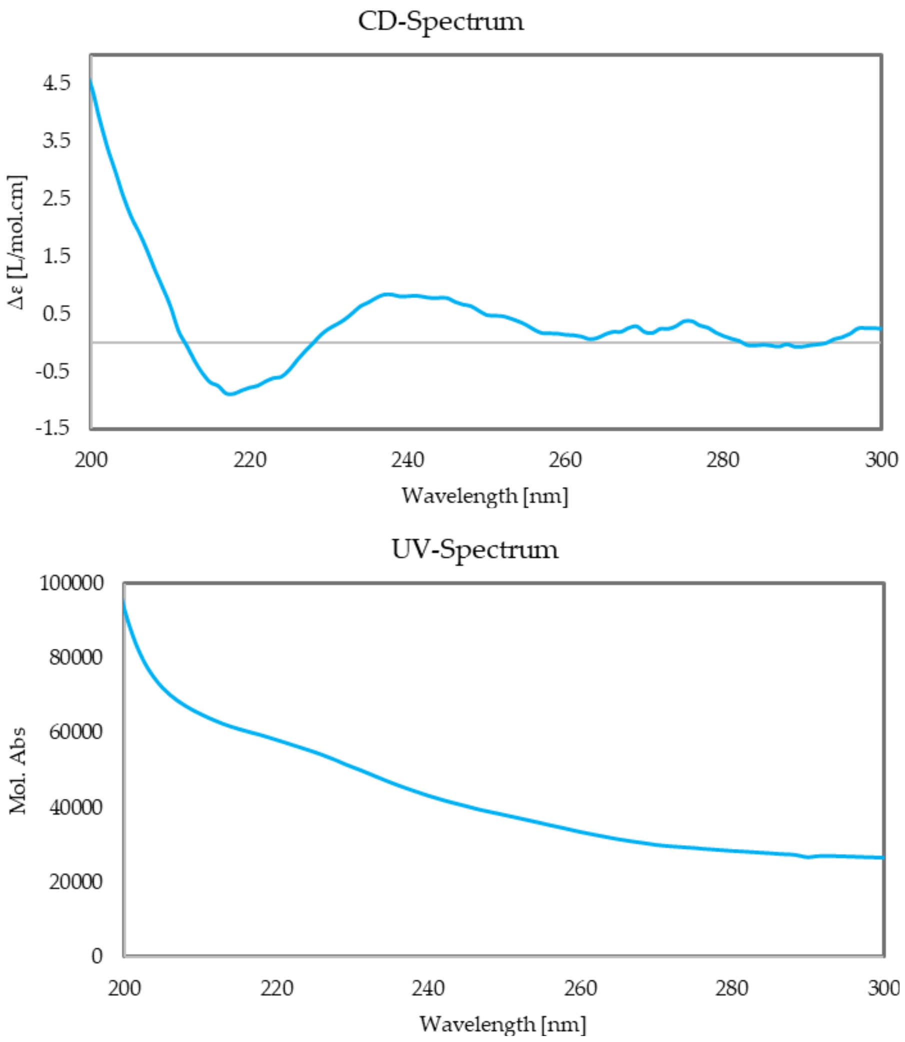

Figure S171. CD and UV spectra of obtusiepoxamine-A (**21**) in MeOH (0.1 mg/mL). CD (λ[nm], Δε): 238, 0.83; 218, -0.89; 200, 4.4 (last reading); UV (λ[nm], log ε): ≈220, ≈4.75 (sh); 200, 5.97 (last reading).

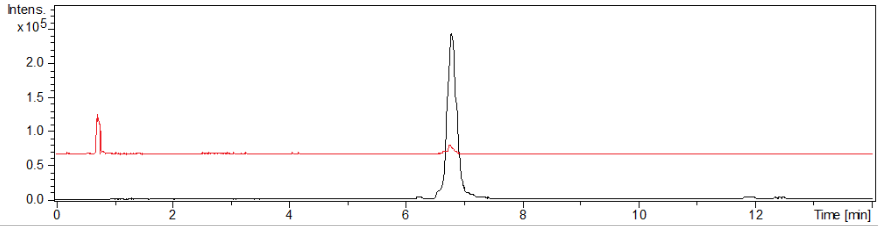

Figure S172. UHPLC/+ESI-QqTOF-MS/MS chromatogram of obtusiepoxamine-A (**21**). Base peak chromatogram 200.0000–1000.0000 +All MS (black); UV chromatogram 200-400 nm (red).

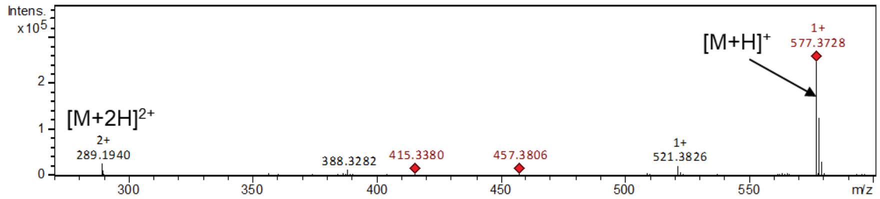

Figure S173. +ESI-QqTOF-MS spectrum of obtusiepoxamine-A (**21**); *m/z* 577.3728 $[M+H]^+$, 289.1940 $[M+2H]^{2+}$.

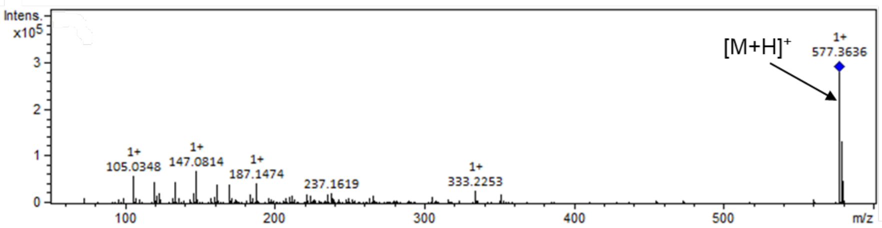

Figure S174. +ESI-QqTOF MS/MS spectrum of obtusiepoxamine-A (**21**).

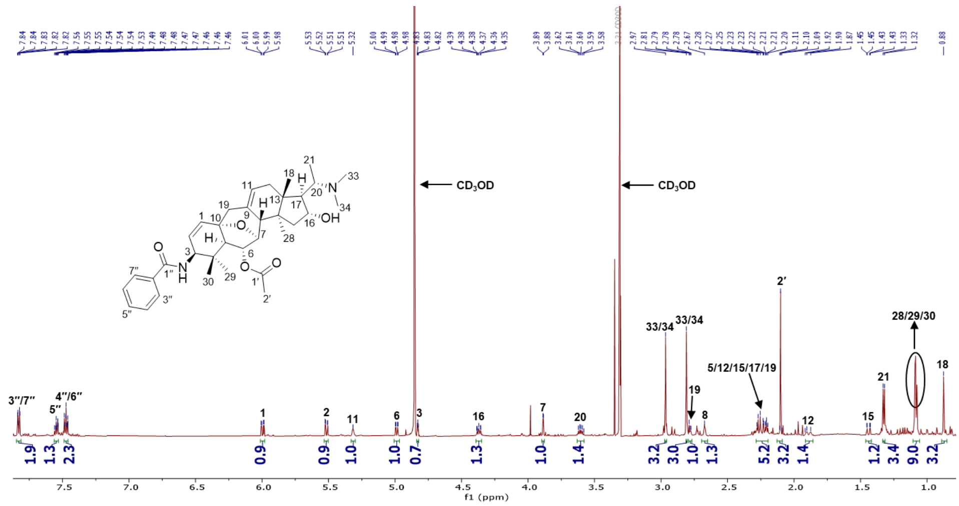

Figure S175. ^{1}H NMR spectrum of obtusiepoxamine-A (**21**) (CD_3OD, 600 MHz).

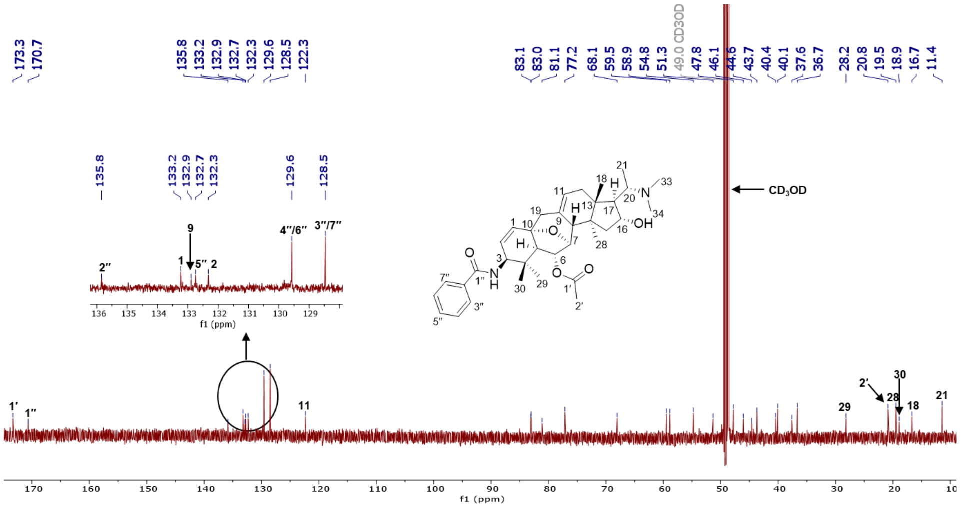

Figure S176. ^{13}C NMR spectrum of obtusiepoxamine-A (**21**) (CD_3OD, 150 MHz).

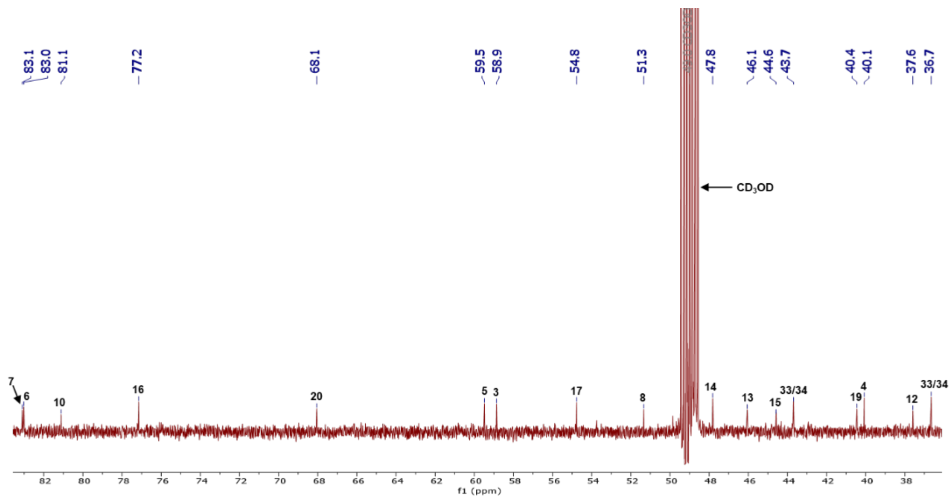

Figure S177. Detail of the ^{13}C NMR spectrum of obtusiepoxamine-A (**21**) (CD_3OD, 150 MHz).

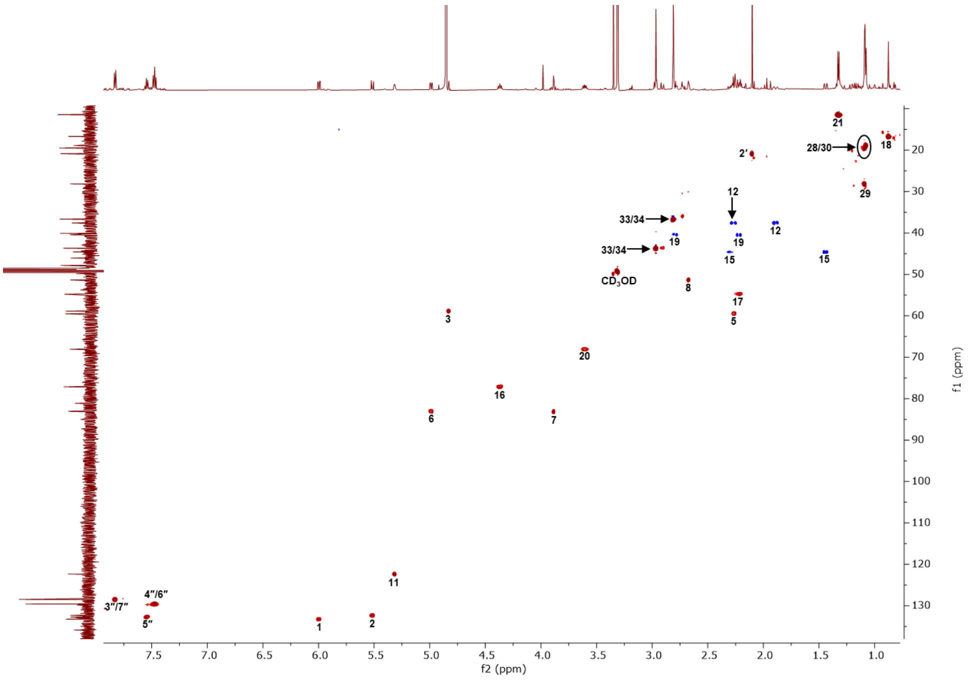

Figure S178. ^{1}H/^{13}C HSQC spectrum of obtusiepoxamine-A (**21**) (CD_3OD, 600/150 MHz).

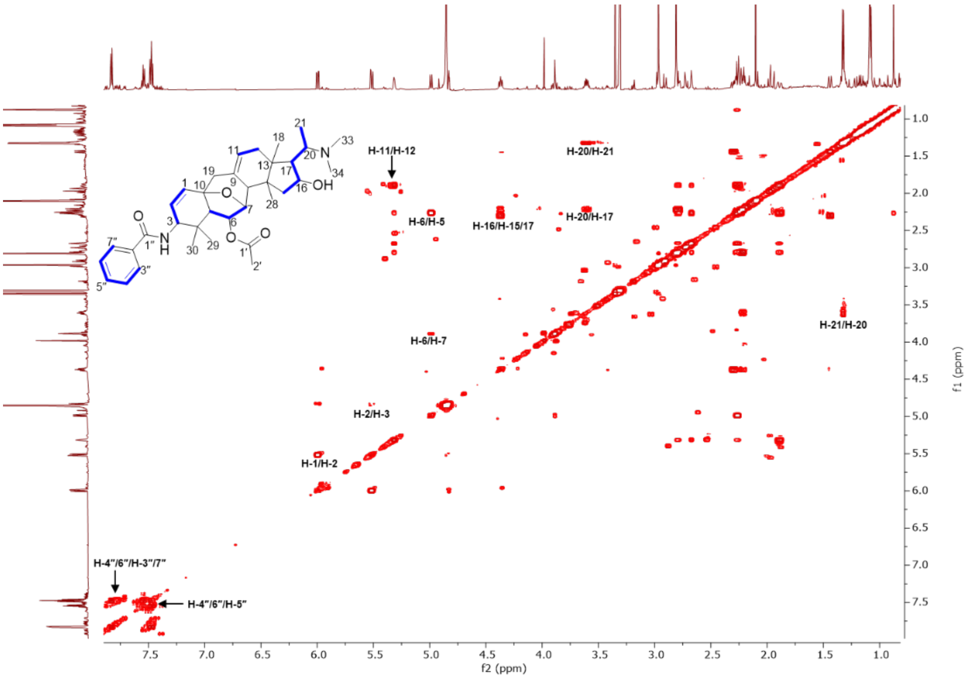

Figure S179. $^{1}H/^{1}H$ COSY spectrum (key correlations) of obtusiepoxamine-A (**21**) (CD_3OD, 600 MHz).

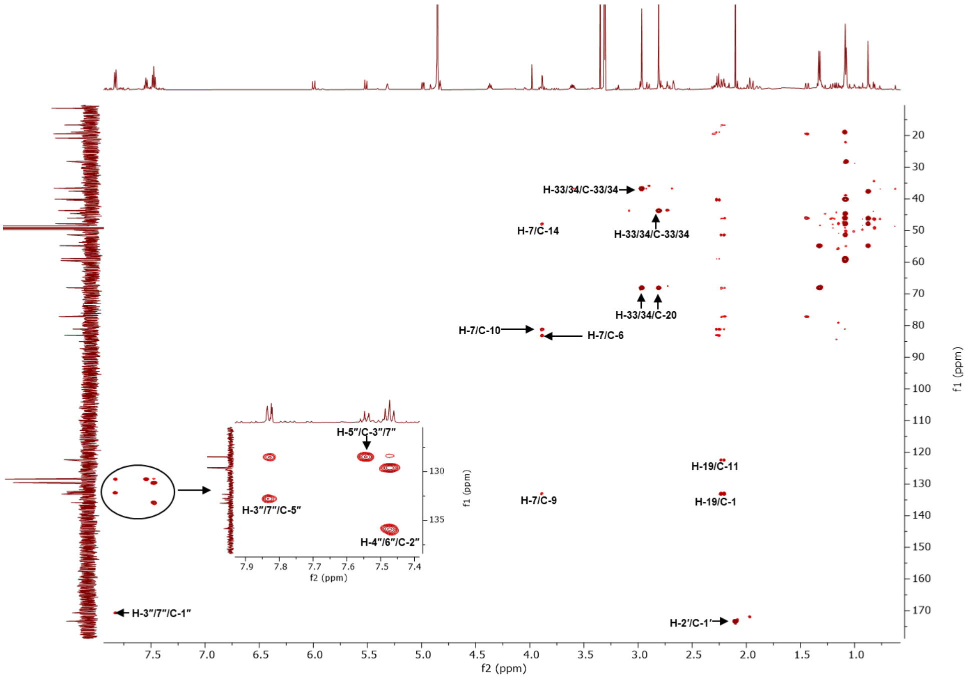

Figure S180. $^{1}H/^{13}C$ HMBC spectrum (key correlations) of obtusiepoxamine-A (**21**) (CD_3OD, 600/150 MHz).

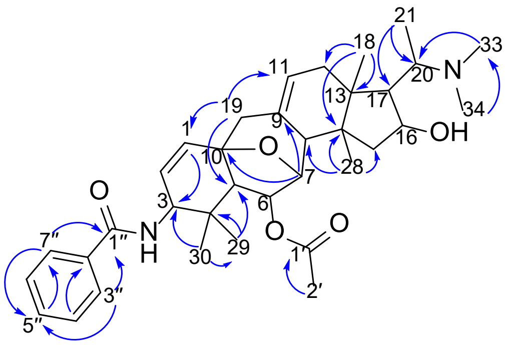

Figure S181. Key HMBC (blue arrows) correlations of obtusiepoxamine-A (**21**).

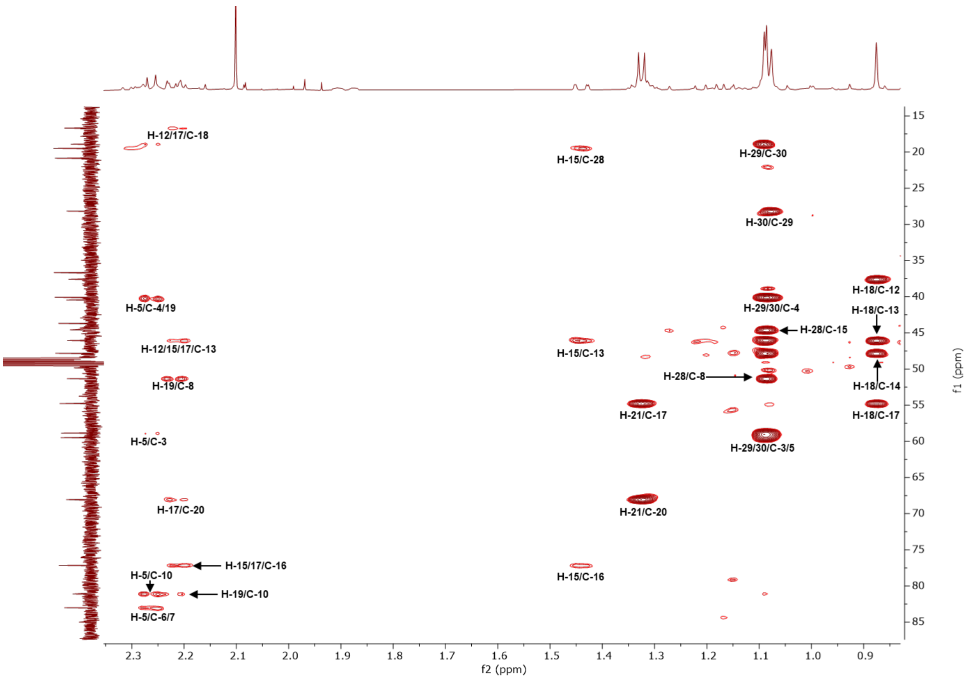

Figure S182. Detail of the $^{1}H/^{13}C$ HMBC spectrum (key correlations) of obtusiepoxamine-A (**21**) (CD_3OD, 600/150 MHz).

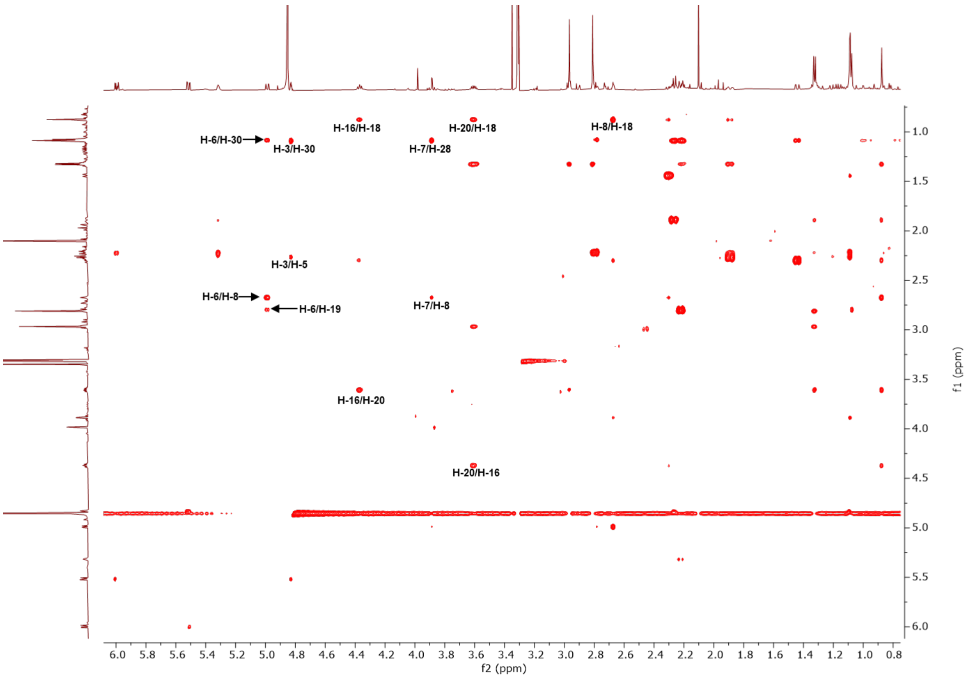

Figure S183. $^1H/^1H$ NOESY spectrum (key correlations) of obtusiepoxamine-A (**21**) (CD_3OD, 600 MHz).

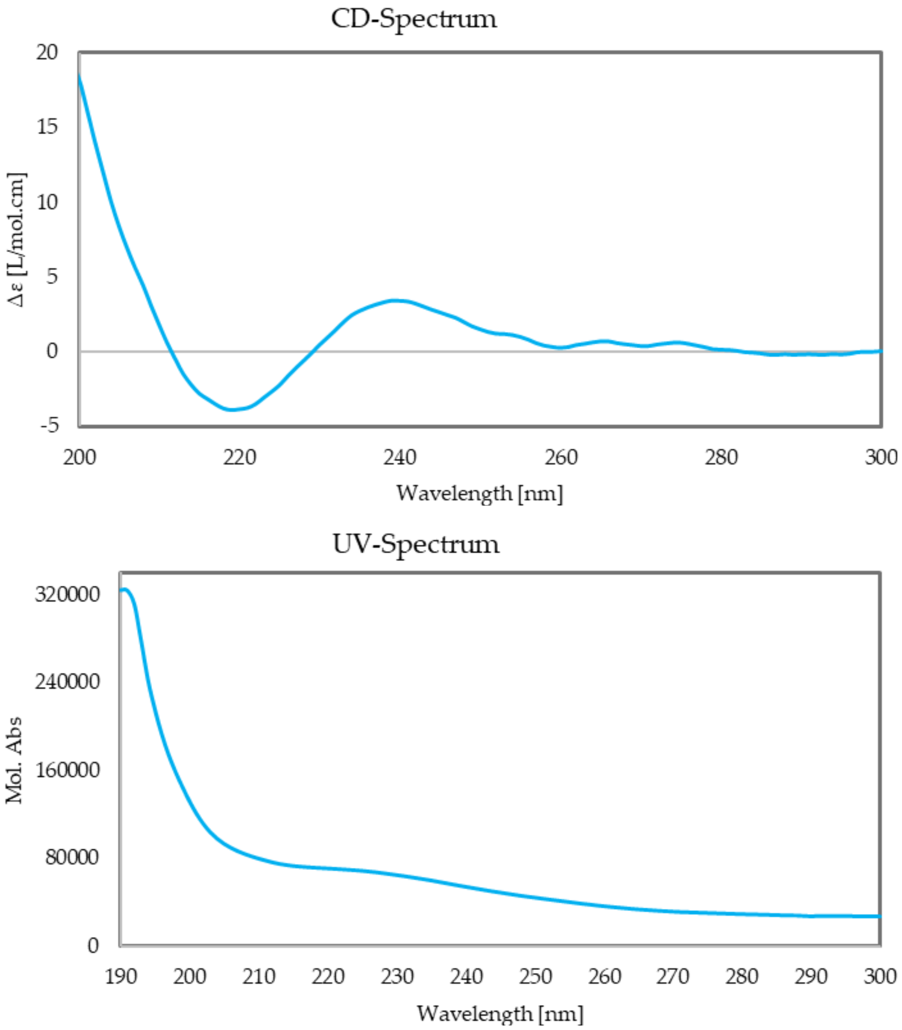

Figure S184. CD and UV spectra of obtusidienolamine-A (**22**) in MeOH (0.1 mg/mL). CD (λ[nm], Δε): 239, 3.38; 219, -3.94; 200, 18.6 (last reading); UV (λ[nm], log ε): 225, 4.8 (sh); 200, 5.12 (last reading).

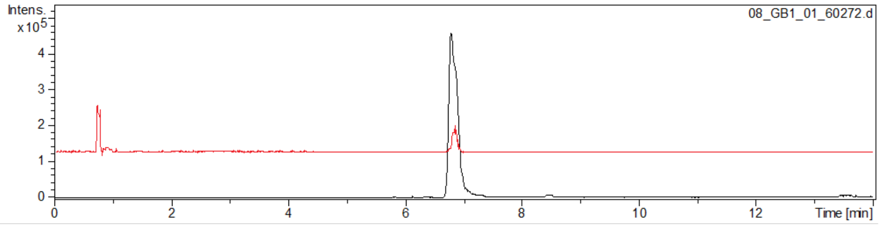

Figure S185. UHPLC/+ESI-QqTOF-MS/MS chromatogram of obtusidienolamine-A (**22**). Base peak chromatogram 200.0000–1000.0000 +All MS (black); UV chromatogram 200-400 nm (red).

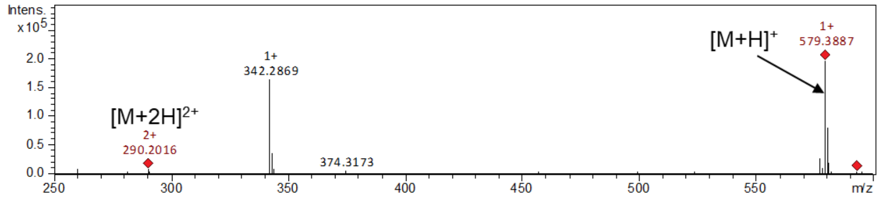

Figure S186. +ESI-QqTOF-MS spectrum of obtusidienolamine-A (**22**); *m/z* 579.3887 $[M+H]^+$, 290.2016 $[M+2H]^{2+}$.

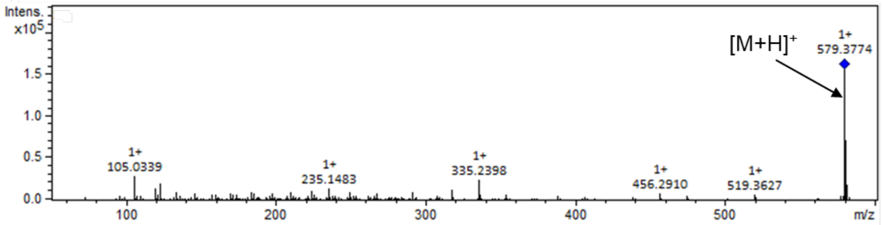

Figure S187. +ESI-QqTOF MS/MS spectrum of obtusidienolamine-A (**22**).

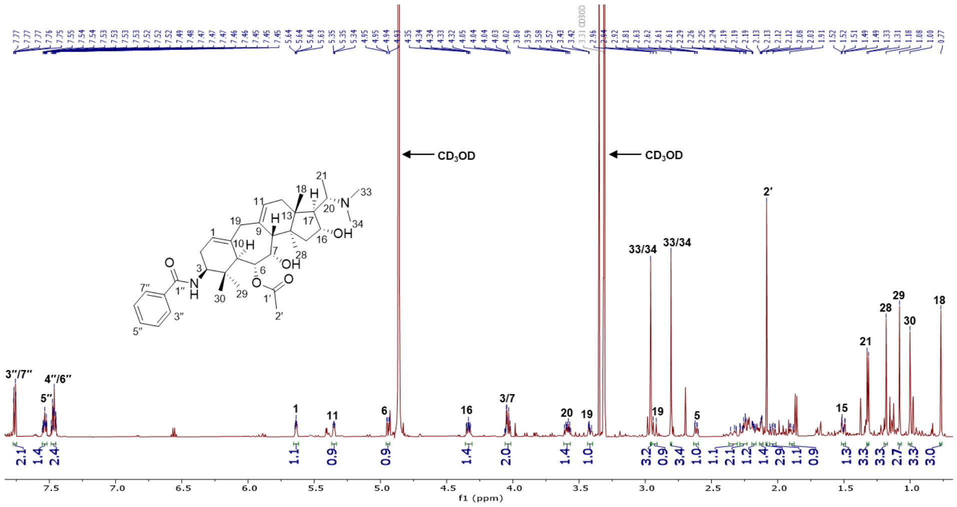

Figure S188. ^{1}H NMR spectrum of obtusidienolamine-A (**22**) (CD3OD, 600 MHz).

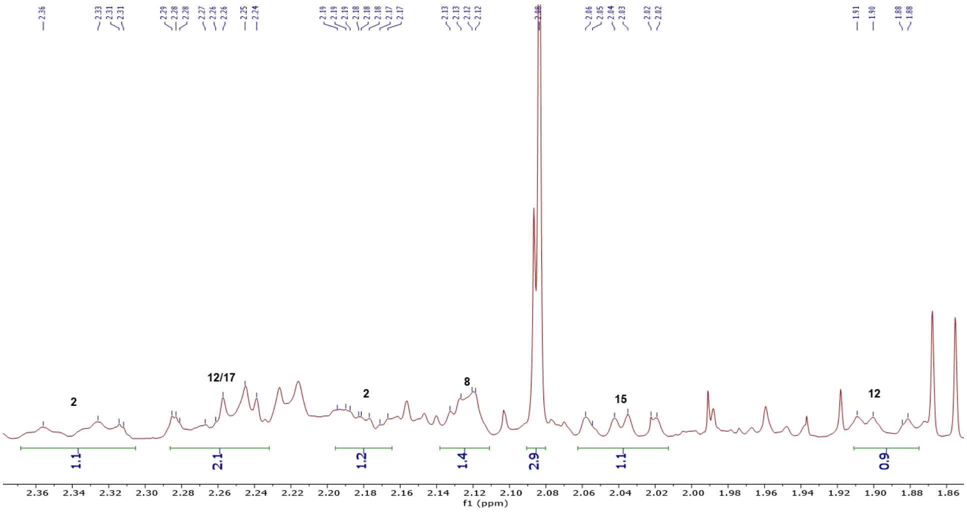

Figure S189. Detail of the ^{1}H NMR spectrum of obtusidienolamine-A (**22**) (CD3OD, 600 MHz).

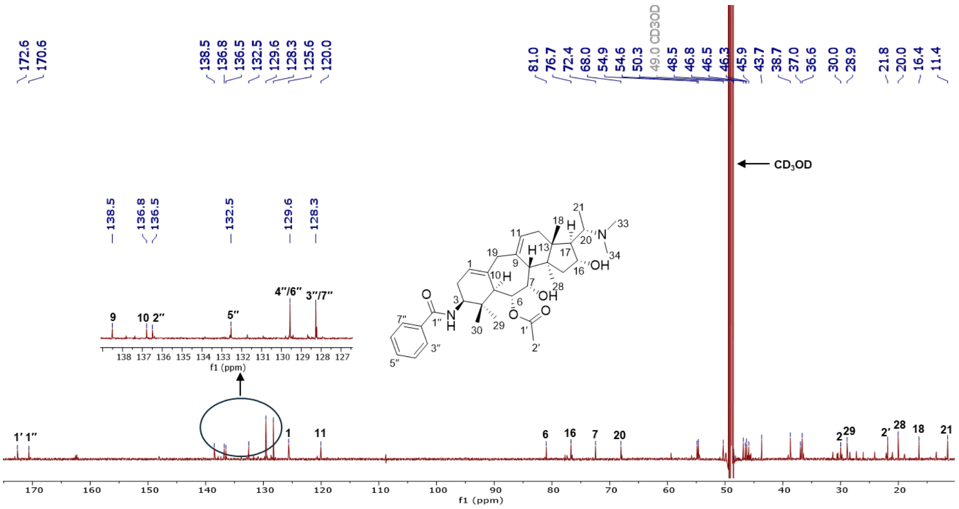

Figure S190. ^{13}C NMR spectrum of obtusidienolamine-A (**22**) (CD_3OD, 150 MHz).

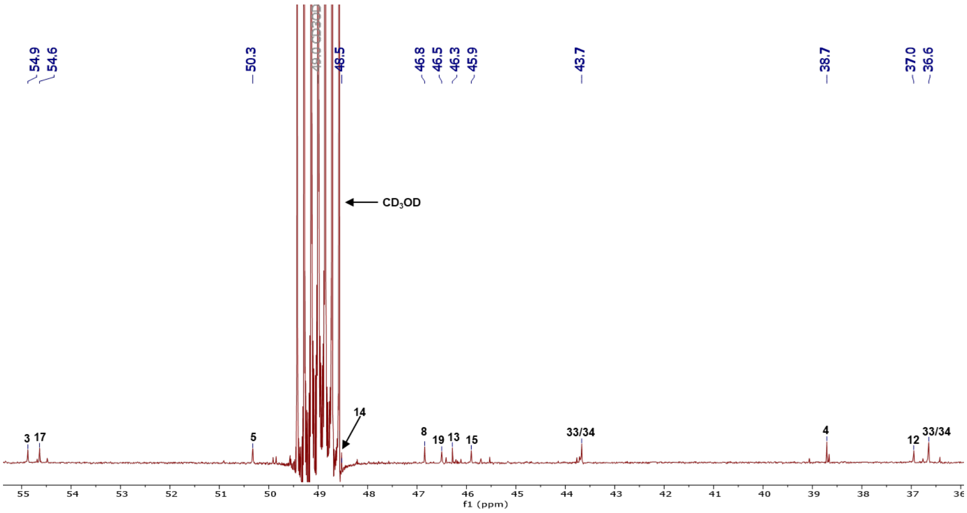

Figure S191. Detail of the ^{13}C NMR spectrum of obtusidienolamine-A (**22**) (CD_3OD, 150 MHz).

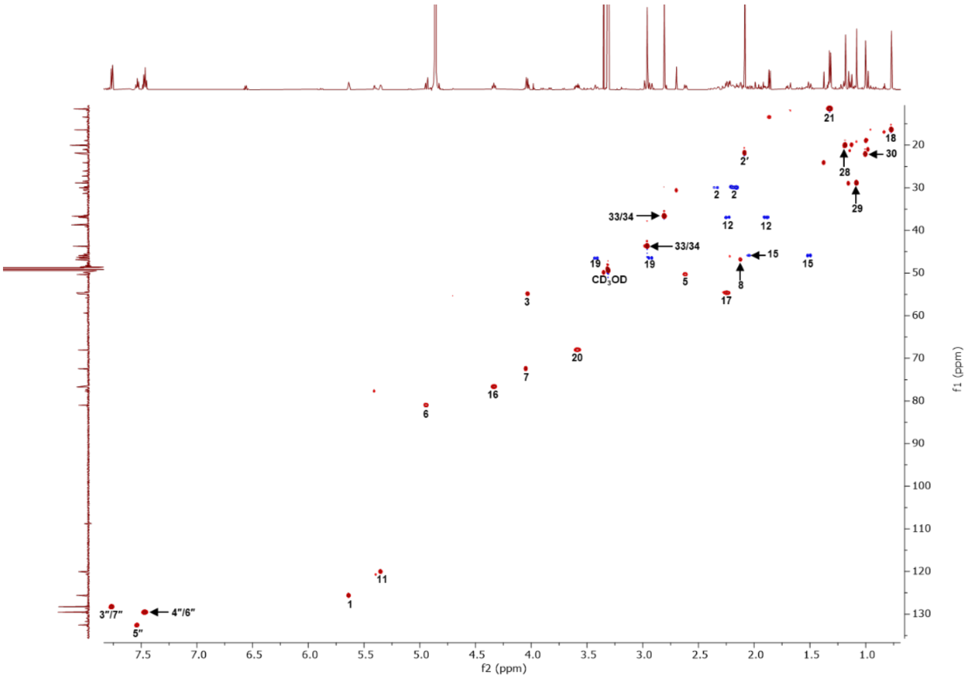

Figure S192. $^{1}H/^{13}C$ HSQC spectrum of obtusidienolamine-A (**22**) (CD_3OD, 600/150 MHz).

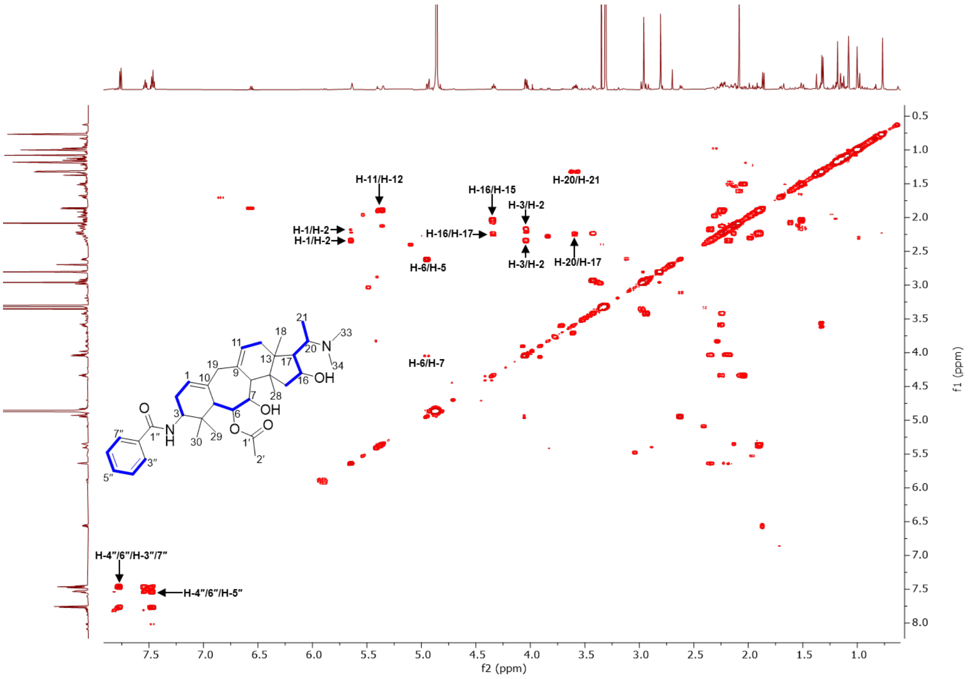

Figure S193. $^1H/^1H$ COSY spectrum (key correlations) of obtusidienolamine-A (**22**) (CD_3OD, 600 MHz).

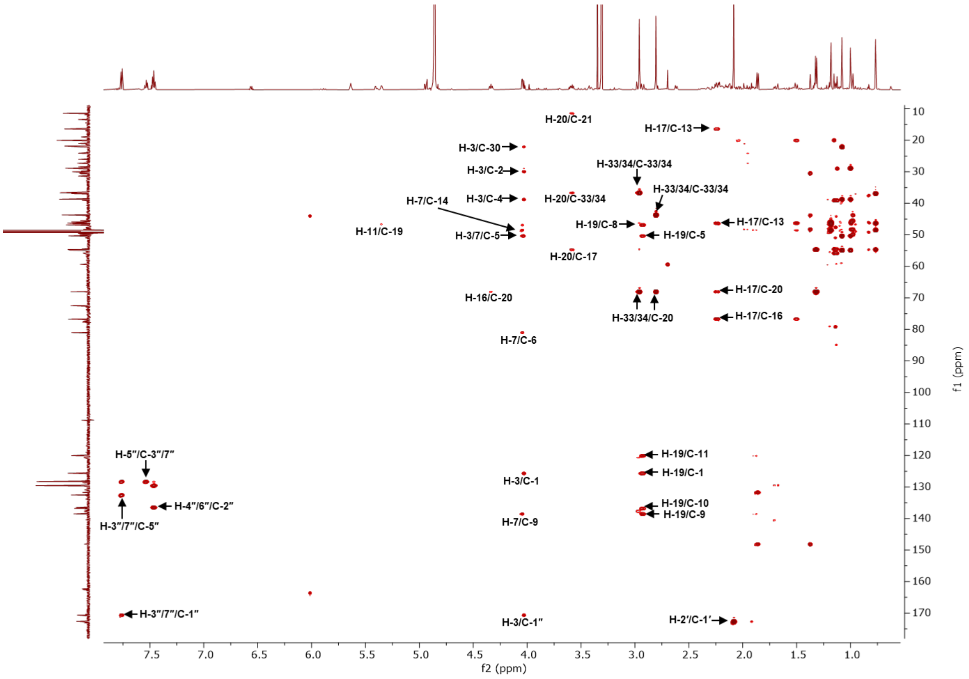

Figure S194. $^1H/^{13}C$ HMBC spectrum (key correlations) of obtusidienolamine-A (**22**) (CD_3OD, 600/150 MHz).

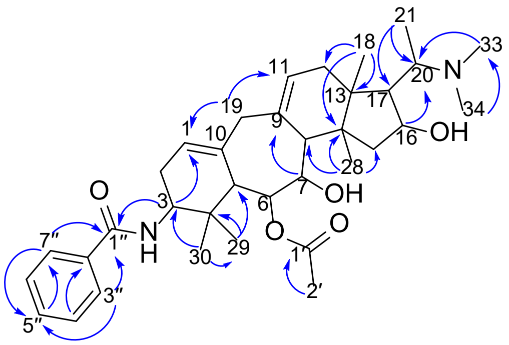

Figure S195. Key HMBC (blue arrows) correlations of obtusidienolamine-A (**22**).

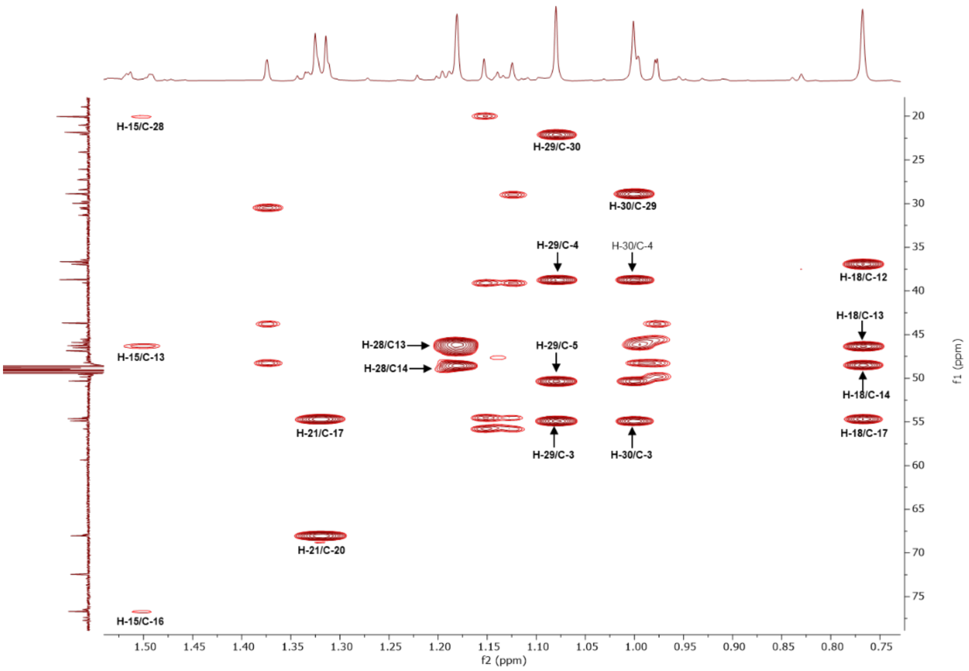

Figure S196. Detail of the ^{1}H/^{13}C HMBC spectrum (key correlations) of obtusidienolamine-A (**22**) (CD_3OD, 600/150 MHz).

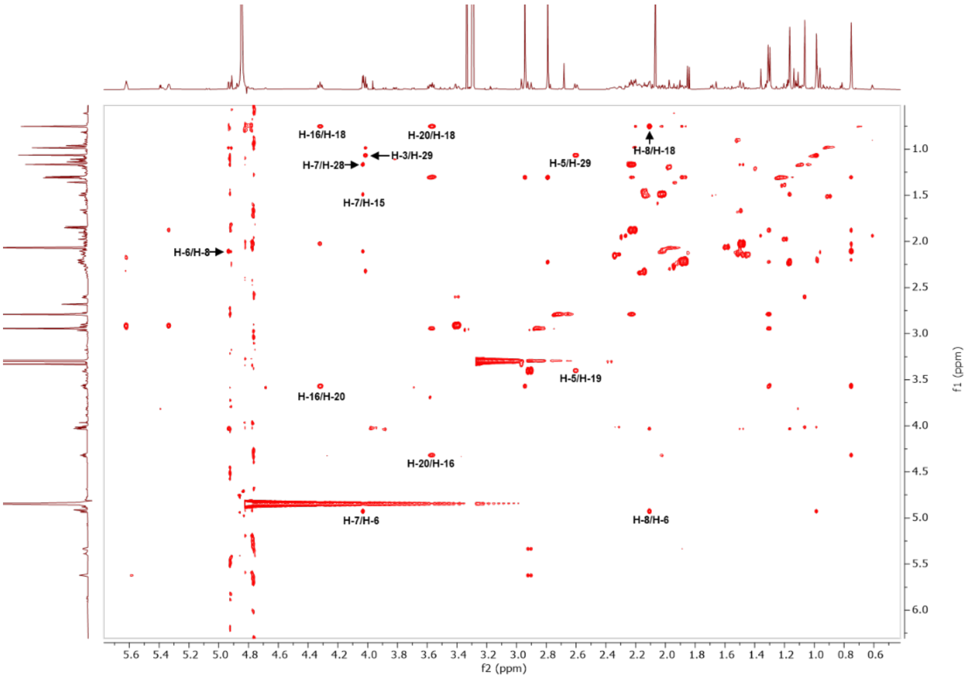

Figure S197. ^{1}H/^{1}H NOESY spectrum (key correlations) of obtusidienolamine-A (**22**) (CD_3OD, 600 MHz).

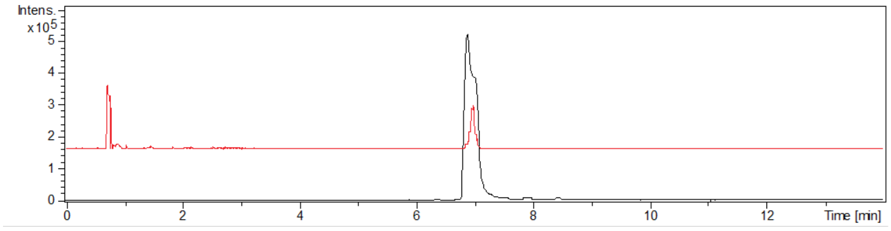

Figure S198. UHPLC/+ESI-QqTOF-MS/MS chromatogram of deoxyobtusidienolamine-A (**23**). Base peak chromatogram 200.0000–1000.0000 +All MS (black); UV chromatogram 200-400 nm (red).

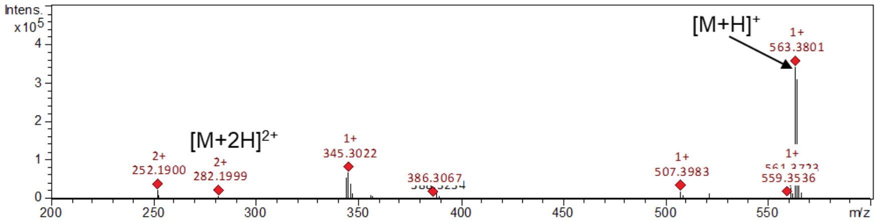

Figure S199. +ESI-QqTOF-MS spectrum of deoxyobtusidienolamine-A (**23**); *m/z* 563.3801 [M+H]$^+$, 282.1999 [M+2H]$^{2+}$.

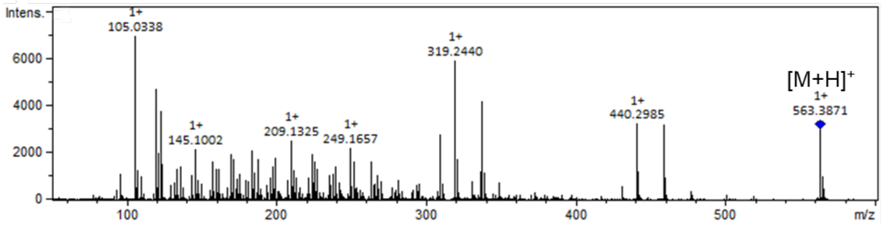

Figure S200. +ESI-QqTOF MS/MS spectrum of deoxyobtusidienolamine-A (**23**).

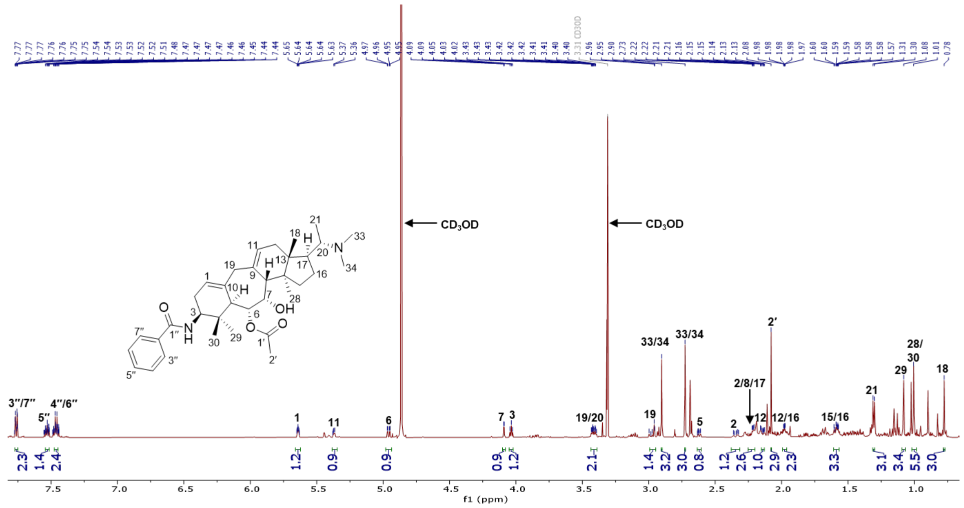

Figure S201. ^{1}H NMR spectrum of deoxyobtusidienolamine-A (**23**) (CD_3OD, 600 MHz).

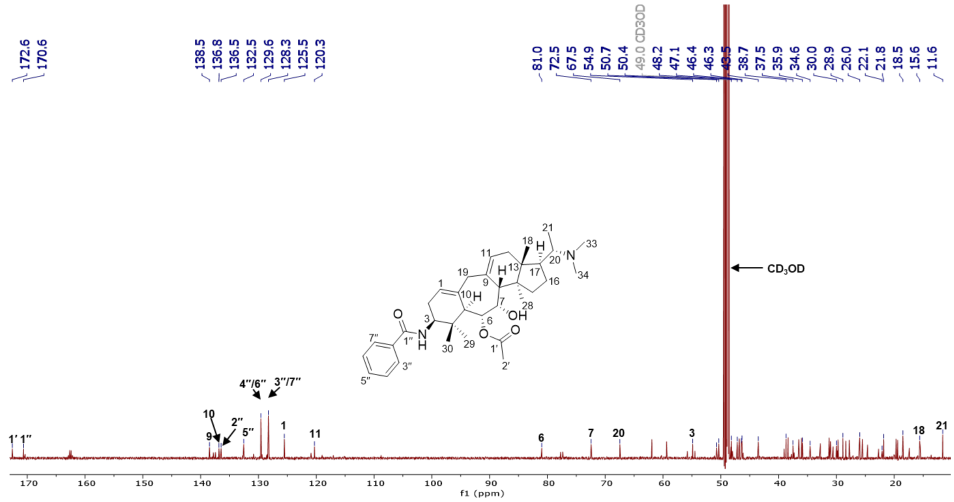

Figure S202. ^{13}C NMR spectrum of deoxyobtusidienolamine-A (**23**) (CD_3OD, 150 MHz).

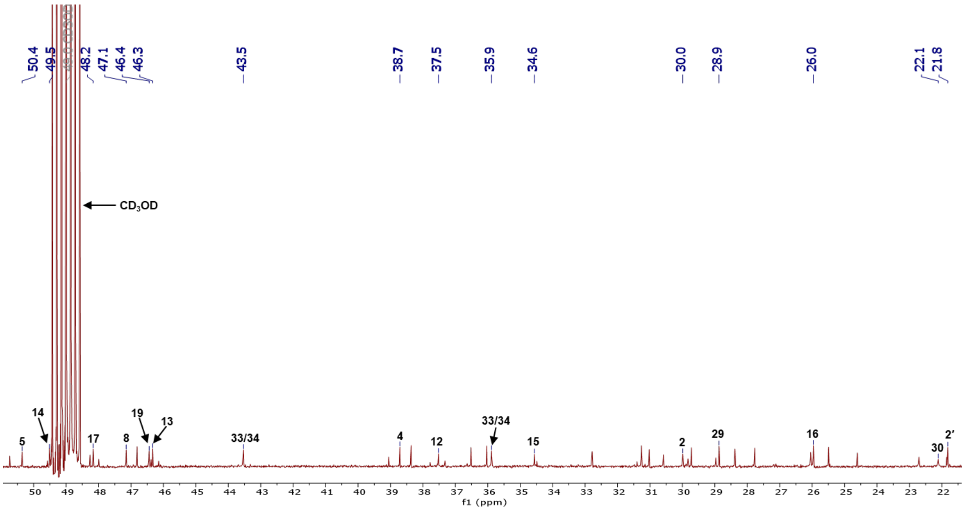

Figure S203. Detail of the ^{13}C NMR spectrum of deoxyobtusidienolamine-A (**23**) (CD_3OD, 150 MHz).

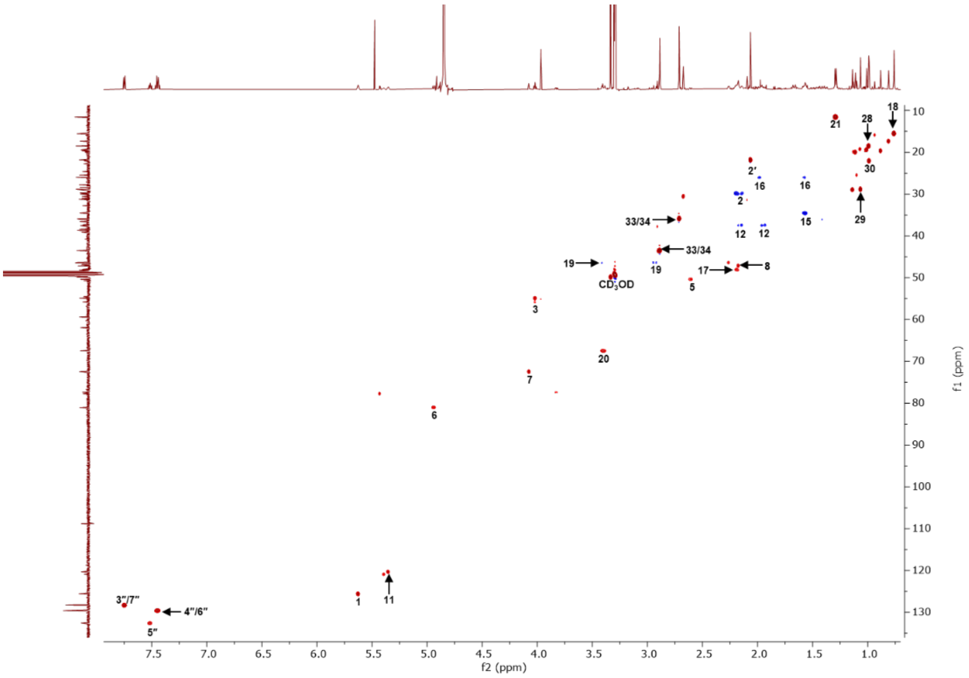

Figure S204. ^{1}H/^{13}C HSQC spectrum of deoxyobtusidienolamine-A (**23**) (CD_3OD, 600/150 MHz).

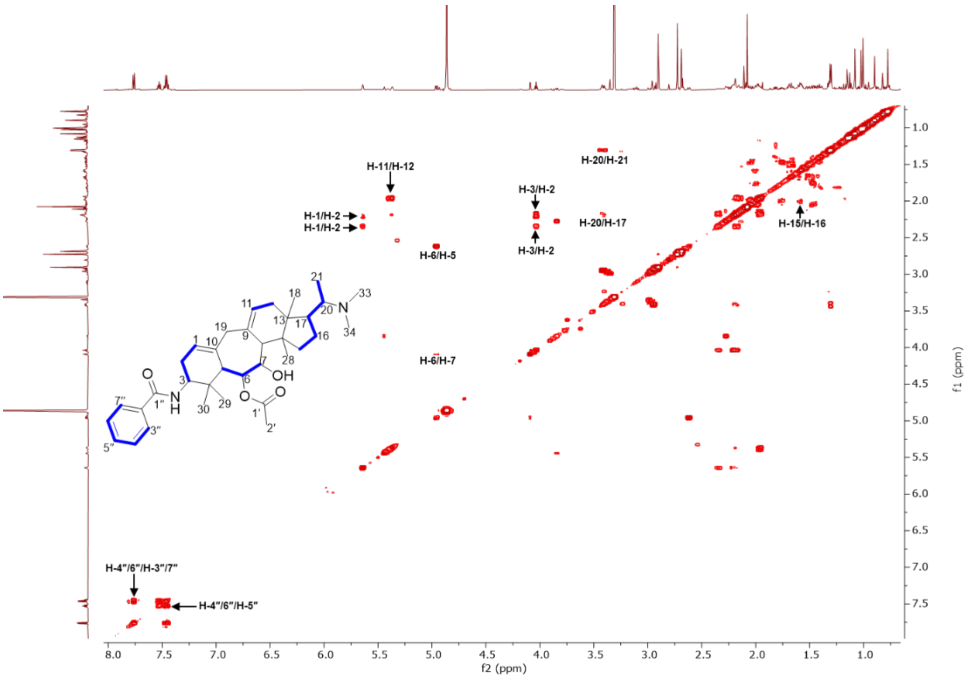

Figure S205. $^1H/^1H$ COSY spectrum (key correlations) of deoxyobtusidienolamine-A (**23**) (CD_3OD, 600 MHz).

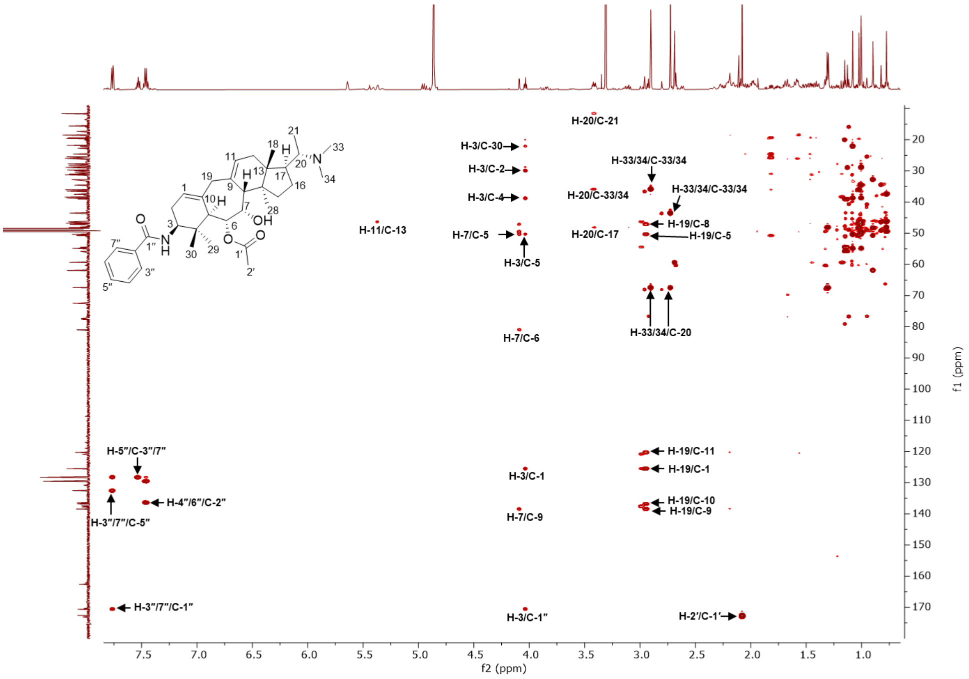

Figure S206. $^1H/^{13}C$ HMBC spectrum (key correlations) of deoxyobtusidienolamine-A (**23**) (CD_3OD, 600/150 MHz).

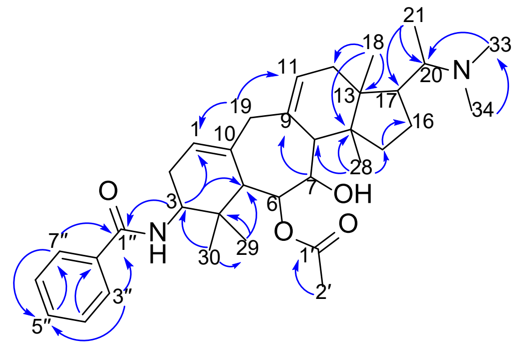

Figure 207. Key HMBC (blue arrows) correlations of of deoxyobtusidienolamine-A (**23**).

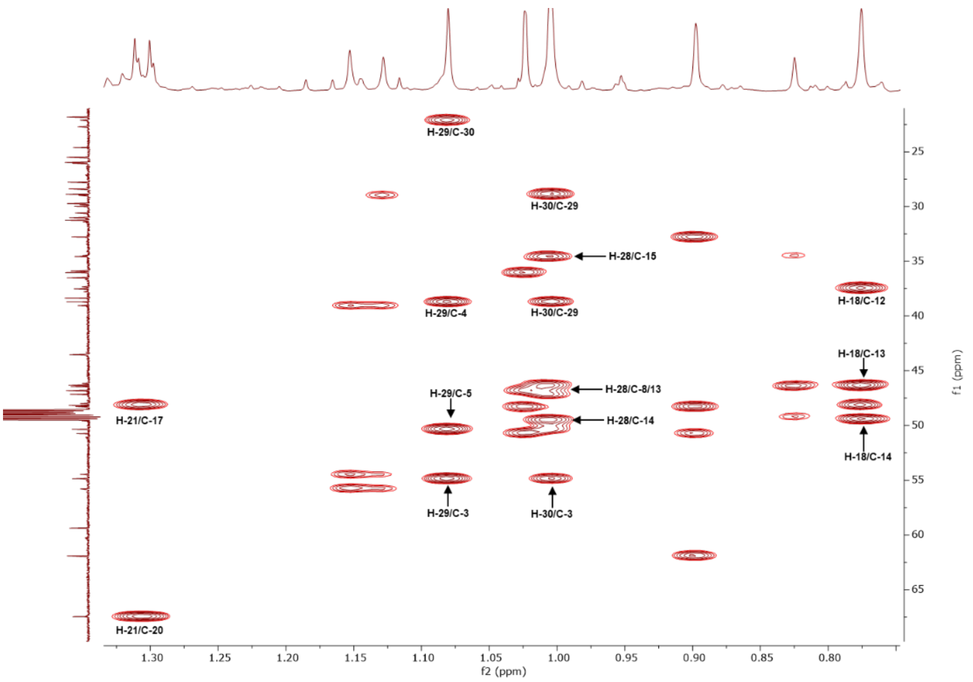

Figure S208. Detail of the ¹H/¹³C HMBC spectrum (key correlations) of deoxyobtusidienolamine-A (**23**) (CD_3OD, 600/150 MHz).

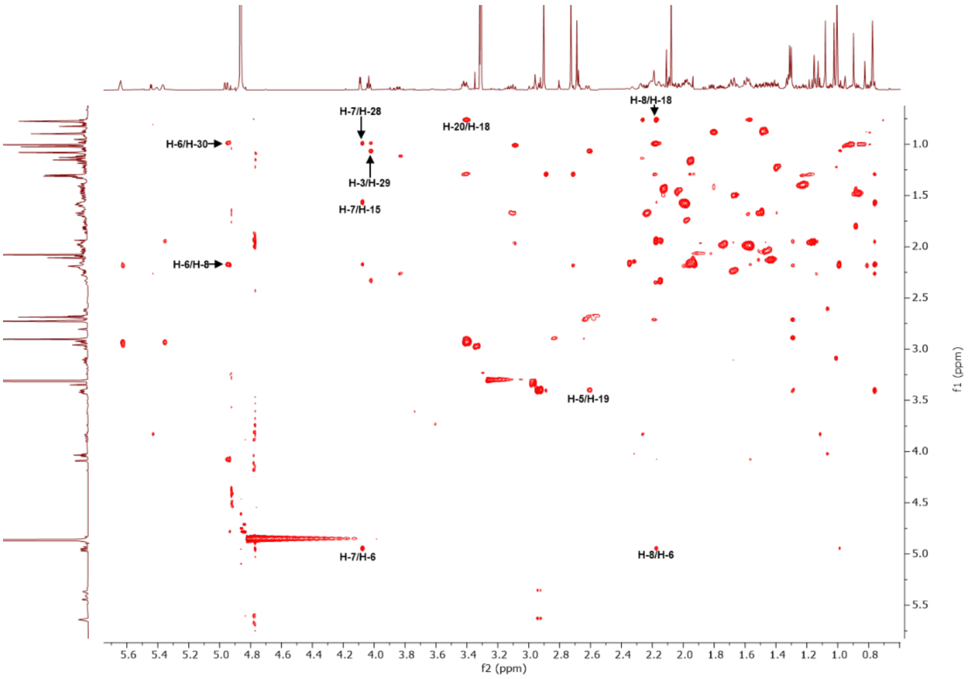

Figure S209. $^1H/^1H$ NOESY spectrum (key correlations) of deoxyobtusidienolamine-A (**23**) (CD_3OD, 600 MHz).

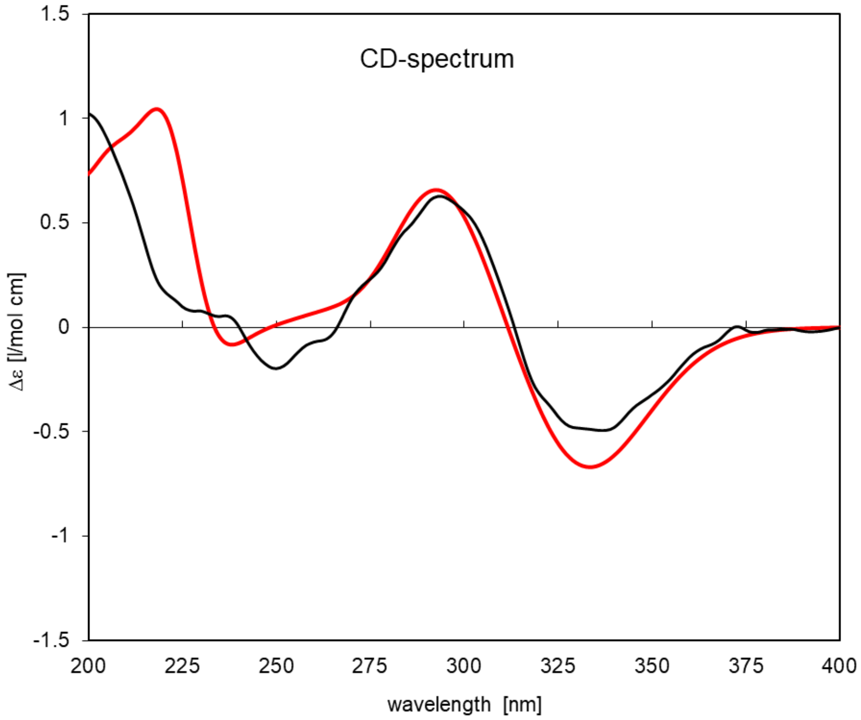

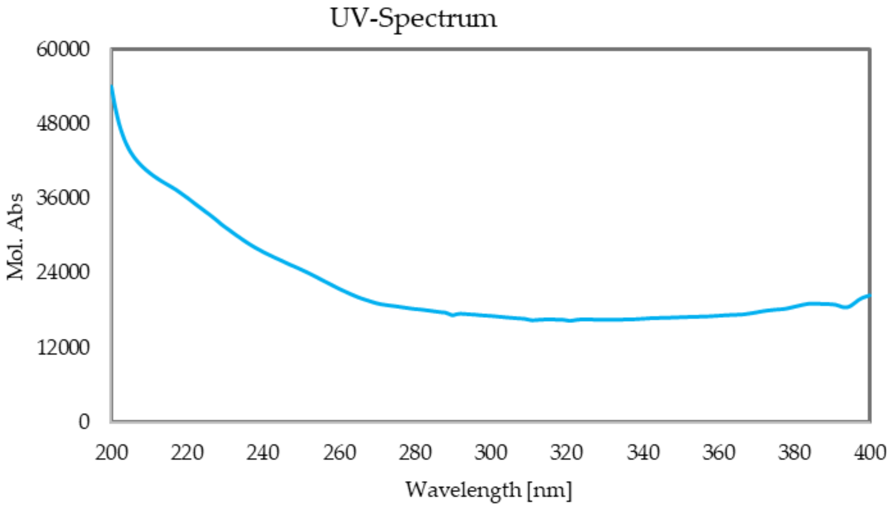

Figure S210. CD and UV spectra of obtusiaminocyclin (**24**) in MeOH (0.1 mg/mL). CD (λ[nm], Δε): 335, -0.49; 294, 0.63; 251, -0.21; 233, 0.06 (sh), 200, 1.0 (last reading); UV (λ[nm], log ε): 250, 4.4 (sh); 213, 4.6 (sh) 200, 4.7 (last reading). The CD diagram shows the experimental (black) and TD-DFT simulated spectrum (red) in direct comparison. The simulation was performed with the single conformation found in the force-field based conformational search. The structure was fully energy minimized using the B3LYP/6-31G(d,p) density functional/basis set. A TD-DFT calculation was performed with Gaussian 03 at the same level of theory considering the first 18 electronic transitions. Gaussian functions of a width at 1/e height σ=0.125 eV were applied to the resulting transition vectors and the 18 gaussian curves summed up to the spectrum shown. An energy shift of +0.1 eV and intensity scaling by factor 0.33 were applied to the original computational result to obtain the graph plotted in red, along with the experimental curve (black).

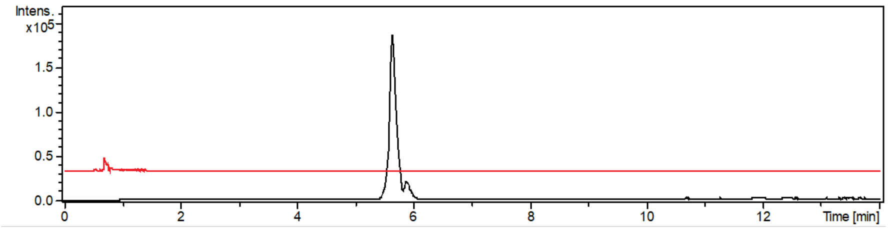

Figure 211. UHPLC/+ESI-QqTOF-MS/MS chromatogram of obtusiaminocyclin (**24**). Base peak chromatogram 200.0000–1000.0000 +All MS (black); UV chromatogram 200-400 nm (red).

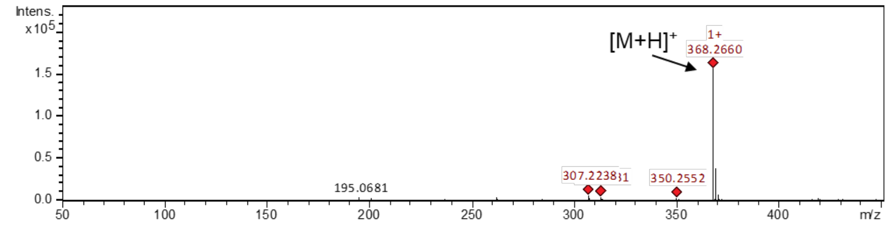

Figure S212. +ESI-QqTOF-MS spectrum of obtusiaminocyclin (**24**); *m*/*z* 368.2660 [M+H]+.

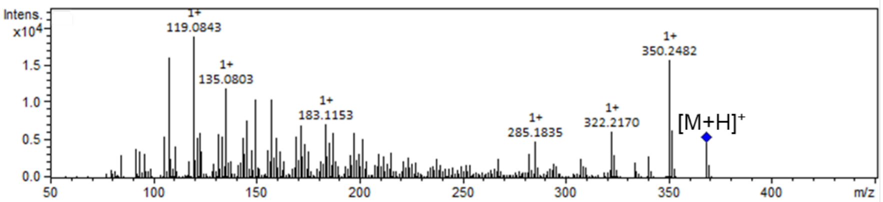

Figure S213. +ESI-QqTOF MS/MS spectrum of obtusiaminocyclin (**24**).

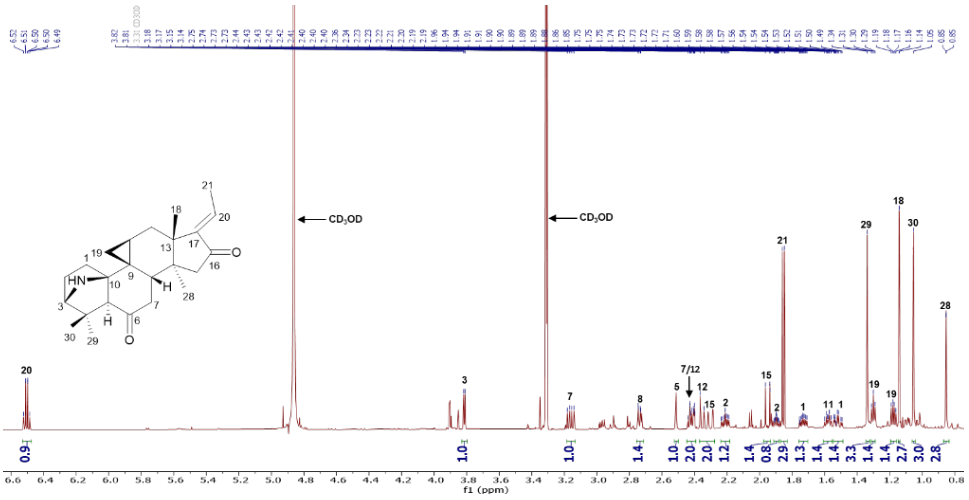

Figure S214. ^{1}H NMR spectrum of obtusiaminocyclin (**24**) (CD_3OD, 600 MHz).

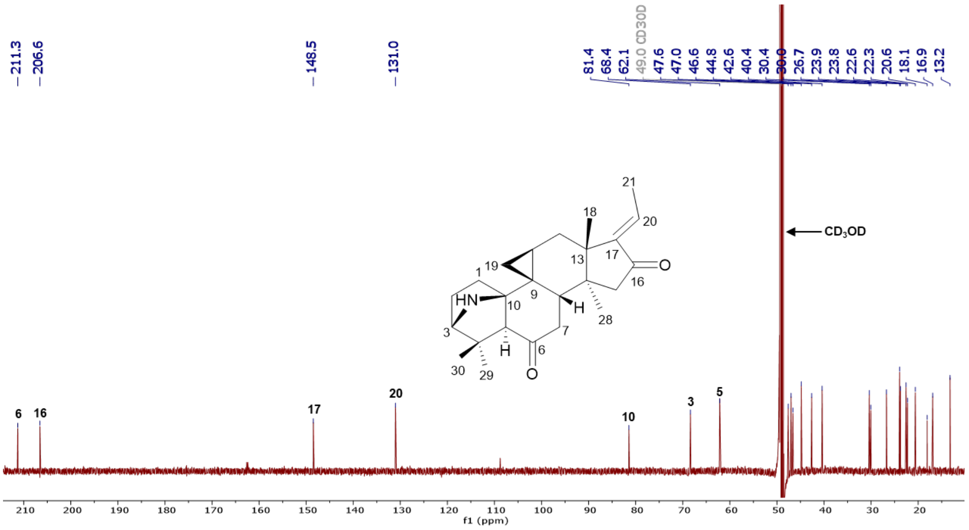

Figure S215. ^{13}C NMR spectrum of obtusiaminocyclin (**24**) (CD_3OD, 150 MHz).

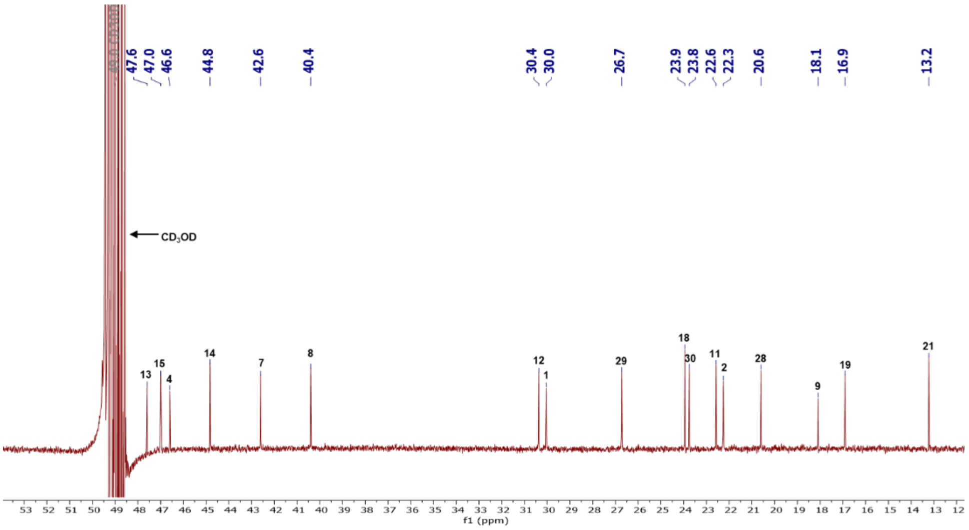

Figure S216. Detail of the ^{13}C NMR spectrum of obtusiaminocyclin (**24**) (CD_3OD, 150 MHz).

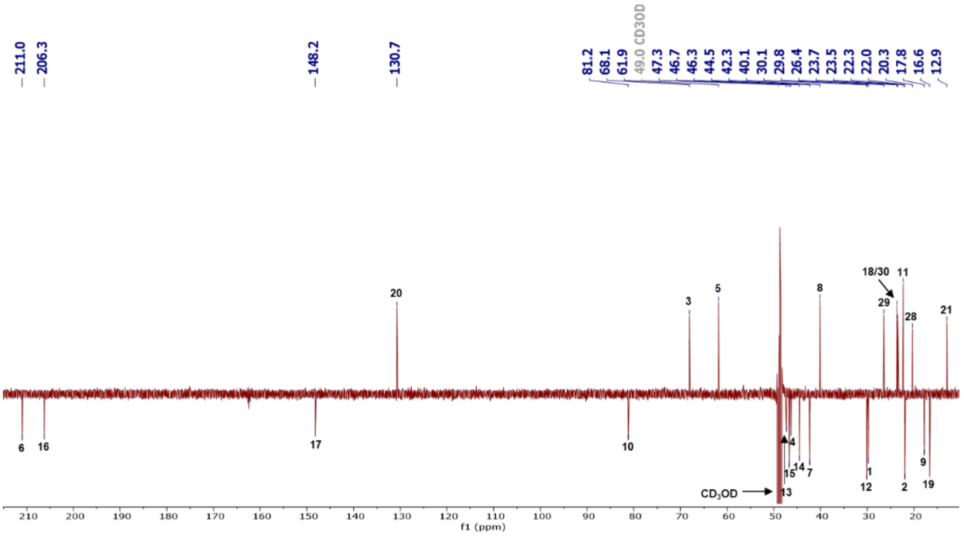

Figure S217. APT spectrum of obtusiaminocyclin (**24**) (CD_3OD, 150 MHz).

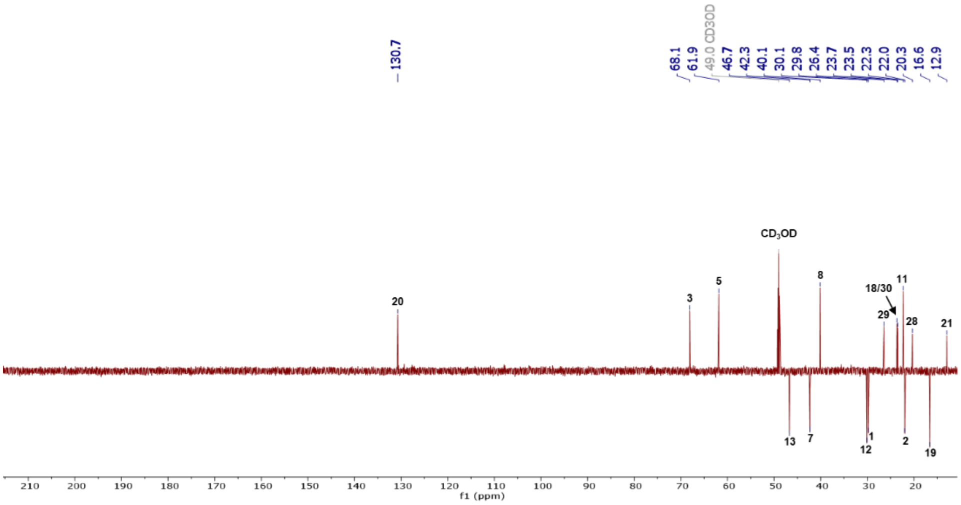

Figure S218. DEPT-135 spectrum of obtusiaminocyclin (**24**) (CD_3OD, 150 MHz).

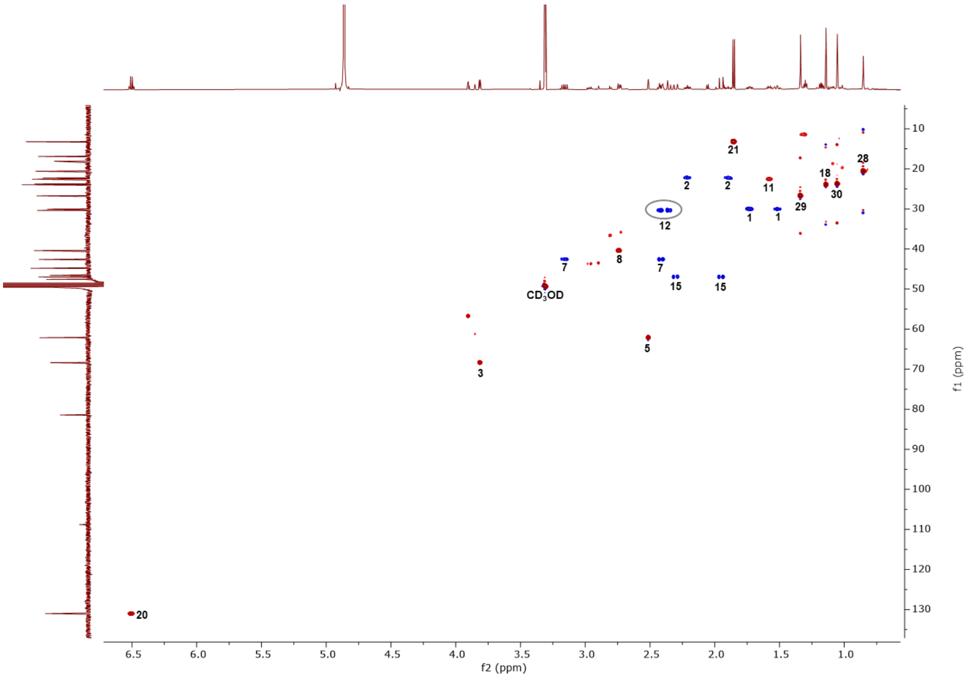

Figure S219. $^{1}H/^{13}C$ HSQC spectrum of obtusiaminocyclin (**24**) (CD_3OD, 600/150 MHz).

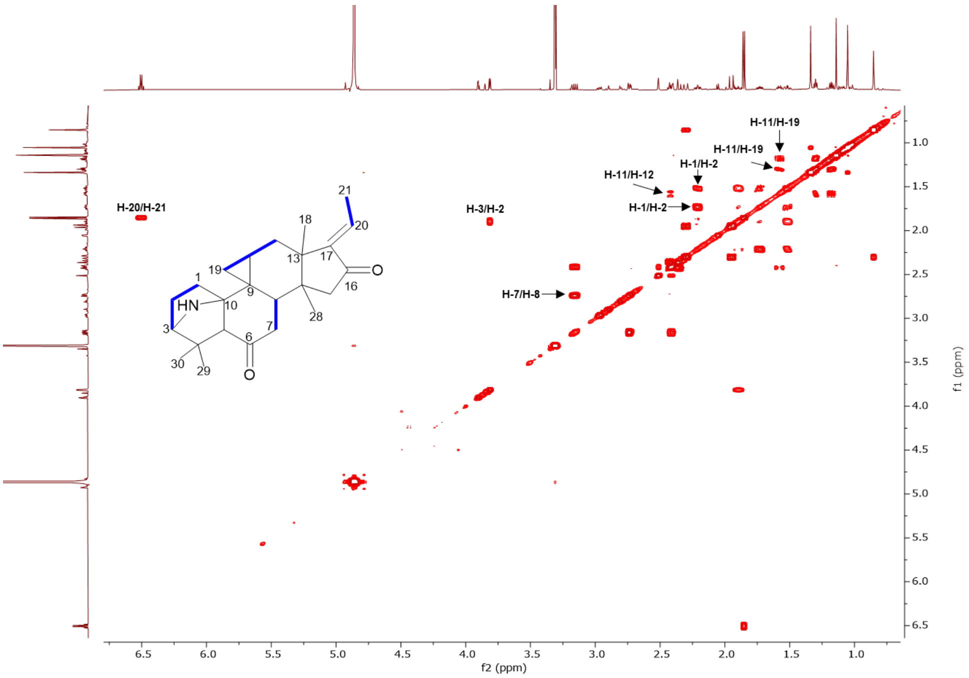

Figure S220. $^1H/^1H$ COSY spectrum (key correlations) of obtusiaminocyclin (**24**) (CD_3OD, 600 MHz).

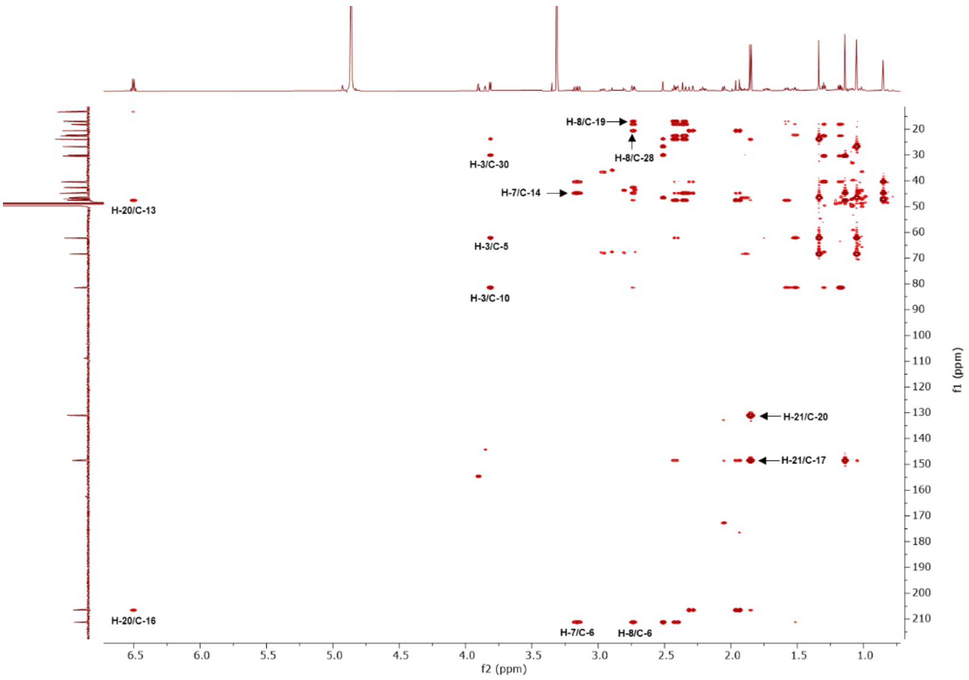

Figure S221. $^1H/^{13}C$ HMBC spectrum (key correlations) of obtusiaminocyclin (**24**) (CD_3OD, 600/150 MHz).

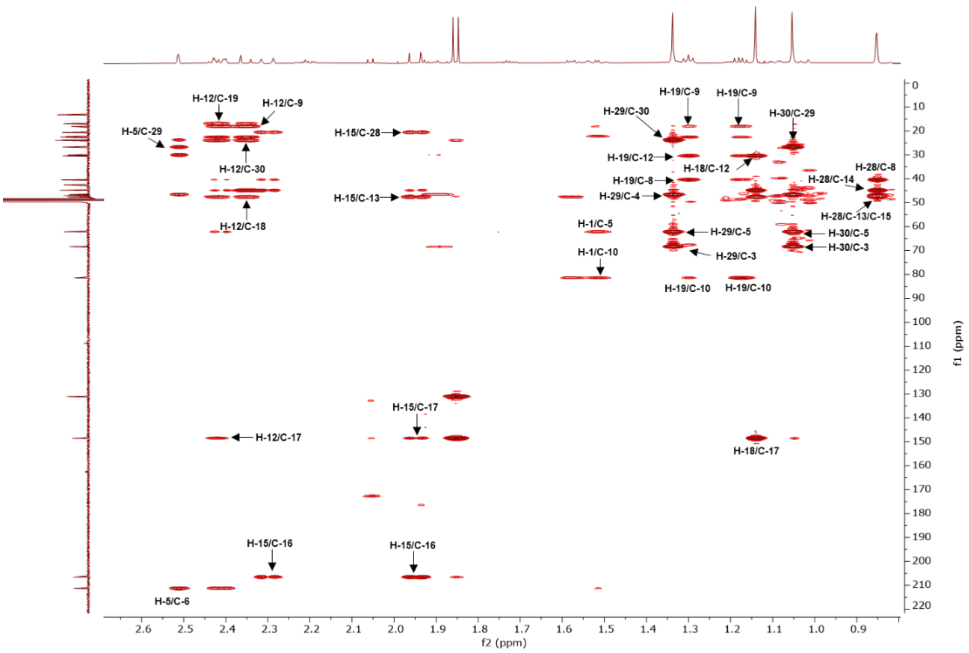

Figure S222. Detail of the $^1H/^{13}C$ HMBC spectrum (key correlations) of obtusiaminocyclin (**24**) (CD_3OD, 600/150 MHz).

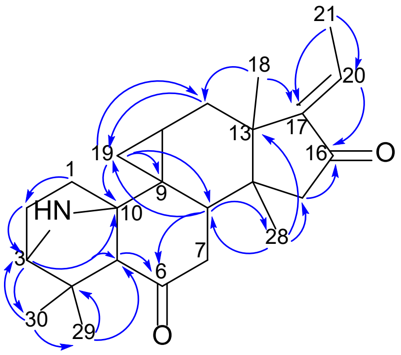

Figure S223. Key HMBC (blue arrows) correlations of obtusiaminocyclin (**24**).

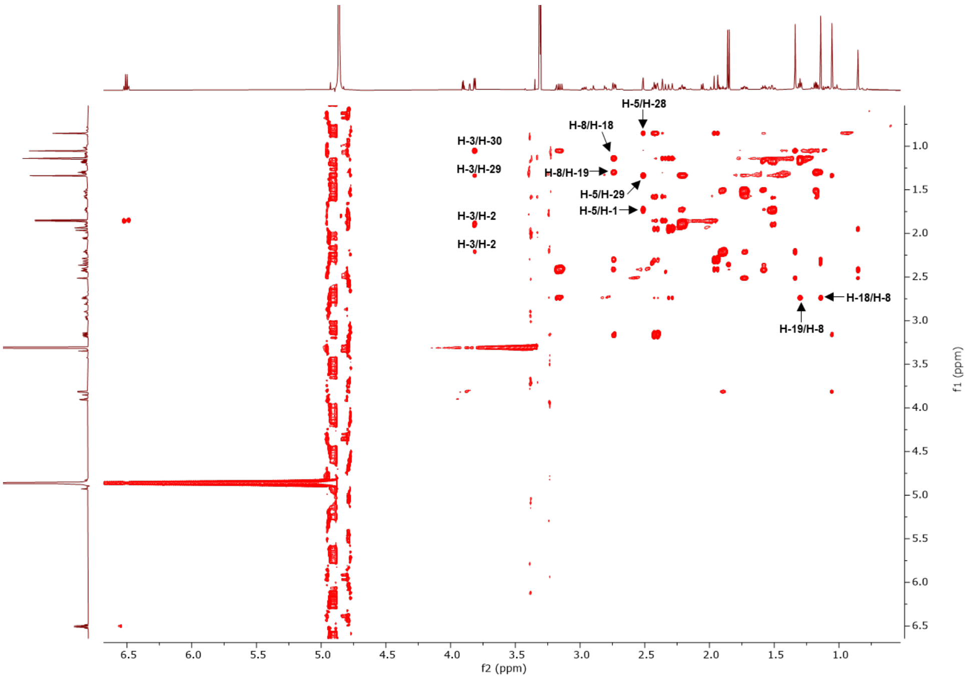

Figure S224. $^1H/^1H$ NOESY spectrum (key correlations) of obtusiaminocyclin (**24**) (CD_3OD, 600 MHz).
